# Adipocyte‐Derived Exosomal miR‐5099 Mitigates M1 Macrophage Polarization and Adipose Inflammation via c‐Met/NF‐κB Axis to Improve Metabolic Health

**DOI:** 10.1002/advs.202518902

**Published:** 2026-02-21

**Authors:** Ping Tang, Lixin Tai, Hongxia Xu, Dongliang Zhu, Jiajia Li, Hao Feng, Jinghui Cui, Shuhai Lin, Li‐jun Di, Li Wang

**Affiliations:** ^1^ Department of Biological Sciences Faculty of Health Sciences University of Macau Macau P. R. China; ^2^ Ministry of Education Frontiers Science Center for Precision Oncology (FSCPO) University of Macau Macau P. R. China; ^3^ State Key Laboratory of Cellular Stress Biology School of Life Science Faculty of Medicine and Life Sciences Xiamen University Xiamen P. R. China

**Keywords:** exosome, inflammation, macrophage, miR‐5099/c‐Met, obesity

## Abstract

Obesity underlies metabolic dysfunction and contributes to the pathogenesis of various diseases. Obesity associated adipose tissue (AT) inflammation contributes to whole body inflammation vulnerability and metabolic disease development. Celastrol (CEL) exhibits significant therapeutic potential against obesity; however, its clinical application is limited by toxicity. Here, CEL treatment fundamentally reprograms the microRNA (miRNA) profile of adipocyte‐derived exosomes. Among the altered miRNAs, we identified miR‐5099 as being dramatically upregulated and enriched specifically within adipocyte‐derived exosomes. We found that CEL‐conditioned adipocyte culture medium exhibited the beneficial effects, including the suppression of M1 macrophage polarization, improvement of metabolic function, and reduction of inflammation in obese animals. Importantly, these beneficial effects are largely dependent on the presence of exosomal miR‐5099. Furthermore, direct administration of miR‐5099 in obese mice significantly ameliorated metabolic disorders, including adipose tissue inflammation and hepatic steatosis. Mechanistically, miR‐5099 attenuates AT inflammation by directly targeting the c‐Met/NF‐κB axis in infiltrated macrophages. Concomitantly, miR‐5099 enhances systemic insulin sensitivity and glucose homeostasis across metabolic tissues. Collectively, our study identifies miR‐5099 as the key downstream effector of CEL. This finding suggests that direct miR‐5099 administration offers a strategy to harness the therapeutic benefits of CEL while circumventing its toxicity, positioning it as a promising treatment for obesity and associated comorbidities.

## Graphical abstract

Celastrol upregulates adipocyte‐derived exosomal miR‐5099, suppressing the macrophage c‐Met/NF‐κB axis to mitigate pro‐inflammatory M1 polarization and adipose inflammation. These miR‐5099‐enriched exosomes also act as endocrine signals targeting the liver, muscle, and adipocytes to significantly enhance systemic insulin sensitivity. This mechanism effectively restores metabolic homeostasis in obesity through both local and systemic delivery of miR‐5099.

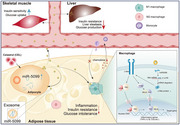

**Abbreviations**
3T3‐M: Matured 3T3‐L1 adipocytes
3T3‐Pre: 3T3‐L1 preadipocytes
ALT: Alanine Transaminase
AST: Aspartate Aminotransferase
AT: Adipose Tissue
ATMs: adipose tissue macrophages
BAT: Brown Adipose Tissue
BMDM: Bone Marrow Derived Macrophage
BUN: Blood Urea Nitrogen
CEL: Celastrol
CEL‐CM: Conditional Medium from CEL treated adipocytes
CEL‐CM‐Sup: Supernatant from the culture medium of CEL treated adipocytes without Exosome
CEL‐EXO: Exosomes derived from CEL treated adipocytes
CEL‐KO‐EXO: Exosomes derived from CEL treated adipocytes with miR‐5099 knockout
CM: Conditional Medium
Ctrl‐CM: Conditional Medium from DMSO treated adipocytes
Ctrl‐CM‐Sup: Supernatant from the culture medium of DMSO treated adipocytes without Exosome
Ctrl‐EXO: Exosomes derived from DMSO treated adipocytes
eWAT: epididymal White Adipose Tissue
EXO: Exosomes
EXO‐OE: Exosomes derived from CEL treated adipocytes with miR‐5099 overexpression
GTT: Glucose Tolerance Test
HFD: High Fat Diet
IFNγ: Interferon‐gamma
iNOS: Inducible Nitric Oxide Synthase
ITT: Insulin Tolerance Test
iWAT: inguinal White Adipose Tissue
LDH: Lactate Dehydrogenase
LPS: Lipopolysaccharide
NC: Negative Control
OA: Oleic Acid
OE: Overexpression
PA: Palmitic Acid
ROS: Reactive Oxygen Species
sg‐miR‐5099: Single guiding RNA for miR‐5099 knockout
SKL: Skeletal muscle
SVF: Stromal Vascular Fraction
TC: Total cholesterol
TEM: Transmission Electron Microscopy
TG: Triglycerides
UTR: Untranslated Region
WAT: White Adipose Tissue

## 1 Introduction

Obesity has emerged as a global health crisis and is recognized as one of the most significant public health challenges of the modern era. Importantly, obesity is a major contributor to mortality worldwide, primarily due to its role in driving many chronic diseases, including metabolic disorders such as insulin resistance, type 2 diabetes, cardiovascular diseases, hypertension, stroke, and even cancer.

Adipose tissue (AT), especially white AT (WAT) contains resident immune cells crucial for monitoring and preserving adipocyte function and hormonal sensitivity [1]. Under lean conditions, healthy AT exhibits an anti‐inflammatory state, characterized by elevated levels of anti‐inflammatory immune cells like regulatory T cells (Tregs) and M2‐polarized adipose tissue macrophages (ATMs). This environment promotes high adipocyte insulin sensitivity, which suppresses lipolytic signals and ensures metabolic flexibility. Conversely, obesity shifts the AT immune milieu toward a pro‐inflammatory state. This shift involves increased infiltration and polarization of pro‐inflammatory immune cells such as M1 macrophages, which secrete inflammatory cytokines such as TNF‐α and IL‐1β [2]. The resulting chronic adipose tissue inflammation is a key driver of insulin resistance and the development of metabolic disorders [1, 3, 4, 5, 6]. Understanding the mechanisms underlying macrophage recruitment, accumulation, and phenotypic transformation in AT is crucial for developing targeted therapies. Interventions targeting this inflammation hold significant promise for preventing or reversing insulin resistance, offering novel therapeutic strategies against diabetes and its comorbidities.

Exosomes are small extracellular vesicles (approximately 150 nm in diameter) enclosed by a phospholipid bilayer membrane, which are produced by various types of cells [7]. Numerous studies have highlighted the dual role of exosomes as both cellular messengers and significant biomarkers reflecting various physiological and pathophysiological states. Due to their natural origin within the body, exosomes can evade immune system detection and clearance. They are also recognized as effective drug delivery vehicles. By targeting specific cells, they enhance therapeutic bioavailability and reduce side effects. These combined advantages, including their signaling role, biomarker value, biocompatibility, and targeted delivery, are driving increased scientific interest in understanding exosome biology and mechanisms. Most mammalian cells produce exosomes with AT as one of the major contributors. Adipocytes‐derived exosome vehicles are considered to have important regulatory functions and mediate the crosstalk between adipose tissue and other organs [8]. Exosomes are enriched with diverse bioactive molecules, including proteins, lipids, DNA, and a variety of RNA types, facilitating intercellular communication and pivotal roles in numerous biological processes [9]. Among these molecules, miRNA has particularly captured significant interest in recent research endeavors [10].

Exosome‐delivered miRNAs have been demonstrated to possess significant biological functions. Research underscores the potential of exosomal miRNAs as biomarkers in obesity, type 2 diabetes, and related metabolic disorders. Distinct profiles in blood and adipose tissue show differential expression of hundreds of miRNAs between lean and obese individuals, reflecting their role in disease development and progression. Changes in these profiles, particularly from adipose tissue in obesity, correlate with key pathophysiological features like glucose intolerance, insulin resistance, and inflammation. Circulating exosomal miRNAs emerge as promising non‐invasive tools for early detection and disease monitoring. Research in animal models has demonstrated the therapeutic potential of numerous miRNAs in combating obesity, preventing glucose intolerance, and addressing insulin resistance. Some miRNA‐based therapies are already undergoing clinical trials for various diseases, including neurocognitive disorders, cancer, and metabolic conditions like obesity, type 2 diabetes, non‐alcoholic fatty liver disease (NAFLD), and non‐alcoholic steatohepatitis (NASH) [11]. Notably, Adipose tissue is a key regulator of circulating miRNAs. In conditions such as obesity and lipodystrophy, miRNA expression within adipocytes is altered. This dysregulation may subsequently lead to changes in the profile of extracellular miRNAs. The majority of circulating microRNAs encapsulated in exosomes originate from adipocytes and are released into the extracellular space. These miRNAs travel to distant tissues like the liver, muscle, lung, and pancreas, exerting their effects at remote sites by regulating target gene expression, thereby influencing inflammation, insulin sensitivity, and metabolic balance [9, 12, 13, 14]. These miRNAs derived from adipocytes are thought to act locally, influencing the function of recipient cells, such as macrophages in adipose tissue, to regulate inflammation and metabolic health within the adipose tissue [15, 16]. Targeting these miRNAs involved in cell‐to‐cell communication with anti‐obesity miRNA‐loaded carriers is believed to offer potential for mitigating both obesity and adipose tissue inflammation in clinical contexts. These results emphasize the considerable potential of exosomal miRNAs as therapeutic interventions for metabolic disorders, necessitating additional exploration of their mechanisms of action.

Tripterygium wilfordii, native to China, has been recognized for its toxicity for over a millennium. This is evident in its traditional names, which carry strong cautionary meanings, such as “Duanchang Cao” (meaning “gut‐breaking herb”) and “Shan Pishuang” (“Pishuang” refers to the highly toxic arsenic trioxide, known for its extreme lethality). Celastrol (CEL) is a quinone methide triterpenoid compound extracted from the root of Tripterygium wilfordii, which is one of the most bioactive components of the plant. It has been extensively studied for its wide range of pharmacological effects, including antitumor, anti‐inflammatory, immunosuppressive, and anti‐obesity properties. CEL has been proved to be a powerful anti‐obesity compound and causes dramatic body weight loss in animal models [17]. The well proven working mechanism of CEL in body weight control is through increasing the leptin sensitivity and decreasing food intake [17, 18]. Other mechanisms involve increased mitochondrial function in fat and muscle tissues were also proposed with some argument [18, 19]. More intriguingly, another independent study observed a moderate but significant body weight reduction in nonobese mice and LepR null mice by CEL treatment (100ug/kg) [20], suggesting the mechanism of CEL mediated body weight reduction remains largely unknown. However, direct administration of CEL has been associated with undesirable side effects, limiting its clinical applicability [21]. Identifying downstream targets of CEL that mediate its therapeutic benefits while minimizing toxicity could enable the development of novel therapeutic strategies against metabolic disorders.

Our study unveils that CEL, acting on adipocytes, triggers the production of a novel microRNA‐5099 (miR‐5099). This miRNA is then encapsulated in exosomes and released into adipose tissue to influence neighboring macrophages around adipocytes within the tissue. MiR‐5099 inhibits the c‐Met gene in pro‐inflammatory macrophages, inhibiting NF‐κB pathway, hindering M1 macrophage polarization, and decreasing the production of inflammatory cytokines. This process mitigates obesity‐induced inflammation, insulin resistance, and metabolic disorders. The discovery of this “adipocyte/exosome/miR‐5099/macrophage axis” mechanism highlights exosomal miR‐5099 as a therapeutic agent. It replicates the benefits of CEL treatment while avoiding its direct adverse effects. This offers a promising and targeted strategy for combating obesity‐related metabolic syndrome.

## 2 Results

### 2.1 Direct CEL Administration Ameliorates Metabolic Disorders but Induces Toxic Adverse Effects in Obese Mice

**FIGURE 1:**
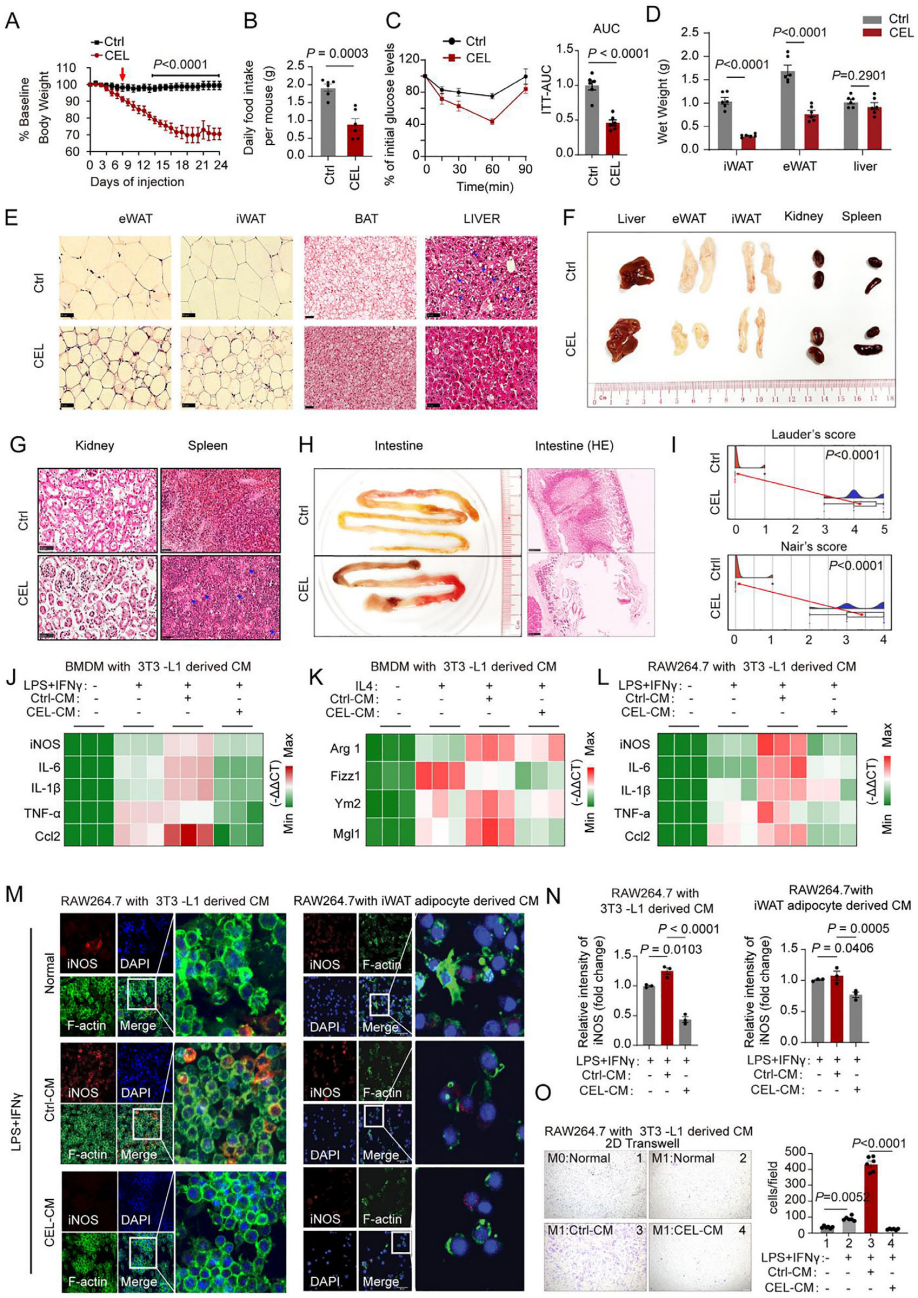
Assessing the impact of Celastrol (CEL) on metabolic health and its influence on adipocyte‐derived conditioned media (CM) in modulating macrophage activation. Male C57Bl/6J mice (6–8 weeks) were induced to obesity by a high‐fat diet (HFD) for 8 weeks, followed by a 21‐day daily injection of CEL (intraperitoneal daily injections with indicated doses), these mice were used for the experiments in Figure 1A–I (n = 6). (A–B) Body weight change (A) and food intake (B) during a 21‐day CEL treatment. Arrows indicate dose escalation (0.1→0.5 mg/kg/day). (C) Insulin tolerance test (ITT) analysis and area under the curve (AUC) for ITT of CEL treated mice. (D) Final weights of eWAT, iWAT, and liver of the mice. (E‐G) H&E staining of iWAT, eWAT, liver (E), spleen, kidney (G), and representative images of these tissues (F).scale bar, 50 µm. (H‐I) Representative images and histological images of intestines (H, scale bar, 250 µm) and adhesion scores (I). (J‐L) Heatmaps analysis of the expression of M1 marker genes (J) and M2 marker genes (K) in BMDMs and M1 marker genes in RAW264.7 cells (L) cultured in conditional medium (CM) collected from 3T3‐1 adipocytes without or with CEL treatment (400 nm, 48 h) (n = 3). (M‐N) Immunofluorescence (IF) staining for iNOS, F‐actin (for cellular morphological control), and DAPI in LPS/IFNγ (100 ng/mL;20 ng/mL, 24 h) induced RAW264.7 M1 macrophages cultured in the presence of PBS or conditioned media (CM) obtained from 3T3‐L1 adipocytes or iWAT SVF‐differentiated adipocytes (iWAT adipocytes, (M) and the quantification for iNOS staining (N), with or without CEL treatment. Scale bar, 50 µm. (O) Migration analysis and quantification of RAW264.7 M0 and M1 macrophages cultured in CM harvested from 3T3‐adipocytes with or without CEL treatment (n = 6). Scale bar for O, 200 µm. Comparison between the two groups was evaluated using an unpaired two‐tailed *t*‐test. One‐way ANOVA with Tukey's post‐hoc test was used for comparing more than two groups. Data represent mean ± SEM.

First, we tested the effects of direct CEL administration on the metabolic health of obese animals. We observed the dramatically reduced body weight of DIO (high fat diet induced obese) mice with 0.5 mg/kg/d CEL treatment (Figure 1A). CEL treated DIO mice showed a significant reduction in both the daily food intake and ITT (Insulin tolerance test) than the control mice (Figure 1B,C). At the tissue level, the weight reduction is mainly contributed by decreased inguinal white adipose tissue (iWAT) and epididymal white adipose tissue (eWAT) but not liver (Figure 1D; Figure S1A). The adipocytes from iWAT, eWAT, and BAT tissues of CEL treated mice showed smaller sizes (Figure 1E; Figure S1B). Consistently, liver histology demonstrated a marked decrease in liver steatosis in DIO mice treated with CEL (Figure 1E; Figure S1C). Furthermore, CEL‐treated DIO mice showed lowered serum levels of total cholesterol (TC), albeit not triglycerides (TG) (Figure S1D).

Although we observed decreased fat tissues (both iWAT and eWAT) with CEL treatment (Figure 1F), significant side effects on other organs were also noted. While CEL treatment did not induce hepatotoxicity, as evidenced by insignificant change of ALT (Alanine Transaminase) and AST (Aspartate Aminotransferase) levels, the elevated serum levels of BUN (Blood Urea Nitrogen) and LDH (Lactate Dehydrogenase) indicate potential nephrotoxicity and general cytotoxicity associated with the treatment (Figure S1E). Consistently, obese animals treated with CEL exhibited organomegaly, notably affecting the spleen and kidney (Figure 1F). Histological examination and scoring revealed extensive cell depletion in the spleen and altered cell morphology in the kidneys (Figure 1G; Figure S1F). Notably, the most striking organ pathology was observed in the intestine, showing extensive ulceration and mucosal erosion based on histological analysis and damage scoring (Figure 1H,I; Figure S1F). Furthermore, CEL treatment led to anus irritation and internal tissue adhesions, as evidenced by histological images and the scoring of histological damage (Figure S1G; Figure 1I). Collectively, these findings demonstrate CEL's severe systemic toxicity in visceral organs, which precludes the clinical application of direct CEL administration.

Next, we attempted to design strategies that take advantage of its beneficial aspects while mitigating the detrimental ones. As obesity‐induced AT inflammation significantly contributes to obesity induced metabolic disorders, targeting the communication between adipocytes and inflammatory adipose tissue macrophages (ATM) could be a promising strategy to reduce inflammation and associated insulin resistance in obesity. Hence, our investigation aimed to identify downstream factors that mediate CEL's impact on the communication between adipocytes and macrophages. We first investigated the impact of conditioned medium (CM) obtained from CEL‐treated adipocytes on macrophage polarization. Remarkably, we observed that the control CM (Ctrl‐CM), derived from matured 3T3‐L1 adipocytes with vehicle treatment, significantly enhanced M1 polarization induction by LPS/IFNγ on bone marrow‐derived macrophages (BMDMs). This was evidenced by an increased expression of M1 marker genes. In contrast, the CM derived from CEL‐treated 3T3‐L1 adipocytes (CEL‐CM) significantly inhibited the expression of M1 marker genes induced by Ctrl‐CM (Figure 1J; Figure S1H). We also observed a similar effect of CEL‐CM on the M2 macrophage gene program induced by Ctrl‐CM. However, the impact of Ctrl‐CM on the gene expression profile of M2 cells was inconsistent, with some genes showing an increase and others a decrease (Figure 1K; Figure S1I). CEL‐CM also suppressed Ctrl‐CM‐stimulated M1 polarization in RAW264.7 macrophages under LPS/IFNγ induction (Figure 1L). This effect was observed using CEL‐CM from two sources: 3T3‐L1 adipocytes and primary adipocytes. These primary adipocytes were differentiated in vitro from the stromal vascular fraction (SVF) of inguinal white adipose tissue (iWAT). The CEL‐CM downregulated M1 marker gene expression. It also reduced M1 marker iNOS staining and the number of inflammatory CD11c^+^ cells (Figure 1M,N; Figure S1J–M). Emerging evidence suggests that the increased migration and subsequent accumulation of macrophages, particularly M1 macrophages in AT, play a significant role in AT inflammation. This inflammation, in turn, exacerbates metabolic complications associated with obesity [22, 23]. Our observations revealed that CEL‐CM obtained from both 3T3‐L1 and primary iWAT adipocyte cultures significantly diminished the transmembrane migration ability of M1 macrophages when compared to macrophages cultured with Ctrl‐CM, as demonstrated in the transwell assay (Figure 1O; Figure S1N). This indicates that CEL‐CM negatively influences the migratory capabilities of macrophages, consequently influencing adipose tissue inflammation. These findings suggest that mature adipocytes may release factors that drive macrophage differentiation and polarization. However, CEL treatment appears to dampen this effect, potentially disrupting the crosstalk between adipocytes and macrophages.

Remarkably, direct exposure to CEL within a non‐cytotoxic dose range (Figure S2A) exhibited a dose‐dependent repression of M1 macrophage polarization in BMDMs. This was supported by a notable reduction in the expression of M1 marker genes and iNOS staining (Figure S2B–E). Surprisingly, CEL treatment also suppressed the expression of M2 macrophage marker genes during IL‐4‐induced M2 polarization (Figure S2F). Consistent with these in vitro findings, serum proteomic analysis from CEL‐treated mice revealed a global reduction in proteins associated with immune and inflammatory responses (Figure S2G–I). Taken together, these findings illustrate that CEL exerts a broad inhibitory effect on macrophage polarization and maturation, consequently dampening the immune function systemically. This suggests that direct CEL administration could induce a state of immune suppression, potentially exacerbating tissue damage by compromising host defense and repair mechanisms. Therefore, selectively interrupting adipocyte macrophage communication (e.g., via CEL‐primed adipocyte signals), may offer greater therapeutic potential than direct CEL administration.

### 2.2 CEL Treated Adipocyte‐Derived Exosome Modulates M1 Macrophage Polarization

**FIGURE 2:**
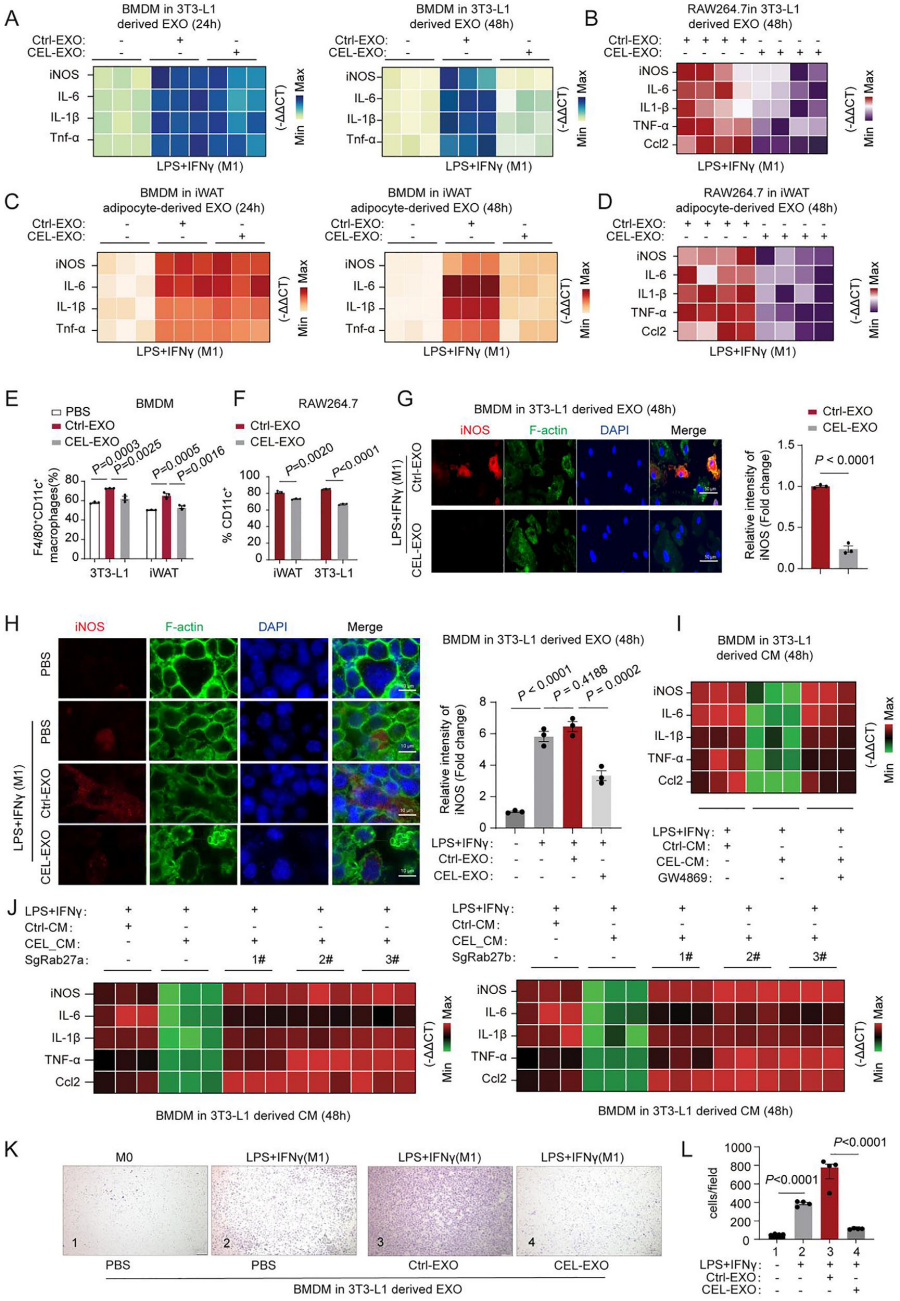
CEL‐treated adipocyte‐derived exosomes suppress M1 macrophage polarization and migration via EXO‐dependent mechanisms. (A–D) Heatmaps analysis of the expression of M1 macrophage markers in BMDM or RAW264.7 M1 macrophages following treatment with exosomes (EXOs) (50 µg/mL, 24 h) collected from the culture medium of 3T3‐L1 adipocytes (A, B) with or without CEL treatment for 24, or 48 h, as well as from iWAT adipocytes (C, D) with or without CEL treatment for 24, or 48 h (n = 3). (E–F) Flow cytometry analysis of F4/80^+^CD11C^+^ BMDM macrophages (E) and CD11C^+^ RAW264.7 macrophages (F) following treatment with EXOs (50 µg/mL, 24 h) collected from the culture medium of 3T3‐L1 adipocytes or iWAT adipocytes with or without CEL treatment (0.4 µm, 48 h) (n = 3). (G–H) IF staining for iNOS, F‐actin, and DAPI in BMDMs (G) and RAW264.7 macrophages (H) as depicted in E and F. Scale bar for G, 50 µm, Scale bar for H, 10 µm (n = 3). (I‐J) Heatmap of M1 marker expression in BMDM macrophages cultured in CM (24 h) harvested from 3T3‐L1adipocytes with or without CEL treatment (0.4 µm, 48 h), combined with the treatment of exosome secretion inhibitor GW4869 (20 µm, 24 h, I), or with Rab27a and Rab27b knockout (J) (n = 3). (K–L) Migration analysis using transwell (K) and quantification (L) in M0 and M1 BMDMs treated with EXOs (30ug, 24 h) obtained from 3T3‐L1 adipocytes with or without CEL treatment (0.4 µm, 48 h) (n = 4). Scale bar for K, 200 µm. Comparison between the two groups was evaluated using an unpaired two‐tailed *t*‐test. One‐way ANOVA with Tukey's post‐hoc test was used for comparing more than two groups. Data represent mean ± SEM.

We next examined which components of the CEL‐CM regulate macrophage polarization. Emerging evidence highlights that adipocyte‐derived extracellular vesicles, particularly exosomes constitute a large fraction of the AT secretome. These vesicles play a crucial role in facilitating intercellular communication and modulating metabolic complications associated with obesity. Therefore, we isolated exosomes from CM obtained from in vitro cultured 3T3‐L1 or primary adipocytes (from iWAT) without or with CEL treatment to investigate the impact of adipocyte‐derived exosomes on macrophage polarization (Ctrl‐EXOs and CEL‐EXOs, Figure S3A). We initially characterized both Ctrl‐EXOs and CEL‐EXOs using Nanosight, confocal, and transmission electron microscopy (TEM) (Figure S3B). TEM imaging confirmed the characteristic cup‐shaped morphology of the exosomes (Figure S3B). Nanoparticle Tracking Analysis (NTA) further revealed the size distribution and concentration profiles of both exosome groups (Figure S3C). Western blot analysis validated the exosomal identity through positive markers (HSP70, Flotillin, CD9, and CD63) and the absence of the negative marker Calnexin (Figure S3D). Additionally, IF and flow cytometry analysis of Dil‐labeled EXOs confirmed their uptake by macrophages (Figure S3E,F). Interestingly, exosomes derived from CEL‐conditioned media (CEL‐EXOs), obtained from matured 3T3‐L1 adipocytes or primary adipocytes, exhibited a notable ability to inhibit the polarization of BMDMs or RAW264.7 macrophages toward the M1 phenotype when compared to control exosomes, Ctrl‐EXOs. This inhibitory effect was observed to be time‐dependent, with the most significant impact seen at the 48‐h time point. This was evidenced by the suppression of the M1 marker gene program, decrease in the percentage of CD11c+ cells, and reduced iNOS staining in BMDMs and RAW264.7 macrophages (Figure 2A–H; Figure S4A–F). However, notably, we did not observe a substantial and significant impact of these exosomes on M2 macrophage polarization. This was determined by assessing M2 marker gene expression and the percentage of F4/80^+^Mgl1^+^ (M2) macrophages (Figure S4G–I). In obese adipose tissue, macrophages increase reactive oxygen species (ROS) production, driving their progression toward a pro‐inflammatory M1 phenotype, ultimately triggering inflammation and insulin resistance [24, 25, 26]. Our data revealed that Ctrl‐EXOs derived from mature 3T3‐L1 adipocytes significantly potentiated ROS generation in LPS/IFNγ‐stimulated RAW264.7 M1 macrophages. In striking contrast, CEL‐EXOs effectively attenuated this ROS elevation (Figure S5A–C). Importantly, this ROS‐modulating capacity was specifically attributable to the exosomal fraction, as depletion of exosomes from the CEL‐CM (CM‐supernatant) completely abolished these regulatory effects (Figure S5D,E). This CEL‐EXO specific effect on ROS reduction was also observed in LPS/IFNγ‐stimulated mouse BMDM M1 macrophages (Figure S5F–I) and human THP1 M1 macrophages (Figure S5J). Subsequent examination of iNOS staining and M1 marker gene expression further affirmed the specific inhibitory effect of CEL‐EXOs on M1 macrophages. This effect was validated when BMDMs and RAW264.7 macrophages were cultured in CM‐sup with exosomes removed (Figure S5K,L). To confirm the role of exosomes, we treated 3T3‐L1 adipocytes with GW4869, a potent exosome inhibitor. This treatment significantly reduced the ability of CEL‐CM to suppress M1 polarization in BMDMs. Consequently, the expression of M1 marker genes was restored to higher levels (Figure 2I; Figure S6A). Upon knocking down Rab27a and Rab27b, crucial regulators of exosome secretion, in 3T3‐L1 adipocytes (Figure S6B), the CEL‐CM's potential to suppress M1 macrophage polarization in both RAW264.7 and BMDM cells was also attenuated (Figure 2J; Figure S6C–E). These findings demonstrate that adipocyte‐derived exosomes play an essential role in mediating the inhibitory effects of CEL‐CM on Ctrl‐CM‐induced M1 macrophage polarization. Notably, 3T3‐L1‐derived CEL‐EXO treatment significantly reduced the migration of BMDMs and RAW264.7 macrophages. This suppression was confirmed in transwell assays compared to Ctrl‐EXOs (Figure 2K,L; Figure S6F,G). These results suggest that CEL‐EXOs effectively inhibits the infiltration of M1‐polarized macrophages into adipose tissue. The results indicate that CEL‐CM suppresses M1 polarization primarily through exosome‐mediated mechanisms in adipose tissue. Adipocyte‐derived exosomes facilitate communication between adipocytes and macrophages, thereby modulating macrophage polarization. Treatment with CEL alters the composition of these exosomes, impairing intercellular communication and subsequently affecting M1 macrophage polarization.

### 2.3 Protective Potential of Adipocyte‐Derived CEL‐EXOs against HFD‐Induced Obesity Development and Associated Metabolic Complications

**FIGURE 3:**
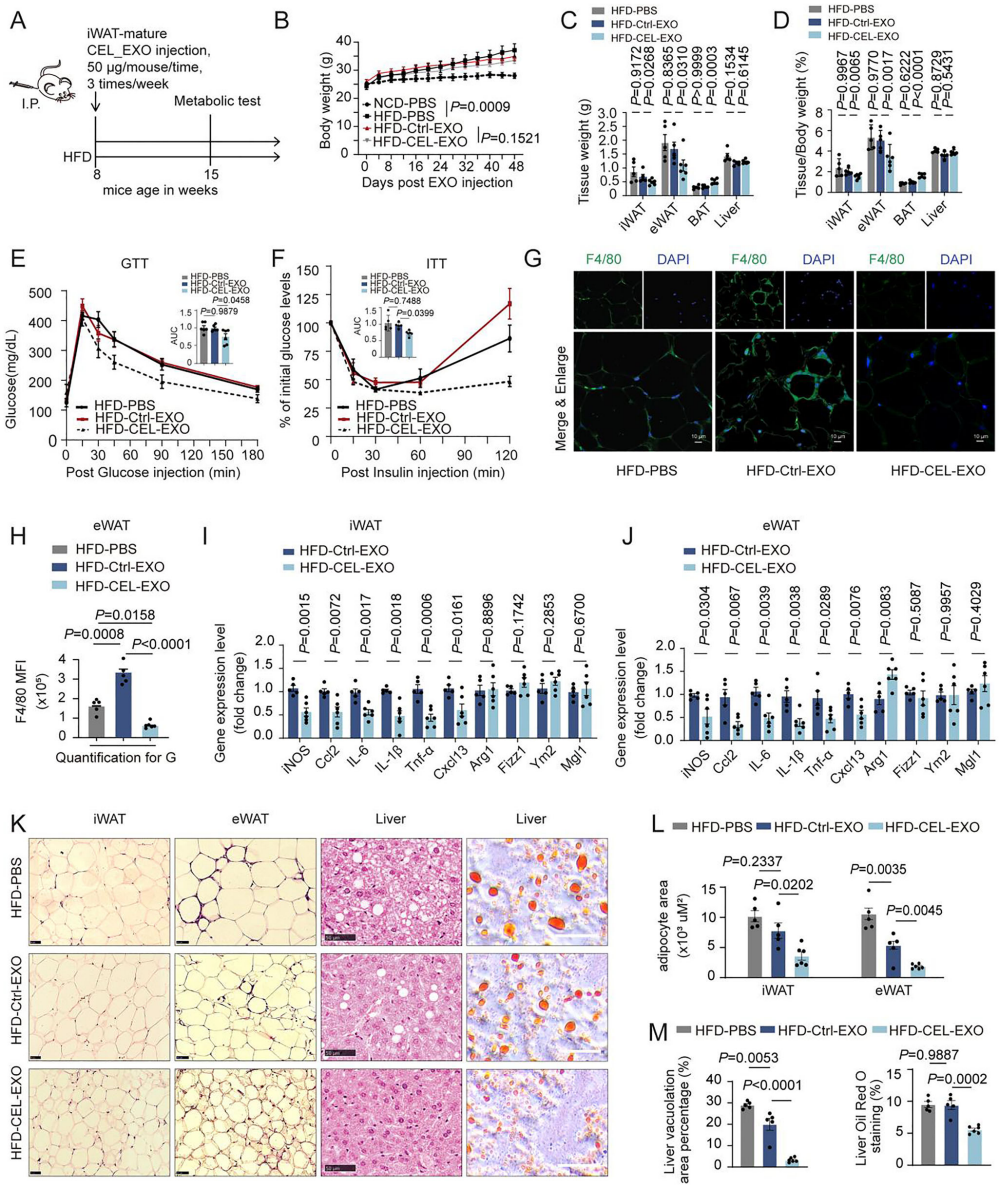
CEL‐treated adipocyte‐derived exosomes improve metabolic health during HFD induced obesity development. (A) Schematic of the dietary and treatment approach to generate experimental mice. Male C57Bl/6J mice (8 weeks) were fed an HFD for 7 weeks with administration of either Vehicle (PBS), Ctrl‐EXOs, or CEL‐EXOs (intraperitoneal injections, 50 µg/mouse, 3times/week) harvested from iWAT adipocytes with or without CEL treatment (0.4 µm, 48 h). Additionally, a control group of mice was fed a normal chow diet (NCD). These mice were utilized for the experiments. (B) Body weight changes over time, and the AUC for body weight. (C–D) Tissue mass (C) and tissue mass as a percentage of body weight at sacrifice (D) of eWAT, iWAT, BAT, and liver (n = 5‐6). (E–F) Glucose tolerance test (GTT, E), insulin tolerance test (ITT, F), and associated AUC (n = 5‐6). (G–H) IF staining (G) and quantification (H) of F4/80^+^ in eWAT of obese mice with indicated treatment (n = 5‐6). Scale bar, 10 µm. (I–J) The expression analysis by RT‐PCR for M1 and M2 marker genes in iWAT (I) and eWAT (J) of obese mice with indicated treatment (n = 5‐6). (K) Representative H&E images of iWAT, eWAT, and liver, and Oil Red O staining of liver sections (n = 5‐6). Scale bar, 50 µm. (L) Quantification of adipocyte size in iWAT and eWAT (n = 5‐6). (M) Quantification of liver microvesicular vacuoles and Oil Red O staining for lipid droplets (n = 5‐6). Comparison between the two groups was evaluated using an unpaired two‐tailed *t*‐test. One‐way ANOVA with Tukey's post‐hoc test was used for comparing more than two groups. Data represent mean ± SEM.

We next investigated whether CEL‐EXOs could prevent high‐fat diet (HFD)‐induced obesity and associated metabolic complications. To test this, mice on the HFD were subjected to intraperitoneal (I.P.) injections of either CEL‐EXOs or Ctrl‐EXOs (derived from iWAT SVF) over 15 weeks (3 × 50 µg/mouse/week, Figure 3A). The detection of exosomes in adipose tissue and other organs indicated the systemic uptake of the administered exosomes (Figure S7A,B). Surprisingly, both CEL‐EXO and Ctrl‐EXO treatments reduced the body weight of obese animals. CEL‐EXOs did not significantly affect body weight or food intake compared to Ctrl‐EXOs (Figure 3B; Figure S7C,D). However, it reduced iWAT and eWAT mass while increasing BAT (Figure 3C,D). Notably, CEL‐EXO treatment markedly enhanced both glucose tolerance and insulin sensitivity (Figure 3E,F). These findings suggest that while both treatments influenced body weight similarly, CEL‐EXOs exerted distinct metabolic benefits by modulating adipose tissue composition and improving glucose homeostasis. Next, we further examined whether CEL‐EXO treatment could affect macrophage accumulation in adipose tissue during the development of obesity. Our results showed that CEL‐EXOs from iWAT SVF‐derived adipocytes decreased macrophage accumulation in obese eWAT compared to Ctrl‐EXOs, indicated by reduced F4/80 staining (Figure 3G,H) and lowered expression of pro‐inflammatory M1 macrophage marker genes in iWAT and eWAT (Figure 3I,J). Moreover, M2 marker genes, including Mgl1, Ym2, and Fizz1 remained largely unchanged, although Arg1 was slightly elevated in eWAT (Figure 3I,J). Interestingly, some M1 marker genes were also observed to be downregulated in BAT and liver following CEL‐EXO treatment as opposed to Ctrl‐EXOs (Figure S7E,F). Furthermore, CEL‐EXOs decreased adipocyte size in WAT depots (Figure 3K,L) and ameliorated HFD‐induced hepatic steatosis (Figure 3K,M). Collectively, these findings demonstrate that CEL‐EXOs confers comprehensive protective effects against HFD‐driven obesity and its metabolic health by mitigating adipose tissue inflammation, particularly targeting M1 macrophage characteristics, reducing adipocyte hypertrophy, and improving liver health.

We further observed that CEL‐EXO treatment enhanced insulin‐stimulated glucose uptake in both 3T3‐L1 adipocytes and C2C12 myocytes (Figure S7G,H). Additionally, in HepG2 and Huh7 hepatocytes, compared to Ctrl‐EXOs, CEL‐EXOs significantly blunted glucagon‐induced hepatic glucose production (Figure S7I,J) and mitigated lipid accumulation, as evidenced by Bodipy staining (Figure S7K–N). These findings indicate that conditioned medium from CEL‐treated adipocytes contains bioactive components. These factors act locally on macrophages and adipocytes. They also enter the circulation to benefit muscle and hepatocytes. Collectively, these multi‐tissue effects improve glucose metabolism and insulin sensitivity.

### 2.4 Therapeutic Potential of Adipocyte‐Derived CEL‐EXOs in Established Obese Animals

**FIGURE 4:**
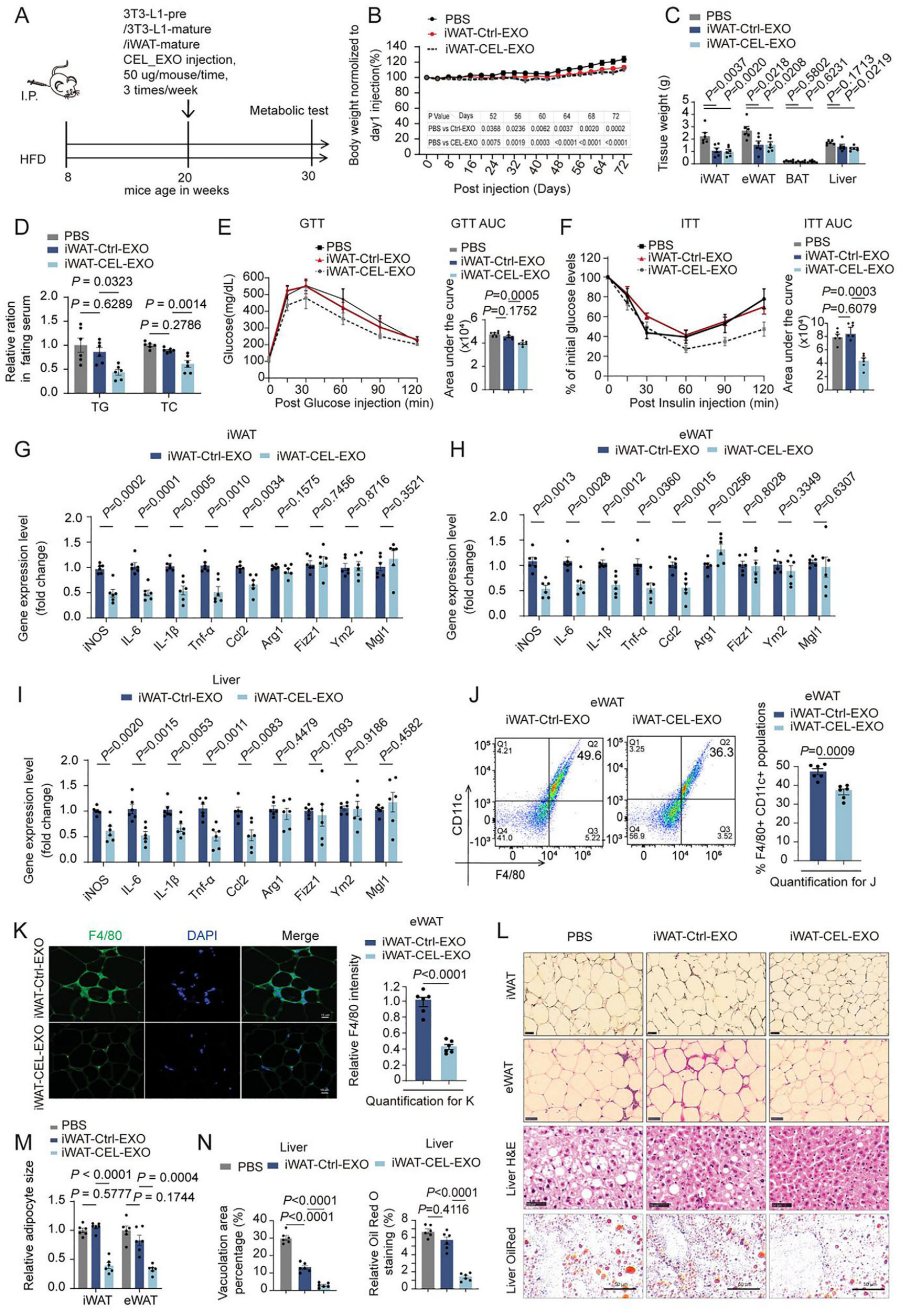
CEL‐treated adipocyte‐derived exosomes ameliorate obesity‐associated metabolic dysfunction and adipose tissue inflammation in obese mice. (A) Schematic of the dietary and treatment approach to generate experimental mice. Male C57Bl/6J mice (8 weeks) were fed an HFD for 12 weeks, followed by the administration of Vehicle (PBS), Ctrl‐EXOs, or CEL‐EXOs (Intravenous (I.V. injections, 50 µg/mouse, 3 times/week) for an additional 10 weeks. The exosomes (EXOs) used in this study were derived from iWAT SVF‐differentiated adipocytes, 3T3‐L1 preadipocytes (3T3‐Pre), or 3T3‐L1 mature adipocytes (3T3‐M), with or without CEL treatment (0.4 µm, 48 h). These mice were utilized for experiments in Figure 4; Figures S8 and 9 (n = 6). (B) Body weight changes over time in obese mice with indicated treatment (n  =  6). (C) Tissue mass of iWAT, eWAT, BAT, and liver in the indicated mice. (D) Fasting serum triglyceride (TG) and total cholesterol (TC) levels. (E‐F) GTT (E), ITT (F), and associated AUC. (G‐I) The expression analysis by RT‐PCR for M1 and M2 marker genes in iWAT (G), eWAT (H), and liver (I) of obese mice with indicated treatment. (J) Flow cytometry plots and quantification of CD11c^+^F4/80^+^ macrophages in eWAT. (K) IF staining and quantification for F4/80^+^ macrophages in eWAT. Scale bar, 10 µm. (L‐N) H&E staining for iWAT, eWAT, and liver, and Oil Red O staining for liver (L); quantification for adipocyte size (M) and for liver microvesicular vacuoles and lipid droplets (N) by ImageJ. Scale bar for L, 50 µm. Comparison between the two groups was evaluated using an unpaired two‐tailed *t*‐test. One‐way ANOVA with Tukey's post‐hoc test was used for comparing more than two groups. Data represent mean ± SEM.

We assessed if the in vitro anti‐inflammatory effects of CEL‐EXOs on macrophages could improve adipose tissue inflammation and metabolic health in obese mice with established metabolic disorders. This would demonstrate the potential of CEL‐EXOs potential not only for preventing obesity development but also for treating existing obesity. Established obese mice (on HFD for 12 weeks) were administered with CEL‐EXOs and Ctrl‐EXOs (derived from iWAT SVF) (Figure 4A) while maintained on a high‐fat diet (HFD).

We applied three types of exosomes derived from iWAT SVF differentiated adipocytes (iWAT‐EXOs) of lean mice, 3T3‐L1 preadipocytes (3T3‐Pre‐EXOs), and matured 3T3‐L1 adipocytes (3T3‐M‐EXOs) without or with CEL treatment to treat HFD induced obese mice (Figure 4A). EXO treatment significantly reduced normalized body weight (Figure 4B; Figure S8A,B). Specifically, EXOs from 3T3‐L1 preadipocytes and adipocytes decreased total body weight gain (Figure S8C). Regarding fat mass, EXOs from iWAT and 3T3‐L1 preadipocytes reduced both iWAT and eWAT, whereas 3T3‐L1 adipocyte EXOs reduced iWAT only (Figure 4C; Figure S8D,E). Food intake remained unchanged (Figure S8F). No significant variances in metabolic traits, including oxygen consumption, CO2 production, heat production, and total physical activity, were observed between the Ctrl‐EXO and CEL‐EXO groups (Figure S8G–J). Under fasting conditions, the baseline glucose levels exhibited a notable distinction between the vehicle‐treated and EXO‐treated groups (both Ctrl‐ and CEL‐EXO), (Figure S8K,L). Nevertheless, the serum triglyceride (TG) and total cholesterol (TC) levels demonstrated a significant decrease in CEL‐EXO‐treated mice in comparison to the Ctrl‐EXO group under the fasting conditions (Figure 4D). The GTT and ITT assays exhibited improved glucose tolerance and insulin sensitivity in CEL‐EXO‐treated mice as compared to the Ctrl‐EXO group (Figure 4E,F; Figure S9A–D). Furthermore, gene expression analysis illustrated that the M1 macrophage proinflammatory markers were downregulated following iWAT‐CEL‐EXO treatment in both iWAT and eWAT and liver tissues compared to iWAT‐Ctrl‐EXO treatment. However, M2 marker genes, including Mgl1, Ym2, and Fizz1 remained unchanged, although Arg1 slightly increased in eWAT (Figure 4G–I). These observations align with the impact of CEL‐EXOs during the progression of obesity (Figure 3), highlighting its specific targeting of M1 macrophage traits.

Consistent with the downregulation of proinflammatory genes, flow cytometry and immunofluorescence staining revealed a marked reduction in the infiltrating M1 macrophage population within obese eWAT following treatment with iWAT‐CEL‐EXO (Figure 4J,K). Treatment with 3T3‐Pre‐CEL‐EXOs or 3T3‐M‐CEL‐EXOs reduced M1 macrophage accumulation in iWAT and eWAT. This was evidenced by decreased iNOS^+^ and F4/80^+^ staining compared to Ctrl‐EXOs (Figure S9E–L). These results suggest that exosomes from CEL‐treated adipocytes exert significant anti‐inflammatory effects. Histological analysis revealed that CEL‐EXO treatment significantly reduces the size of adipocytes in both iWAT and eWAT tissues (Figure S4L,M and Figure S9M–P). Furthermore, CEL‐EXO treatment notably improved liver steatosis complications (Figure 4L,N; Figure S9M–P). Collectively, these findings suggest a significant therapeutic impact of exosome derived from CEL treated adipocytes in addressing adipose tissue inflammation and enhancing the overall metabolic profile in established obese mice.

### 2.5 CEL‐EXO‐Mediated Delivery of Adipocyte miR‐5099 Modulates M1 Macrophage Polarization In Vitro

**FIGURE 5:**
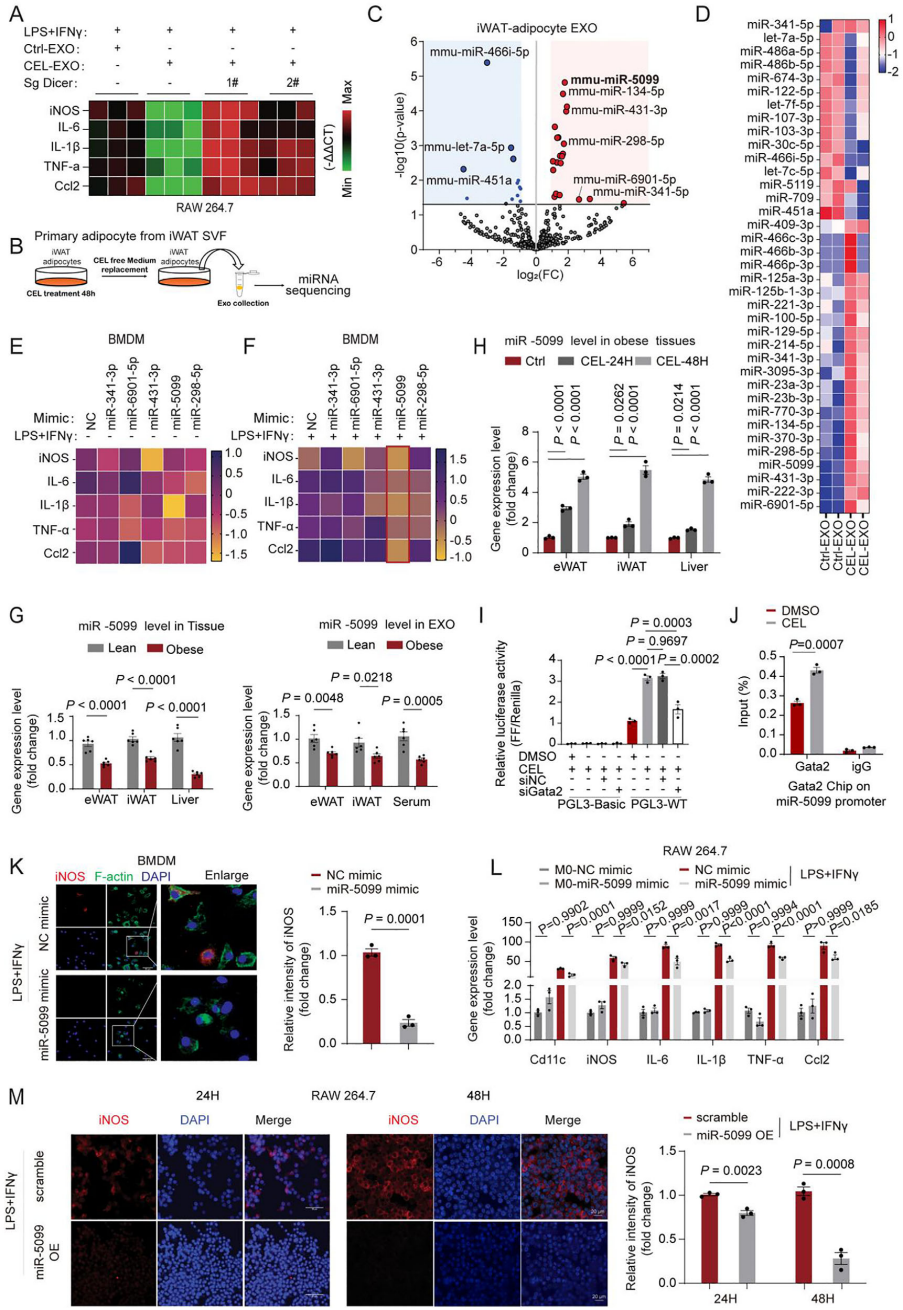
miR‐5099 is enriched in adipocyte‐derived CEL‐EXOs and modulates M1 macrophage polarization. (A) Heatmap illustrates the expression of M1 macrophage marker genes in RAW264.7 M1 macrophages treated with Ctrl‐EXOs or CEL‐EXOs derived from 3T3‐L1 adipocytes subjected to Ctrl or CEL treatment (0.4 µm, 48 h), with or without Dicer knockout (sgDicer) (n = 3). (B) Schematic timeline of exosome treatment and isolation for miRNA sequencing (n = 3). (C–D) Volcano plot (C) and heatmap (D) showing differentially expressed miRNAs extracted from the exosomes obtained from iWAT SVF‐differentiated adipocytes with or without CEL treatment (0.4 µm, 48 h) (n = 3). (E–F) Heatmaps analysis of M1 macrophage marker gene expression in BMDMs treated with indicated miRNA mimics (50 nm, 24 h) in the absence (E) or presence (F) of LPS/IFNγ (10 ng/mL; 20 ng/mL, 24 h) (n = 3). (G) Relative expression level of miR‐5099 in iWAT, eWAT, and liver, as well as in the exosomes derived from iWAT, eWAT, and serum of lean and obese mice (n = 6). (H) Relative expression level of miR‐5099 in iWAT, eWAT, and liver from obese mice treated with or without CEL (0.5 mg/kg BW) for 24 h and 48 h (n = 3). (I) Luciferase reporter assays in 3T3‐L1 cells, without or with CEL (400 nm) treatment, and relative luciferase activities were measured to determine whether CEL can transactivate promoters of miR‐5099 via GATA2‐binding (n = 3). (J) ChIP assay of the GATA2 binding at the promoters of miR‐5099, without or with CEL treatment (n = 3). Nonspecific IgG was used as a negative control for chromatin pulldown. (K) IF staining for iNOS and F‐actin and quantification for iNOS in BMDMs treated with miR‐5099 mimics (50 nm, 24 h) under LPS/IFNγ stimulation (10 ng/mL; 20 ng/mL, 24 h). Scale bar, 50 µm (n = 3). (L) Relative mRNA expression level of M1 macrophage markers in RAW264.7 cells treated with or without LPS/IFNγ (100 ng/mL; 20 ng/mL, 24 h), in combination with or without miR‐5099 mimic treatment for 24 h (n = 3). (M) IF staining for iNOS and quantification in RAW264.7 cells with or without the overexpressing (OE) miR‐5099 under the induction of LPS/IFNγ (100 ng/mL; 20 ng/mL) stimulation for 24 h (left, Scale bar, 50 µm(n = 3)) or 48 h (right, Scale bar, 20 µm(n = 3)). Comparison between the two groups was evaluated using an unpaired two‐tailed *t*‐test. One‐way ANOVA with Dunnet multiple comparison post hoc tests for (H) and Tukey's post‐hoc test for (I & L) were used for comparing more than two groups. Data represent mean ± SEM.

The functionality of exosomes is intricately tied to their cargo, encompassing proteins, mRNAs, non‐coding RNAs, and so on. Despite our efforts to compare the protein profiles between CEL‐EXOs and CTL‐EXOs, we were unable to identify the specific mediators responsible for the observed effects of CEL‐EXOs (Figure S10A,B). We investigated whether microRNAs (miRNAs) mediate these effects. To test this, we silenced Dicer in 3T3‐L1 adipocytes before exosome extraction. We found that Dicer depletion abolished the anti‐inflammatory effects of CEL‐EXOs on M1 macrophages, as confirmed by M1 marker gene expression (Figure 5A; Figure S10C–E). To identify the target miRNAs, we performed deep sequencing of miRNAs extracted from exosomes originating from CEL‐treated vs. Ctrl‐treated adipocytes and identified differentially expressed miRNAs (DEGs) (Figure 5B–D). The top five miRNAs exhibiting the most substantial expression alterations were selected for detailed validation. All five miRNAs affected M1 polarization genes. However, miR‐5099 showed the strongest effect on M1 markers in BMDMs without altering M2 genes (Figure 5E,F; Figure S10F,G).

Tissue distribution analysis unveiled a higher miR‐5099 expression in WAT, especially iWAT, in contrast to other tissues (Figure S10H). Comparative evaluation of miR‐5099 expression in adipocytes and SVF isolated from iWAT indicates a higher miR‐5099 level in the SVF than in adipocytes (Figure S10H). miR‐5099 levels were downregulated in the WAT and liver of obese mice compared to lean controls, and this reduction was mirrored in exosomes isolated from the WAT and serum of obese mice (Figure 5G). However, CEL administration in obese mice upregulated miR‐5099 expression in both WAT and liver (Figure 5H). During 3T3‐L1 adipocyte differentiation, miR‐5099 expression decreased in mature adipocytes relative to preadipocytes. CEL treatment upregulated miR‐5099 expression in both preadipocytes and mature adipocytes, with corresponding elevations observed in exosomes derived from these cell types (Figure S10I). This coordinated increase in cellular and exosomal miR‐5099 was further validated in SVF‐differentiated adipocytes from iWAT following CEL intervention (Figure S10J). We next investigated the mechanism through which CEL stimulates miR‐5099 expression in adipocytes. Analysis of the putative promoter region of miR‐5099 identified a potential GATA2 binding site (TGATAA) located between ‐1067 and ‐1061, suggesting this site may be important for miR‐5099 promoter activity. Notably, CEL was found to significantly increase GATA2 expression in 3T3‐L1 preadipocytes (Figure S10K). To determine whether GATA2 mediates CEL‐induced regulation of miR‐5099, we performed promoter‐luciferase reporter assays. A reporter construct containing the putative GATA2 binding site was transfected into 3T3‐L1 preadipocytes, followed by CEL treatment with or without GATA2 knockdown. CEL treatment markedly enhanced the luciferase activity of the miR‐5099 promoter, while GATA2 knockdown attenuated this CEL‐stimulated activity (Figure 5I). Additionally, Chromatin Immunoprecipitation (ChIP) experiments confirmed that GATA2 binds to the putative GATA2 site within the miR‐5099 promoter, and this binding was enhanced by CEL treatment (Figure 5J). Together, these results indicate that GATA2, at least in part, modulates the promoter activity of miR‐5099 to mediate the effect of CEL on its expression. These findings demonstrate that adipocytes actively package CEL‐induced miR‐5099 into exosomes for systemic secretion, providing a mechanistic basis for the metabolic benefits in obese mouse models conferred by CEL‐EXOs derived from both pre‐ and mature adipocytes, as we observed.

To evaluate the delivery and functional potential of miR‐5099 via adipocyte‐derived exosomes, a Cy3‐labeled miR‐5099 mimic was synthesized and transfected into 3T3‐L1 preadipocytes, generating engineered exosomes (mir‐EXOs) containing the labeled miRNA. These mir‐EXOs were then administered to BMDMs. Flow cytometry and gene expression analysis confirmed the successful uptake of the labeled miR‐5099 by BMDMs and its delivery via the adipocyte‐derived exosomes (Figure S11A,B). This result demonstrates the efficacy of using adipocyte exosomes as carriers for miR‐5099. Importantly, it confirms that miR‐5099, when packaged and delivered in this physiologically relevant manner through exosomes naturally produced by adipocytes, retains its capacity to function as a physiological mediator within recipient BMDMs under natural conditions. Upon direct transfection of the miR‐5099 mimic into LPS/IFNγ‐induced BMDM M1 macrophages, a remarkable suppression of the M1 marker iNOS levels was observed (Figure 5K). The successful transfection of the miR‐5099 mimic into the macrophage cell line RAW264.7 was validated through immunofluorescence (Figure S11C). Notably, this direct administration of the miR‐5099 mimic significantly inhibited LPS/IFNγ‐induced M1 macrophage polarization, as demonstrated by the expression of M1 proinflammatory genes and measurements of ROS production (Figure 5L; Figure S11D–F).

Following ectopic miR‐5099 overexpression using an EGFP‐labeled vector in RAW264.7 macrophages, successful transfection was confirmed by GFP imaging, flow cytometry, and miR‐5099 quantification (Figure S11G,H). This miR‐5099 overexpression significantly suppressed the expression of LPS/IFNγ‐induced M1 polarization marker genes, while flow cytometry and IF staining revealed decreased populations of iNOS^+^ and CD11c^+^ cells (Figure S11I–K; Figure 5M). Concurrently, miR‐5099 overexpression attenuated intracellular ROS accumulation in activated M1 macrophages as measured by DCF fluorescence (Figure S11L). This anti‐inflammatory phenotype was consistently recapitulated in human THP‐1 M1 macrophages (Figure S11M–O).

### 2.6 Adipocyte‐Derived Exosomal miR‐5099 Modulates M1 Macrophage Polarization via Targeting c‐Met

**FIGURE 6:**
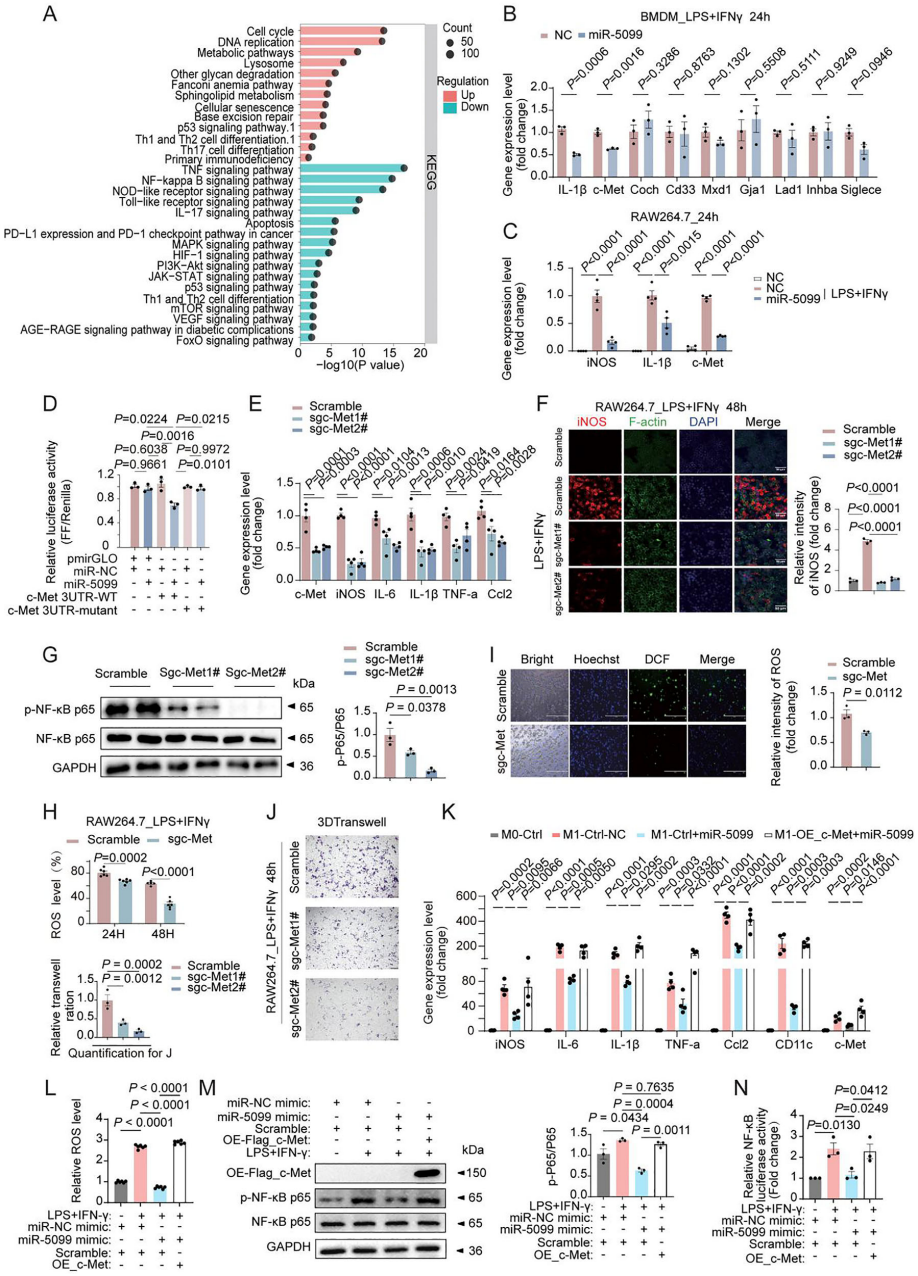
c‐Met mediate miR‐5099 targets c‐Met to regulate M1 macrophage activation and function. (A) KEGG pathway analyses of DEGs for the top 20 pathways. (B) RT‐PCR validation of 8 candidate target genes in BMDMs transfected with miR‐5099 mimic or NC (n = 3). (C) The expression changes of indicated genes at the RNA level in RAW264.7 cells stimulated without or with LPS/IFNγ (100 ng/mL; 20 ng/mL) transfected with miR‐5099 mimic or NC (n = 4). (D) Dual‐luciferase assay validating c‐Met as a direct target of miR‐5099 in HEK293T cells (n = 3). (E) The expression changes of M1 macrophage markers in LPS/IFNγ (100 ng/mL; 20 ng/mL) stimulated RAW264.7 M1 macrophages with or without c‐Met gene knockout (n = 4). (F) Representative IF staining for iNOS, F‐actin, and DAPI in RAW264.7 cells treated with or without LPS/IFNγ (100 ng/mL; 20 ng/mL) for 24 or 48 h, in the combination of c‐Met knockout (sgMET) (n = 3). Scale bar, 50 µm. (G) Western blot of p‐P65 and total P65 in RAW264.7 M1 macrophages with or without c‐Met gene knockout (n = 3). (H‐I) ROS level analysis in 48 h LPS/IFNγ (100 ng/mL; 20 ng/mL) stimulated RAW264.7 M1 macrophages with or without c‐Met gene knockout (H) (n = 6) and DCF staining and quantification (I) (n = 3). Scale bar, 200 µm. (J) Representative images of migration assays using 3D transwell and its quantification in LPS/IFNγ (100 ng/mL; 20 ng/mL) stimulated RAW264.7 M1 macrophages with or without c‐Met gene knockout (n = 3). Scale bar, 200 µm. (K–N) The expression analysis of inflammatory M1 macrophage marker genes (K), DCF fluorescent signaling for ROS levels (L), Western blot of p‐P65 and total P65 (M), NF‐κB luciferase reporter assay (N) in LPS/IFN‐γ (100 ng/mL; 20 ng/mL) stimulated RAW264.7 macrophages treated with or without miR‐5099 mimic (50 nm, 24 h), in combination with or without c‐Met overexpression (n  =  3). Comparison between the two groups was evaluated using an unpaired two‐tailed *t*‐test. One‐way ANOVA with Dunnet multiple comparison post hoc tests for E, F, G, J, and Tukey's post‐hoc test for the rest were used for comparing more than two groups. Data represent mean ± SEM.

To unravel the mechanisms underlying miR‐5099's regulation of M1 polarization, we treated BMDMs with miR‐5099 in the presence of LPS/IFNγ and identified differentially expressed genes (DEGs) (Figure S12A). Notably, GSEA analysis, KEGG and GO analysis identified that inflammation related pathways, such as TNF and NF‐κB pathways, are enriched in downregulated DEGs (Figure 6A; Figure S12B,C). By utilizing TargetScan and miRPathDB, we predicted miR‐5099‐regulated genes and pinpointed 8 candidate DEGs (Figure S12D; Figure 6B). Notably, among these candidates, c‐Met gene expression showed the most pronounced upregulation during LPS/IFNγ‐induced M1 polarization in RAW264.7 and BMDM cells (Figure S12E), while miR‐5099 treatment significantly downregulated c‐Met expression, alongside with M1 markers IL‐1β and iNOS (Figure 6C). To validate whether c‐Met is a direct target of miR‐5099, we employed a luciferase reporter assay. Mutation of the predicted miR‐5099 binding motif within the 3’ UTR of the c‐Met gene effectively abolished the miR‐5099‐mediated repression of c‐Met luciferase reporter expression (Figure 6D). These findings indicate that miR‐5099 downregulates c‐Met gene expression by directly binding to a specific motif in its 3' UTR, potentially causing c‐Met mRNA degradation, as evidenced by reduced c‐Met mRNA levels. Importantly, the knockout of c‐Met in RAW264.7 macrophages led to a decrease in LPS/IFNγ‐induced M1 polarization, evident from the reduced iNOS IF staining and the expression levels of M1 macrophage marker genes (Figure 6E,F; Figure S12F). In obese adipose tissue, the NF‐κB pathway serves as a crucial regulator of inflammation. It fosters M1 macrophage differentiation, triggering chronic, low‐grade inflammation that promotes insulin resistance and the onset of obesity‐related metabolic disorders [27]. Our findings indicate that c‐Met knockout markedly reduced p65 activity (Figure 6G), the pivotal protein subunit of the NF‐κB complex responsible for regulating the expression of pro‐inflammatory cytokines like TNF‐α, IL‐1β, IL‐6, and Ccl2. While evidence linking the non‐canonical NF‐κB proteins p100 and p52 to adipose tissue inflammation remains limited, our study found that c‐Met knockout downregulated both proteins, suggesting their potential involvement in obesity‐related inflammation (Figure S12G,H). Furthermore, c‐Met knockout reduced ROS production in M1‐polarized macrophages and attenuated their migratory capacity (Figure 6H–J; Figure S12I). The deletion of c‐Met in LPS/IFN‐γ‐induced M1 BMDM resulted in a notable decrease in the F4/80+/CD11c^+^ cell population (Figure S12J,K). In both c‐Met knockout and miR‐5099‐treated groups, LPS/IFN‐γ‐induced M1 RAW264.7 macrophages showed similar suppression of inflammatory gene expression, reduction in the F4/80^+^/CD11c^+^ cell population, and decreased ROS levels, and c‐Met knockout did not enhance the anti‐inflammatory effects of miR‐5099 treatment (Figure S12L–N). Notably, miR‐5099‐mediated inhibition of M1 macrophage polarization and inflammation in RAW264.7 cells was reversed by c‐Met overexpression, which restored the downregulated inflammatory gene expression and reduced ROS levels (Figure 6K,L). Furthermore, c‐Met overexpression blocked the suppressive effect of miR‐5099 on NF‐κB signaling, as shown by increased p65 activity and NF‐κB luciferase reporter assay results (Figure 6M,N). These findings suggest that c‐Met functions downstream of miR‐5099 as a negative mediator, promoting M1 polarization and inflammation via the NF‐κB pathway. Collectively, these data demonstrate that adipocyte‐derived and CEL‐upregulated exosomal miR‐5099 acts via extracellular delivery to macrophages, where it suppresses c‐Met expression and NF‐κB signaling. This miR‐5099/c‐Met/NF‐κB axis mediates adipocyte‐macrophage communication, ultimately regulating macrophage M1 polarization, proinflammatory responses, and potentially, tissue infiltration of macrophages.

### 2.7 miR‐5099 is Essential for the Effects of CEL Treated Adipocyte‐Derived Exosomes on Anti‐Inflammation and the Improvement of Metabolic Health in Obese Mice

**FIGURE 7:**
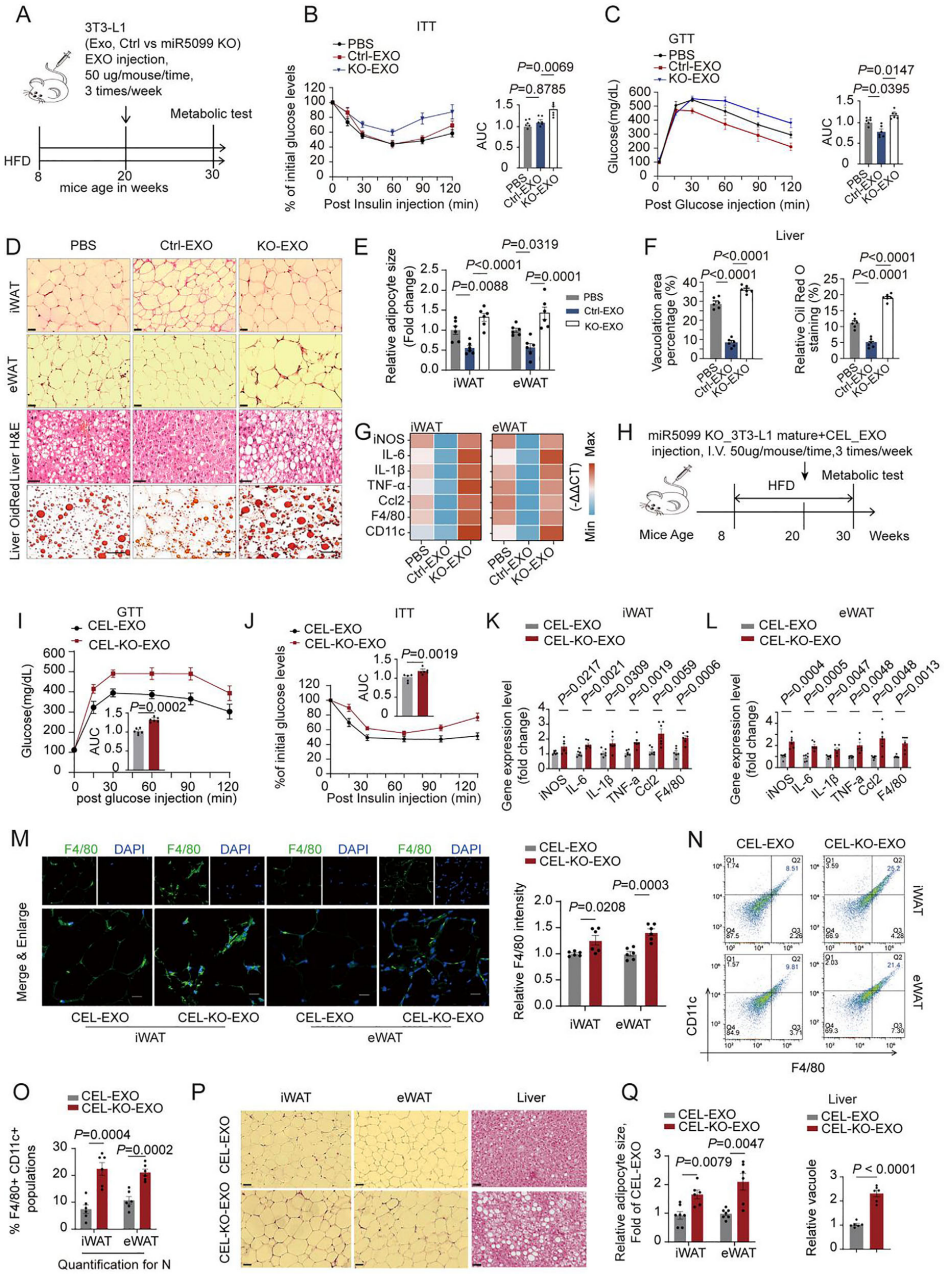
miR‐5099 is essential to the impact of adipocyte‐derived EXOs on suppressing M1 macrophage polarization and adipose tissue inflammation. (A) Schematic illustrating the approach to generate experimental obese mice. Male C57Bl/6J mice (8 weeks) were fed an HFD for 12 weeks, followed by the administration of vehicle (PBS), adipocyte‐derived exosomes without (Ctrl‐EXOs) or with miR‐5099 knockout (KO‐EXOs) derived from 3T3‐L1 preadipocytes (I.V. injections, 50 µg/mouse, 3times/week) for an additional 10 weeks. These mice were used for experiments in Figure 7B–J. (B–C) GTT (B) and ITT (C) and associated AUC in HFD induced obese mice (n = 6). (D) Representative H&E images of iWAT and eWAT (Scale bar, 50 µm), and liver(Scale bar, 50 µm), and Oil Red O staining of liver sections (scale bar, 20 µm). (E) Quantification of adipocyte size in iWAT and eWAT. (F) Quantification of liver microvesicular vacuoles and Oil Red O staining for liver lipid droplets using ImageJ. (G) The expression changes of M1 macrophage markers at the RNA level in iWAT and eWAT of EXO‐treated mice (n = 6). (H) Schematic illustrating the approach to generate experimental mice. Male C57Bl/6J mice (8 weeks) were fed an HFD for 12 weeks, followed by the administration of 3T3‐L1 adipocyte‐derived exosomes with CEL treatment (0.4 µm, 48 h) without (CEL‐EXOs) or with miR‐5099 knockout (CEL‐KO‐EXOs) (I.V. injections, 50 µg/mouse, 3times/week) for an additional 10 weeks. These mice were used for experiments in Figure 7I–Q. (I‐J) GTT (I) and ITT (J) and associated AUC in obese mice with indicated treatment (n = 5–6). (K‐L) The expression analysis of M1 macrophage markers in iWAT (K) and eWAT (L) of the indicated obese mice (n = 6). (M) Representative IF staining for F4/80 and DAPI in iWAT and eWAT (n = 6). Scale bar, 20 µm. (N‐O) Flow cytometric plot (N) and quantification (O) of CD11c^+^/F4/80^+^ macrophage population in iWAT and eWAT (n = 6). (P‐Q) Representative H&E images of iWAT, eWAT, and liver (P), and quantification for adipocyte size of both iWAT and eWAT and liver microvesicular vacuoles (Q) (n = 6). Scale bar, 50 µm. Comparison between two groups was evaluated using an unpaired two‐tailed *t*‐test. One‐way ANOVA with Tukey's post‐hoc test was used for comparing more than two groups. Data represent mean ± SEM.

We explored whether miR‐5099 is essential for mediating the function of adipocyte‐derived exosomes in enhancing the metabolic health of established obese mice. To investigate this, we isolated KO‐EXOs (exosomes derived from 3T3‐L1 preadipocytes with miR‐5099 knockout (KO) (Figure 7A; Figure S13A). Interestingly, we found that the depletion of miR‐5099 did not alter the body weight reduction effect induced by Ctrl‐EXOs alone, and there was no significant change in food intake (Figure S13B–D). Nevertheless, treatment with KO‐EXOs reversed the reduction in fat mass of iWAT and eWAT caused by Ctrl‐EXO treatment, while concurrently leading to a decrease in lean mass in obese animals (Figure S13E–I). KO‐EXO treatment exacerbated glucose intolerance and insulin resistance (Figure 7B,C). Critically, KO‐EXOs with depleted miR‐5099 completely abolished the beneficial effects of preadipocyte‐derived Ctrl‐EXOs. These effects included reduced adipocyte hypertrophy and alleviated liver steatosis (Figure 7D–F). Ctrl‐EXOs suppressed inflammatory phenotypes, including M1 macrophage infiltration (Figure S13J,K) and marker gene expression (Figure 7G; Figure S13L). F4/80 and iNOS staining were similarly reduced (Figure S13M). However, KO‐EXO treatment completely reversed these anti‐inflammatory outcomes. Collectively, these findings demonstrate that miR‐5099 is essential for the therapeutic benefits of preadipocyte‐derived exosomes to mitigate key metabolic and inflammatory hallmarks of obesity.

Furthermore, we examined whether exosomal miR‐5099 also mediates the effects of CEL‐EXOs in improving the metabolic health of obese animals. To determine if exosomal miR‐5099 mediates the metabolic benefits of CEL‐treated adipocyte exosomes (CEL‐EXOs), we harvested exosomes from 3T3 adipocytes treated with CEL, either with intact miR‐5099 (CEL‐EXOs) or with miR‐5099 knockout (CEL‐KO‐EXOs). When administered directly to macrophages (BMDM and RAW264.7), CEL‐EXOs reduced M1 marker gene expression compared to control exosomes (Ctrl‐EXO), while CEL‐KO‐EXOs largely abrogated this anti‐inflammatory effect (Figure S14A,B). To evaluate whether miR‐5099 mediates the effects of CEL‐EXOs in vivo in obese animals, we administered CEL‐EXOs and CEL‐KO‐EXOs to established obese mice via intravenous injection (Figure 7H). Interestingly, we observed no significant differences in body weight or daily food intake between the CEL‐EXO and CEL‐KO‐EXO groups (Figure S14C–E). While overall fat mass was comparable, a slight increase in iWAT mass was noted in the CEL‐KO‐EXO group (Figure S14F–H). Additionally, CEL‐KO‐EXO treatment elevated fasting serum total cholesterol (TC) and triglycerides (TG), whereas glucose levels remained unchanged (Figure S14I–K). Furthermore, CEL‐KO‐EXO treatment led to a reduction in the respiratory activity of obese mice (Figure S14L). Critically, compared to CEL‐EXO treatment, administration of miR‐5099‐depleted exosomes (CEL‐KO‐EXOs) exacerbated metabolic dysfunction in obese mice, manifesting as increased glucose intolerance and insulin resistance (Figure 7I,J). Moreover, CEL‐KO‐EXOs reversed the anti‐inflammatory effects of CEL‐EXOs. Specifically, M1 macrophage marker genes were elevated (Figure 7K,L). We also observed enhanced F4/80^+^ staining (Figure 7M) and increased CD11c^+^F4/80^+^ macrophage infiltration by CEL‐KO‐EXOs (Figure 7N,O).

Histological analysis confirmed that CEL‐KO‐EXOs worsened obesity‐associated adipocyte hypertrophy and liver steatosis (Figure 7P,Q). These data suggest that miR‐5099 plays a vital role in mediating the positive effects of CEL‐induced, adipocyte‐derived exosomes on metabolic health. This miRNA facilitates crucial communication between adipocytes and macrophages within obese adipose tissue, suppressing the infiltration of proinflammatory macrophages in obese adipose tissue. Depletion of miR‐5099 disrupts this regulation, intensifying adipose tissue inflammation and worsening obesity related metabolic complications.

### 2.8 miR‐5099 is a Deliverable Bioactive Molecule Ameliorating Inflammation and Metabolic Complications Associated with Obesity

To evaluate the potential therapeutic effects of exosomes with elevated miR‐5099 on improving the metabolic health of obese animals, we administered EXO‐OE (exosomes derived from 3T3‐L1 preadipocytes with miR‐5099 ectopic expression) to established obese mice (Figure S15A). The overexpression of miR‐5099 in both cells and exosomes was confirmed through flow cytometry, immunofluorescence, and RT‐PCR analyses (Figure S15B–F). EXO‐OE treatment did not alter body weight, fat mass, or food intake compared to EXO‐Ctrl (Figure S15G–J). However, it notably improved glucose tolerance and insulin sensitivity (Figure S15K,L). With EXO‐OE treatment of obese mice, fasting serum glucose levels exhibited a decrease, while postprandial serum glucose levels remained unchanged (Figure S15M,N). Additionally, EXO‐OE treatment significantly suppressed M1 macrophage infiltration, shown by reduced F4/80 levels (Figure S15O,P), lower percentage of F4/80^+^CD11c^+^ macrophages (Figure S15Q), and decreased expression of M1 inflammatory genes in adipose tissues and liver compared to the EXO‐Ctrl group (Figure S15R). The increased levels of miR‐5099 in exosomes also alleviated obesity‐induced adipocyte hypertrophy and liver steatosis, as demonstrated by decreased adipocyte size and reduced lipid droplet accumulation in the liver (Figure S15S,T).

**FIGURE 8:**
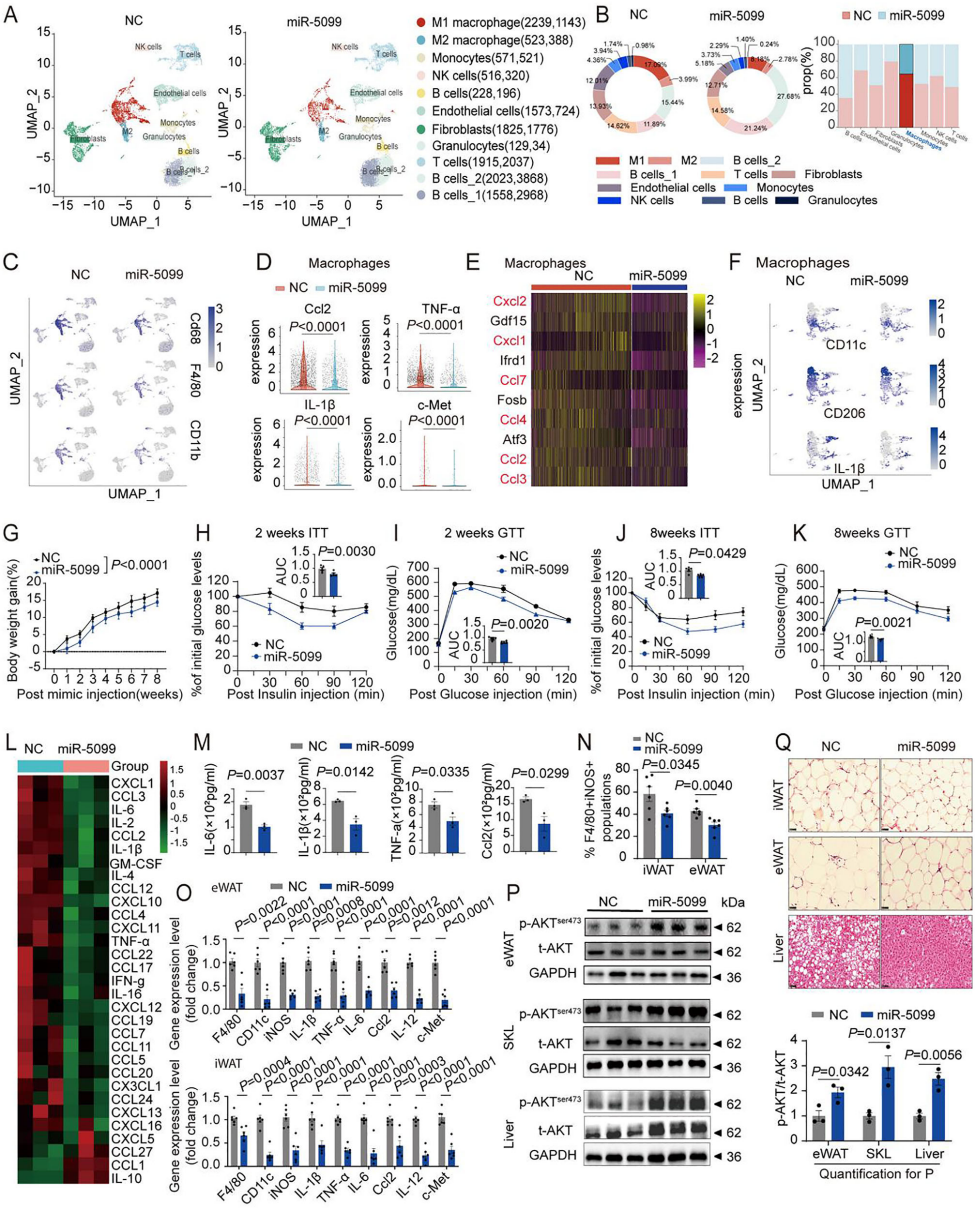
Administration of miR‐5099 suppresses inflammatory M1 macrophage accumulation, alleviates adipose tissue inflammation, and enhances the metabolic health of obese mice. Male C57Bl/6J mice (8 weeks) were fed an HFD for 4 weeks, followed by the administration of agomiR‐5099 (miR‐5099) or agomiR‐NC (NC) (I.V. injections, 2.5 nmol/mouse/time, 3 times/week) for an additional 8 weeks. These mice were used for experiments in Figure 8. (A) UMAP plot displaying single‐cell transcriptomic profiling of SVF cells from iWAT of obese mice with indicated treatment, delineating 31 clusters categorized into 11 major cell types. (B) Proportion of immune cells, including macrophage subtypes, among SVF cells in each experimental group. (C) Expression of macrophage markers (Adgre1, Itgam, Cd68) across all cell clusters. (D) Expression of proinflammatory factors and c‐Met gene in macrophages sub‐cluster of each experimental group. (E) Heatmap of the top 10 downregulated DEGs in macrophages based on single cell sequencing data. (F) Expression change of CD11c, CD206, and Il‐1β in macrophages. (G) Body weight changes over time of obese mice with indicated treatment (n  =  6). (H–I) GTT (H) and ITT (I) and associated AUC at 2 weeks post‐injection (n = 5‐6). (J–K) GTT (J) and ITT (K) and associated AUC at 8 weeks post‐injection (n = 6). (L) Heatmap of serum cytokine profiling of indicated obese mice by Luminex multiplex assay (n = 3). (M) Quantification of some key inflammatory cytokines (n = 3). (N) Flow cytometry quantification of CD11c^+^F4/80^+^ macrophage population in eWAT and iWAT of indicated obese mice (n = 6). (O) The expression level of inflammatory factors and c‐Met in eWAT (top) and iWAT (bottom) of the indicated obese mice (n = 6). (P) Western blot of p‐AKT(Ser^473^) and total AKT in eWAT, skeletal muscle, and liver of indicated obese mice (n = 3). (Q) Representative H&E images of iWAT, eWAT, and the liver of the indicated obese mice. Scale bar, 50 µm. Comparison between the two groups was evaluated using an unpaired two‐tailed *t*‐test. Data represent mean ± SEM.

Our data demonstrated the crucial role of miR‐5099 in mediating adipocyte‐macrophage communication, thereby targeting adipose tissue inflammation and enhancing metabolic health in obese animals. Therefore, we next evaluated the therapeutic potential of directly administering miR‐5099 to obese animals. We treated obese mice directly with the synthetic AgomiR‐5099 mimic(miR‐5099) and control mimic (NC) (Figure S16A) and conducted single‐cell analysis of the SVF isolated from the adipose tissue of the miR‐5099 and NC treated obese mice (Figure S16B). At resolution 1.0 with 30 dimensions, the cells were classified into 31 clusters, which were further consolidated into 11 distinct cell populations representing various types of infiltrated cells in adipose tissue (Figure 8A). Among these 31 cell clusters, 6 exhibited elevated expression levels of macrophage markers such as Adgre1, Itgam, and Cd68 (Figure S16C,D). MiR‐5099 treatment reduced total ATM infiltration (21.08%–10.96%), particularly the M1 subpopulation (17.09%–8.18%) (Figure 8A,B). Consistently, the expression of macrophage marker genes Adgre1 (F4/80), Itgam (CD11b), and Cd68 was downregulated (Figure 8C). Moreover, inflammatory genes such as Ccl2, TNF‐α, and IL‐1β were significantly downregulated by miR‐5099 treatment in macrophage populations (Figure 8D). The miR‐5099 target gene in ATMs, c‐Met, was notably downregulated by miR‐5099 treatment (Figure 8D). Notably, among the top 10 downregulated DEGs in macrophages, many inflammatory chemokines are encompassed, including Cxcl2, Cxcl1, Ccl7, Ccl4, Ccl3, and Ccl2, etc. (Figure 8E,F). Gene set enrichment analysis (GSEA), GO analysis, and KEGG analysis of the DEGs further highlighted a global repression of inflammatory response and its related pathways in miR‐5099 treated obese AT (Figure S16E–G). Owing to the limitation of M1‐M2 dichotomy (M1:CD11cHi; M2:CD206Hi) [28, 29, 30, 31], we further divided ATM (F480+/CD68+/CD11b+) into 7 subclusters to adequately address the diversity (Figure S16H,I). Again, most macrophage subtypes exhibited reduced infiltration in adipose tissue following miR‐5099 treatment. The CD206+ CD11c+ macrophage population, which represents the predominant macrophage subset infiltrating the adipose tissue, displayed a significant, nearly 50% reduction in infiltration. Additionally, the CD206‐CD11c‐IL‐1β+ macrophage subset exhibited a decrease to merely one‐third of its initial presence in adipose tissue after miR‐5099 treatment. (Figure S16I). Expression of CD11c, CD206, and IL‐1β decreased in all macrophages following miR‐5099 treatment (Figure 8F). The expression of the target gene c‐Met also dropped in three of seven subtypes (Figure S16J). Notably, among the DEGs among the top 4 major macrophage groups, a common reduction in pro‐inflammatory chemokines was observed, including Cxcl2, Cxcl1, and others (Figure S16K). Collectively, direct administration of miR‐5099 in obese mice showed strong anti‐inflammatory effects, especially targeting AT inflammation, highlighting its therapeutic potential to improve the adipose tissue microenvironment.

Notably, miR‐5099 reduced body weight without affecting food intake (Figure 8G; Figure S17A,B). Since EXO‐OE failed to reduce weight, direct miR‐5099 administration appears more potent. Treatment of miR‐5099 decreased iWAT and liver weights, lowering the fat‐to‐body weight ratio (Figure S17C,D). Serum cholesterol and triglycerides also dropped, while metabolic rates increased (Figure S17E–I). Furthermore, glucose tolerance and insulin sensitivity improved significantly from weeks 2 to 8 (Figure 8H–K). Cytokine profiling analysis using Luminex multiplex assay in obese animals revealed that miR‐5099 treatment significantly decreased inflammatory cytokines in serum on a large scale. This reduction was further confirmed through ELISA assays, validating the decrease in key cytokines such as IL‐1β, IL‐6, TNF‐α, and Ccl2 (Figure 8L,M). Consistent with these systemic effects, miR‐5099 administration markedly attenuated ATM accumulation in obese AT compared to NC controls. This reduction in inflammatory ATM infiltration was demonstrated by a decreased percentage of inflammatory macrophages (Figure 8N; Figure S17J), diminished staining of M1 macrophage F4/80 and iNOS markers (Figure S17K), and downregulated expression of pro‐inflammatory genes within adipose tissue (Figure 8O). The direct anti‐inflammatory effects of miR‐5099 treatment in obese mice underline the observed improvements in systemic metabolic health, including enhanced glucose tolerance and insulin sensitivity. This improved insulin signaling was mechanistically supported by elevated AKT phosphorylation levels in key metabolic tissues such as eWAT, liver, and skeletal muscle (Figure 8P). Furthermore, histological analyses revealed significant amelioration of adipose tissue pathology, characterized by reduced adipocyte hypertrophy (decreased adipocyte size; Figure 8Q; Figure S17L) and substantial attenuation of obesity‐associated hepatic steatosis (Figure 8Q).

Our in vitro analysis further demonstrated that miR‐5099 overexpression or direct treatment with synthetic miR‐5099 mimic significantly augmented insulin‐stimulated glucose uptake in both adipocytes (3T3‐L1) and myocytes (C2C12) (Figure S17M–O). In hepatocytes, miR‐5099 suppressed glucagon‐stimulated glucose production in HepG2 cells and ameliorated lipid accumulation in PA/OA‐exposed Huh7 cells (Figure S17P,Q). Furthermore, miR‐5099 consistently enhanced insulin‐stimulated AKT phosphorylation across these cell types, confirming improved insulin signaling (Figure S17R). These findings collectively underscore the robust, cell‐autonomous metabolic benefits of miR‐5099 across key insulin‐responsive tissues. By simultaneously enhancing insulin sensitivity, reducing ectopic lipid deposition, and improving hepatic glucose handling, miR‐5099 targets fundamental drivers of metabolic dysfunction. The broad multi‐tissue effectiveness and robust anti‐inflammatory properties of miR‐5099 highlight its potential as a promising therapeutic candidate for intricate metabolic disorders such as obesity, type 2 diabetes, and potentially non‐alcoholic fatty liver disease (NAFLD/NASH), addressing core pathogenic factors like insulin resistance, inflammation, and lipid dysregulation.

## 3 Discussion

Our study identifies a novel microRNA, miR‐5099, which is expressed and upregulated by CEL in adipocytes, being packaged into adipocyte‐derived exosomes. This exosomal miR‐5099 orchestrates adipocyte‐macrophage communication, effectively suppressing pro‐inflammatory M1 macrophage polarization both in vitro and in vivo within obese adipose tissue. Concomitantly, it enhances systemic insulin sensitivity across metabolic tissues. By simultaneously targeting AT inflammation, remodeling the AT microenvironment, and potentiating insulin responsiveness, miR‐5099 ameliorates obesity and its associated metabolic complications. These findings establish exosomal miR‐5099 as a promising therapeutic strategy for combating obesity‐related inflammation and its downstream metabolic disorders.

CEL exhibits broad immunomodulatory effects, treating conditions ranging from autoimmune to metabolic disorders [17, 18, 19, 20]. However, significant cytotoxicity and complex pleiotropic effects hinder clinical use. Therefore, strategies to maintain efficacy while reducing toxicity are essential. Our research reveals that CEL functions through an indirect mechanism. Specifically, it exerts anti‐inflammatory effects by altering the miRNA profile of adipocyte‐secreted exosomes. Substantial evidence indicates that exosomes function as crucial signaling mediators for intercellular communication and interorgan crosstalk by delivering bioactive cargo to target cells or interacting directly with cell surface receptors, thereby coordinating physiological and pathological processes [32, 33]. For instance, adipose‐secreted exosomes coordinate communication both within adipose tissue (between adipocytes and other cell types) and between adipose tissue and other metabolic tissues. This coordination is essential for regulating the local adipose tissue microenvironment and function, ultimately impacting systemic metabolic health [12, 34]. However, unlike the consistent anti‐inflammatory effects of CEL‐treated adipocyte‐derived exosomes (CEL‐EXO) observed both in vitro and in vivo, exosomes derived from untreated adipocytes (Ctrl‐EXOs) did not uniformly reduce inflammatory responses. This inconsistency suggests that Ctrl‐EXOs are not biologically inert, shedding light on a potential confounding factor when using naïve exosomes as a reliable therapeutic benchmark. We propose that CEL actively reshapes the exosome's contents, thereby converting exosomes into a cohesive therapeutic signal. This distinction emphasizes that the specific composition of the native exosomal cargo, rather than just the vesicle itself, serves as the pivotal determinant of its bioactivity, underscoring the precise therapeutic potential of modulating this intrinsic delivery system beyond its function acting as a delivery system and biomarker.

The signaling capacity of exosomes is largely determined by their microRNA (miRNA) content. Adipose tissues are key suppliers of circulating exosomal miRNAs, which act as gene regulators in distant tissues, thereby functioning as novel adipokines [12]. Notably, the miRNA profile of exosomes secreted by adipocytes from obese human visceral adipose tissue differs markedly from that of lean individuals. Furthermore, alterations in adipocyte‐derived exosomal miRNAs have been linked to influences on metabolic health [35, 36]. Our study demonstrates that exosomes from both CEL‐treated and untreated 3T3‐L1 preadipocytes reduce M1 macrophage accumulation in obese adipose tissue. Notably, exosomes from matured 3T3‐L1 adipocytes without CEL treatment do not show this beneficial effect on M1 macrophage accumulation. This differential effect may stem from altered levels of miR‐5099, a novel immunosuppressive adipocyte‐derived exosomal miRNA identified in our study. Indeed, our results demonstrate that elevated miR‐5099 levels within preadipocytes and CEL‐treated adipocyte exosomes are accountable for the observed anti‐inflammatory effects both in vivo and in vitro.

The functional role of miR‐5099 within adipose tissue has yet to be investigated. Existing knowledge regarding miR‐5099's biological functions is very limited, with past studies primarily emphasizing its tissue‐specific alteration in pathological contexts and its potential utility as a biomarker. Specifically, miR‐5099 levels were found to be significantly decreased in the kidneys of HFD induced obese mice and in the joint tissue of old mice [37, 38]. Conversely, elevated levels of miR‐5099 have been demonstrated in cases of ischemic stroke, injured liver, and podocyte injury induced by puromycin aminonucleoside [39, 40, 41]. We observed decreased miR‐5099 levels in the adipose tissue, serum, and exosomes of obese mice. Conversely, CEL treatment increased miR‐5099 expression in adipose tissue. Mechanistically, CEL likely modulates this expression by regulating GATA2 binding to the miR‐5099 promoter. The mechanisms through which obesity downregulates miR‐5099 and whether miR‐5099 can regulate other adipose functions, still remain unknown. Our study is the first to demonstrate that exosomal miR‐5099 from CEL‐treated adipocytes plays a significant role in regulating adipose tissue inflammation and enhancing the metabolic health of obese mice by mediating communication between adipocytes and macrophages. This mechanism elucidates how CEL reduces inflammation and enhances metabolic health in obese animals treated with CEL‐derived adipocyte exosomes.

Of note, adipocytes from obese mice release more exosomes than those from lean mice [42]. Given this increased secretion, it is reasonable to observe that numerous adipose tissue‐derived miRNAs identified so far are linked to the progression of metabolic dysfunction. For example, exosomal miR‐34a from adipocytes inhibits M2 macrophage polarization, exacerbating obesity‐induced adipose inflammation [15]. MiR‐802 promotes lipogenesis and shifts macrophages toward the M1 phenotype [16]. Additionally, miR‐155, a known proinflammatory miRNA, is notably upregulated in microvesicles secreted by adipose tissue from obese mice compared to lean mice [43]. Present in exosomes from both adipocytes and adipose tissue macrophages (ATM) [13, 44], miR‐155 triggers chemokine release, promotes proinflammatory M1 macrophage polarization, and contributes to systemic inflammation [13, 45]. It also hampers thermogenesis, with its inhibition enhancing brown adipocyte differentiation and promoting “browning” of white adipose tissue [46]. MiR‐155 deficiency enhances insulin sensitivity and glucose tolerance, highlighting its role in metabolic syndrome [13]. However, some miRNAs have still been found to exert beneficial regulatory effects on metabolic complications. ATM‐derived miR‐146a suppresses inflammation, countering diet‐induced obesity [47], while miR‐223 hampers pro‐inflammatory M1 activation and boosts anti‐inflammatory M2 responses, safeguarding against systemic insulin resistance [48].

Notably, our newly identified miR‐5099 also combats metabolic complications through a dual mechanism of action. Our study reveals that miR‐5099 functions as both an anti‐inflammatory agent and a potent, multi‐tissue insulin sensitizer. We demonstrated that miR‐5099 directly enhances insulin signaling in adipocytes, myocytes, and hepatocytes. These findings establish miR‐5099 as a novel insulin‐sensitizing miRNA that acts directly on the insulin pathway, rather than merely as an anti‐inflammatory mediator. The dual action positions miR‐5099 as a promising therapeutic candidate for metabolic syndrome. While miR‐5099 holds significant therapeutic promise, its clinical translation faces key challenges. A primary concern is off‐target effects; like other miRNAs, it may unintentionally regulate multiple transcripts. Enhancing specificity through chemical modifications will be essential. Another hurdle is biodistribution, systemically delivered exosomes naturally accumulate in the liver and spleen, which aligns with hepatocyte targeting but limits reach to adipose tissue and skeletal muscle. Engineering exosomes with tissue‐specific targeting ligands could overcome this barrier. Although adipocyte‐derived exosomes are biocompatible, future work must ensure their rigorous purification and humanized sourcing to minimize immunogenicity. Addressing these limitations is critical to advancing miR‐5099 toward a viable metabolic therapy.

MicroRNAs usually control gene expression in target cells, altering cell characteristics. Our study reveals that miR‐5099 modulates M1 macrophage polarization by targeting the c‐Met/NF‐κB axis to suppress M1 polarization and mitigates inflammation within obese adipose tissue. The tyrosine kinase c‐Met, the receptor of hepatocyte Growth Factor (HGF), constitutes a pleiotropic signaling axis with critical roles in metabolism, inflammation, and tissue homeostasis. Activation of c‐Met has been associated with promoting lipid accumulation in adipocytes [49] and plays a pivotal role in β‐cell equilibrium, regulating glucose metabolism in insulin‐responsive cells such as adipocytes, β‐cells, hepatocytes, and muscle cells [50, 51, 52, 53, 54]. Beyond metabolism, HGF‐Met signaling is a potent immunomodulator and participates broadly in modulating inflammatory responses. c‐Met is notably present in macrophages and activated monocytes, especially in conditions resembling inflammation, influencing macrophage reactions and migration [55, 56, 57]. However, the functional outcome of HGF‐Met signaling is context‐dependent. While it can promote tissue repair through M2 polarization [58], it paradoxically contributes to exacerbated inflammation and fibrosis in other settings (e.g., lung injury), where it enhances uncontrolled recruitment and activation of immune cells toward a pro‐inflammatory and profibrotic phenotype [59]. Thus, the HGF‐Met pathway serves as a complex regulator connecting metabolic dysregulation (such as obesity and IR) with inflammation and tissue remodeling, which provides crucial insights into the pathophysiology of metabolic disorders and related conditions. Here, we present the novel finding that miR‐5099, originating from adipocytes, downregulates the expression of c‐Met in infiltrated macrophages within adipose tissue. This regulation occurs through the binding of miR‐5099 to the 3’UTR region of the c‐Met mRNA, potentially triggering the degradation of c‐Met mRNA, as evidenced by the observed reduction in c‐Met mRNA levels. The miR‐5099‐mediated downregulation of c‐Met in macrophages is crucial. Our data show that deleting c‐Met suppresses the NF‐κB pathway and its regulatory targets, inflammatory factors. This action may ultimately hinder pro‐inflammatory M1 polarization and migration, thereby alleviating adipose tissue inflammation in obese animals. There is a strong association between elevated HGF levels and conditions like obesity and insulin resistance [49, 60]. Weight loss has been shown to reduce HGF levels. Investigating the impact of miR‐5099 administration on local or systemic HGF levels and the HGF‐Met signaling pathway would be enlightening. Such investigations could unveil innovative approaches for managing metabolic inflammation associated with obesity.

## 4 Methods and Materials

### 4.1 Animal Studies

Wild‐type (WT) male C57BL/6 mice were obtained from the Animal Facility of the Faculty of Health Sciences at the University of Macau. These mice were maintained under a 12‐h light/dark cycle with unlimited access to water and food. To induce obesity, the mice were provided with a high‐fat diet (HFD) comprising 60 kcal% fat (D12491, Research Diets Inc., New Brunswick, NJ) starting at 8 weeks of age for the specified duration. Mice fed a normal chow diet (NCD) with 10 kcal% fat were used as controls, with food available Ad libitum. All animal experiments were conducted in accordance with the University of Macau Research Guidelines for the Care and Use of Laboratory Animals (Animal ethics approval number: UMARE‐012‐2020). Animals were randomly assigned to experimental groups, and all procedures were approved by the University of Macau Animal Ethics Committee. The vehicle control groups received corresponding volumes of a diluent solution containing 0.4% or 2% DMSO, respectively. Daily intraperitoneal (i.p.) injections were administered 4 h before the onset of the dark cycle. Body weight and food intake were recorded daily just before drug administration. Body composition was assessed at the beginning and end of the study using the EchoMRI 3‐in‐1 (Echo Medical Systems). At the end of the treatment period, mice were humanely euthanized, and tissues were collected, snap‐frozen in liquid nitrogen, and stored at –80°C for subsequent biochemical analyses.

### 4.2 Glucose Tolerance and Insulin Tolerance Tests

Insulin tolerance tests (ITT) and glucose tolerance tests (GTT) were conducted as previously described [5]. For the GTT, mice received a single dose of glucose (2 g/kg body weight) via intraperitoneal (i.p.) injection following a 16‐h fasting period. In the case of ITT, mice underwent a 6‐h fast before being i.p. injected with insulin (0.7 units/kg body weight for HFD mice). Blood samples were obtained from the tail vein at specific time points to measure glucose levels. Glucose levels were assessed using a glucometer with tail vein blood samples collected at designated time intervals.

### 4.3 Whole‐Body Metabolism Analysis

Oxygen consumption (VO_2_), carbon dioxide production (VCO_2_), energy expenditure, and locomotor activity were monitored using the Oxymax Lab Animal Monitoring System (Columbus Instruments). Animals had ad libitum access to food and water. The system and gas analyzers were calibrated as per guidelines. Mice were acclimatized for 12 h in sealed chambers before data collection. Measurements were taken continuously over 72 h.

### 4.4 Histology and Immunofluorescence Staining

Tissues were harvested from mice under terminal anesthesia and fixed in 4% paraformaldehyde (PFA) overnight at 4°C. Adipose tissue and liver samples were embedded in paraffin and sectioned at 4 µm thickness for F4/80 and Hematoxylin and Eosin (H&E) staining. Liver tissues were also embedded in optimal cutting temperature (OCT) compound for Oil Red O staining at 8 µm thickness. For immunofluorescence analysis, F4/80 was labeled with FITC using a F4/80 monoclonal antibody (eBioscience, 14‐4801‐821,1:100), F‐actin cytoskeleton was stained with Alexa Fluor 488‐conjugated phalloidin (Invitrogen, A12379, 1:400), and nuclei counterstained with DAPI (Invitrogen, p36966) or Hoechst (Invitrogen, H3570) per manufacturers’ instructions. For iNOS detection, cells were incubated with Alexa Fluor 594‐conjugated anti‐Nos2 antibody (BioLegend, 696804, 1:1000). Fluorescence signals were detected using an Image Stream Mark II flow cytometer (Amnis) or Carl Zeiss LSM710 or LSM880 confocal microscope.

### 4.5 Serum TC, TG, and Cytokine Measurement

Serum levels of total triglycerides (TG) and total cholesterol (TC) were quantified using commercial assay kits (Triglyceride, Solarbio, BC0625‐100T/96S; Total Cholesterol: Solarbio, BC1985‐100T/96S) following the provided instructions. For cytokine analysis, post‐serum collection, cytokines were screened comprehensively by Wayen Biotechnologies (Shanghai) using the Bio‐Plex Pro Mouse Chemokine Panel 31‐Plex (#12009159) on a Luminex 200 system (Luminex Corporation, Austin, TX, USA).

### 4.6 Measurement of Serum AST, ALT, BUN, and LDH

Blood samples were collected and centrifuged at 3000 rpm for 10 min at 4°C to isolate serum. The activities of aspartate aminotransferase (AST) and alanine aminotransferase (ALT), Lactate dehydrogenase (LDH), along with the levels of blood urea nitrogen (BUN), were assessed using commercial assay kits (Nanjing Jiancheng Bioengineering Institute, Nanjing, China) following the provided instructions.

### 4.7 Bone Marrow Derived Cell Isolation and Culture

Bone marrow‐derived macrophages (BMDM) were isolated and cultured as described [61]. Briefly, 6–8‐week‐old female WT C57BL/6J mice were euthanized with 75% ethanol for 3–5 min to sterilize. The femurs were gently extracted, washed in Dulbecco's Phosphate Buffered Saline (DPBS) with 1% penicillin/streptomycin (P/S) five times, and then the bone marrow was flushed out and collected in RPMI‐1640 medium supplemented with heat‐inactivated Fetal Bovine Serum (FBS) at 56°C for 30 min. The marrow was passed through a 29G needle multiple times, filtered through a 40 µm cell strainer, and centrifuged at 1000 rpm for 6 min. The resulting cell pellet was treated with red blood cell lysis buffer, incubated, and then resuspended in FBS‐containing 1640 medium. After centrifugation, the cells were cultured in RPMI‐1640 medium with Macrophage Colony Stimulating Factor (M‐CSF), P/S, and 10% FBS, seeded in 12‐well plates, and incubated at 37°C in a 5% CO2 atmosphere. After 4–5 days, the culture medium was changed. On days 7–9, cells were polarized into M2 phenotype with interleukin 4 (IL‐4; 20 ng/mL) or into M1 phenotype with lipopolysaccharide (LPS; 10 ng/mL) and interferon γ (IFN‐γ; 20 ng/mL). In some experiments, CEL or exosomes were added at various concentrations along with the stimulating agents. After 24 h, cells were harvested for RNA isolation for subsequent experiments.

### 4.8 RAW264.7 and THP‐1 Cell Culture

RAW264.7 murine macrophage cells were obtained from ATCC and cultured in DMEM supplemented with 10% FBS and 1% penicillin‐streptomycin at 37°C in a humidified 5% CO_2_ atmosphere. To induce M1 polarization, cells were exposed to 100 ng/mL LPS and 20 ng/mL recombinant mouse IFN‐γ for 24 h. In specific experiments, CEL or exosomes were also used for cell treatment. The human monocytic cell line THP‐1 was purchased from the ATCC and cultured in RPMI‐1640 medium supplemented with 10% FBS and 1% penicillin‐streptomycin at 37°C in a 5% CO_2_ humidified atmosphere.

To differentiate into M0 phenotype, THP‐1 monocytes were treated with 100 ng/mL phorbol 12‐myristate 13‐acetate (PMA; Sigma‐Aldrich) for 24 h. Following differentiation, cells were washed with phosphate‐buffered saline (PBS) and incubated in fresh PMA‐free complete medium for an additional 24‐h resting period to stabilize the M0 phenotype. For M1 polarization, M0 macrophages were stimulated with 100 ng/mL LPS and 20 ng/mL recombinant human interferon‐γ (IFN‐γ) for 24 h.

### 4.9 Hepatocyte Cell Culture and Lipid Accumulation Analysis

The human hepatocellular carcinoma cell lines HepG2 and Huh7 were procured from ATCC. Cells were cultured in DMEM (High Glucose) supplemented with 10% FBS and 1% penicillin‐streptomycin, maintained at 37°C in a 5% CO_2_ humidified incubator. The medium was refreshed every 2–3 days. For in vitro lipid accumulation assessment, Huh7 cells were cultured to 70% confluence. The medium was then replaced with fresh medium containing a free fatty acid (FFA) mixture of oleic acid (OA) and palmitic acid (PA) at a 2:1 molar ratio (final concentrations: 0.5 mm OA and 0.25 mm PA) for 24 h. Control cells were treated with medium containing an equivalent concentration of fatty acid‐free BSA and ethanol vehicle. Intracellular lipid droplets were visualized using BODIPY 493/503 staining.

### 4.10 Adipocyte and Myoblast Cell Culture and Differentiation

Mouse 3T3‐L1 preadipocytes (American Type Culture Collection, Manassas, VA, USA) and primary SVF‐derived preadipocytes were grown in DMEM containing 10% fetal bovine serum and 1% penicillin/ streptomycin (Thermo Fisher Scientific, Waltham, MA, USA) and incubated in 5% CO2 at 37°C. Primary stromal‐vascular fraction (SVF) cells were extracted as described [5]. Briefly, SVF cells were obtained from iWAT and eWAT of 8–12‐week‐old WT C57BL/6 male mice. Tissues were washed, chopped, and digested in DMEM with 2% BSA and collagenase. After centrifugation and filtration, cells were cultured in DMEM with 10% FBS with media changes every 2–3 days. Once reaching 90%–95% confluence, cells were used for differentiation and other experiments. Preadipocyte differentiation was conducted as described previously [62], cells were treated with a DMEM/IBMX mixture (0.5 mm 3‐isobutyl‐1‐methylxanthine, 10 µm dexamethasone, and 10 µg/ml insulin) for 3–4 days, followed by DMEM supplemented with 10% FBS, 1% P/S, and insulin for an additional 7–10 days. In select experiments, 3T3‐L1 and SVF preadipocytes were exposed to indicated concentrations of CEL. Differentiated 3T3‐L1 adipocytes underwent a 12‐h serum starvation period with FBS‐free BMDM, followed by 400 nm CEL (with DMSO as a control) treatment for 48 h. Subsequently, cells were washed with PBS, fresh DMEM was added, and the conditioned medium (CM) was collected after centrifugation at 500×g for 10 min. The CM suspension was then utilized immediately or stored at −80°C for future use.

C2C12 myoblasts were maintained in high‐glucose DMEM supplemented with 10% FBS and at 37°C in a 5% CO_2_ humidified incubator. Differentiation was induced when cells reached 100% confluence by switching the medium to DMEM containing 2% horse serum. The differentiation medium was refreshed every 48 h until use.

### 4.11 Exosome Isolation and Identification

For the isolation of exosomes from preadipocytes and adipocytes, cells were cultured in DMEM supplemented with exosome‐depleted FBS (System Biosciences, CA, USA). After three washes with PBS, cells were maintained in DMEM with 10% exosome‐depleted FBS for an additional 48 h. The medium was then collected for exosome isolation.

To obtain tissue‐derived exosomes, adipose tissue was minced into approximately 2 mm^3^ pieces and cultured in DMEM with exosome‐depleted FBS for 48 h. The culture medium was collected for exosome purification following a previously described method [63]. In summary, the medium was centrifuged at 2000 x g for 30 min, mixed with an equal volume of 16% PEG, and left to incubate overnight at 4°C. Subsequently, the mixture was centrifuged at 12 000 rpm for 2 h at 4°C. The pellet containing exosomes was resuspended in 100 µL of PBS, and the exosome concentration was determined using a BCA protein assay kit (Thermo Scientific). The size and concentration of freshly isolated exosomes were assessed using a NanoSight instrument (Malvern, UK), and selected samples were analyzed by transmission electron microscopy (TEM) at MISP (Wuhan, China). Exosomes were stored at −80°C until further use. All procedures were conducted on ice or at 4°C to maintain vesicle integrity.

### 4.12 Adipocyte‐Derived Exosome Labeling with DiI/DiR

To detect macrophage uptake of adipocyte‐derived exosomes, adipocyte‐derived exosomes were labeled with DiI/DiR using Sigma's Fluorescent Cell Linker Kit: 30 µg exosomes in 50 µL PBS were mixed with 0.5 µL DiI, incubated in the dark for 30 min, then 100 µL FBS was added. Labeled exosomes were washed twice with DPBS (100 000 × g, 1 h) and resuspended. 10 µg DiI‐labeled SVF exosomes were co‐incubated with macrophage cells in slide chambers for 24 h. Macrophage cells were then washed, fixed in 4% paraformaldehyde, permeabilized with 0.3% Triton X‐100, and washed again. Fluorescence was detected via Image Stream Mark II flow cytometry and Carl Zeiss Jena fluorescence microscopy.

### 4.13 Exosomes Uptake in Peripheral Tissue

DiI‐ and DiR‐labeled adipocyte‐derived exosomes were intraperitoneally injected into HFD‐induced obese mice. After 24 h post‐injection, liver, skeletal muscle, and WAT tissues were snap‐frozen in O.C.T. compound. 5 µm cryosections were fixed on slides, blocked with 1% BSA at room temperature for 40 min, washed with PBS, and treated with Triton X‐100 for 10 min. After washing, DAPI‐containing mounting media were added, coverslips were applied, and slides were incubated at 4°C overnight. Images were captured using a Zeiss LSM 880 laser scanning microscope and processed with ImageJ.

### 4.14 Agomir miR‐5099 Mimic Transfection into Cultured Cells or In Vivo Treatment in Obese Mice

Cy3‐labeled or unlabeled miR‐5099 mimics were transfected into target cells (60%–80% confluent) using Lipofectamine 3000 per the manufacturer's protocol; transfection efficiency was evaluated 24 h later via qPCR and Cy3 fluorescence imaging. For in vivo studies, mice received 2 nmol agomir miR‐5099 mimics via tail vein injection every other day for 8 weeks or a specified duration. Mice were monitored for physiological responses, and tissues were collected post‐treatment for downstream analyses.

### 4.15 The Treatment of CEL, Exosomes, and miR‐5099 Mimic In Vitro in Cultured Cells and In Vivo in Mice

CEL was administered to 3T3‐L1 and iWAT‐SVF derived primary adipocytes at a concentration of 400 nm for 48 h. Macrophages (BMDM and RAW264.7) were treated with concentrations of 0.1, 0.5, 1.0, and 2.0um for 24 h. DMSO was used as the vehicle control.

In animal studies, concentrations of CEL at 100 or 500ug/kg body weight were used, with the control being an equivalent volume of DMSO dissolved in PBS.

For exosomes, CEL‐treated adipocyte‐derived exosomes were applied to cells at a concentration of 30 µg per 1 × 10^5^ cells. In animals, the concentration was 50ug/mouse/time (in 100 µL sterile PBS, administered three times per week), following slight modifications from previous studies [64, 65, 66]. The control exosomes were derived from DMSO‐treated adipocytes.

For miR‐5099 mimics, the control was the synthesized NC (Negative Control) miRNA. The concentration for both NC and miR‐5099 mimics in cell treatment was 100 nm. For animal treatment, the concentration was 2.5 nmol per mouse per injection.

### 4.16 Glucose Uptake and Production Assay

Glucose uptake was measured using the fluorescent analog 2‐NBDG (MCE, HY‐116215). Cells were serum‐starved for 6 h, then incubated with 100 µL 50 µm 2‐NBDG (±100 nm insulin) at 37°C for 30 min. After washing with ice‐cold PBS to remove excess dye, fluorescence intensity was detected via a microplate reader, and images were captured using a Nikon A1R confocal microscope; the assay was repeated 3 times independently. For glucose production, hepatocytes were serum‐starved overnight, washed twice with PBS, and incubated in glucose‐free DMEM supplemented with glucagon (200 ng/mL), insulin (100 nM), or both for 4 h at 37°C. Medium glucose concentration was quantified with a glucose assay kit according to the manufacturer's instructions (Jiancheng, A154‐1‐1). Glucose production was normalized to total protein, with experiments performed in triplicate and repeated independently at least 3 times.

### 4.17 RNA Extraction and Quantitative Real‐Time Polymerase Chain Reaction (RT‐PCR) Analysis

Total cellular RNA was extracted using TRIzol reagent (Thermo Fisher Scientific), and 500 ng of this RNA was used for reverse transcription. For miRNA RT‐PCR, exosomal RNA was isolated with an exosome RNA isolation Kit (Rengenbio, China; EXORNA30C‐1), and cDNA was synthesized using 5× miRNA primers and the miRNA First Strand cDNA Synthesis Kit (Sangon, China, B532451‐0020). Quantitative RT‐PCR was conducted on a QuantStudio 7 Flex Real‐Time PCR system (Applied Biosystems) with specific primers and SYBR Green (QuantiTect RT Kit; Qiagen). S16 was used as the internal control for mRNA, while U6 served as the internal control for miRNA normalization.

### 4.18 MiR‐5099 Target Gene Prediction and Luciferase Reporter Assay

MiR‐5099 target genes were predicted using TargetScan Mouse 8.0 (https://www.targetscan.org/mmu_80/). To confirm c‐MET as a potential target, a luciferase reporter assay was performed with the pmirGLO Dual‐Luciferase Vector (Promega, E1330). The c‐MET 3’untranslated region (UTR), harboring either wild‐type (5’…GAAAGAUUUUGUUCUGAUCUAA…) or mutated (5’…GAAAGAUUUUGUUCUAUGGAUC…) miR‐5099 binding sites, was cloned downstream of the firefly luciferase gene (luc2). HEK293 cells were co‐transfected with these reporter constructs and either miR‐5099 mimic or negative control mimic. After 24 h, firefly luciferase activity (primary reporter for mRNA regulation) was measured and normalized to Renilla luciferase activity to account for transfection efficiency.

### 4.19 Western Blot Analysis

For Western blot analysis, protein lysates were extracted from lysed cells utilizing RIPA lysis buffer supplemented with a protease inhibitor cocktail (Sigma–Aldrich, P8340). In the case of phosphorylated proteins, a phosphatase inhibitor cocktail (Cell Signaling, 5870) was additionally incorporated into the lysis buffer. The proteins were fractionated using 10% SDS‐PAGE gels and transferred onto a Nitrocellulose Membrane (Bio‐Rad, 1620115). Primary antibodies employed for Western blotting and their respective dilutions were specified in the “Reagents and Antibodies” section. Protein bands were visualized using ECL Western Blotting Reagents (Cytiva, RPN2232). The ChemiDoc system from Bio‐Rad was utilized for gel and immunoblot data acquisition, while ImageJ was employed for image processing and semi‐quantification, with blots normalized relative to loading controls.

### 4.20 RNA‐Sequencing Data Analysis

Total RNA was isolated from the adipose tissues of the specified mice using the RNeasy Extraction Kit (Qiagen, 74106). RNA sequencing was carried out on the Illumina NovaSeq 6000 platform with Paired‐End 150 (PE150) sequencing technology. The sequence reads were subjected to strict quality control using FastQC. Subsequently, the RNA‐seq data were aligned to the GRCm39 mouse reference genome with Bowtie2 (Version 2.5.2), involving the trimming of 10 bases from the 5' end of the nucleotide chain. Reads were filtered to guarantee a minimum final length of 25 bases after trimming. The paired‐end read counts were measured using featureCounts (Version 2.0.6). Differential expression analysis of the read counts was then performed with the DESeq2 package, and sample normalization was achieved through variance stabilizing transformation (VST). Genes exhibiting a fold change exceeding 2 and a *p*‐value < 0.05 were identified as differentially expressed genes. For analyzing the Gene Ontology (GO) pathway of Differentially Expressed Genes (DEGs) or conducting Gene Set Enrichment Analysis (GSEA), the clusterProfiler package in R was used (version 4.1.2).

### 4.21 Exosomal miRNA Sequencing

Exosomes were isolated from the culture medium of specified cells using the RiboTM Exosome Isolation Reagent (Ribobio, China; C‐10120‐1) per the manufacturer's instructions. Exosomal RNA was extracted with MagZol (Magen, R480101) following the provided protocol and submitted to Ribobio Co. Ltd (China) for sequencing on an Illumina HiSeq 2500 platform with single‐end 50 bp reads. TargetScan and miRPathDB were used to predict target genes of selected miRNAs, while KOBAS was employed for further Gene Ontology (GO) and KEGG (Kyoto Encyclopedia of Genes and Genomes) pathway analyses.

### 4.22 Single Cell Sequencing Analysis

Epididymal adipose tissues underwent enzymatic digestion to yield single‐cell suspensions. These suspensions were used to create single‐cell libraries with the SeekOne Digital Droplet Single Cell 3′ library preparation kit (SeekGene, K00202). Subsequently, single‐cell RNA sequencing libraries were generated also using the SeekOne Digital Droplet Single Cell 3′ kit (SeekGene, K00202) following the manufacturer's protocols. The cells were mixed with reverse transcription reagents and loaded into SeekOne chips along with Barcoded Hydrogel Beads and partitioning oil to form emulsion droplets. The sequencing was performed on an Illumina NovaSeq 6000 platform (PE150). Gene Ontology (GO) and Gene Set Enrichment Analysis (GSEA) analyses were carried out using DESeq2 and clusterProfiler.

### 4.23 LC‐MS/MS Proteomics Analysis

Proteome profiles were analyzed via liquid chromatography‐tandem mass spectrometry (LC‐MS/MS). Briefly, washed exosomes were dissolved in ice‐cold SDS extraction buffer (0.02 m Tris/HCl, pH 6.8, 2% SDS, 0.1 m DTT). The suspension was sonicated with a Bioruptor Plus (30 sec on/off cycles at 4°C for 4 cycles) to ensure complete lysis and genomic DNA fragmentation, then incubated at 56°C for 30 min and cooled. Samples were centrifuged at 14 000 rpm for 30 min at 4°C, and the supernatant was collected as protein extract. Protein concentration was measured using the Bio‐Rad RC‐DC assay to achieve >0.5 µg/µL, and the extract was subsequently subjected to LC‐MS/MS proteomic analysis.

The peptides were desalted using C18 StageTips, dried in a vacuum centrifuge, and reconstituted in 0.1% formic acid. LC‐MS/MS analysis was conducted on an Easy‐nLC 1200 system coupled to a Q Exactive Orbitrap mass spectrometer (Thermo Fisher Scientific). Peptides were chromatographically separated on a C18 analytical column (75 µm × 15 cm, 3 µm) using a linear gradient of 5%–35% solvent B (0.1% formic acid in acetonitrile) over 60 min. The mass spectrometer operated in data‐dependent acquisition (DDA) mode with a full MS scan range of 350–1800 m/z (resolution 70 000), selecting the top 15 most intense ions for HCD fragmentation. Raw data were analyzed using MaxQuant software, searching against the UniProt Mouse database with a false discovery rate (FDR) of 1% for both proteins and peptides.

### 4.24 Dual‐Luciferase Reporter Assay for miR‐5099 Promoter Activity

A dual‐luciferase reporter assay was conducted to evaluate the activity of the miR‐5099 promoter. The promoter sequence of the pre‐miR‐5099 was amplified and cloned into the pGL3‐Basic luciferase reporter vector (Promega, Madison, WI, USA) to generate the miR‐5099 promoter reporter.

3T3‐L1 cells were co‐transfected with the pGL3‐miR‐5099 promoter construct and the pRL‐TK (Renilla luciferase control vector) using Lipofectamine 3000 (Invitrogen) following the manufacturer's instructions. To explore the regulatory impact of GATA2, cells were co‐transfected with Gata2 siRNA (siRNA GATA‐2: 5′‐CCGACGAGGTGGATGTCTT‐3′) or a non‐targeting negative control (NC) siRNA. After 24 h post‐transfection, the medium was refreshed, and cells were treated with 400 nm CEL for an additional 48 h. Following treatment, cells were harvested, lysed, and luciferase activities were measured using the Dual‐Luciferase Reporter Assay System (Promega). Firefly luciferase activity was normalized to Renilla luciferase activity to adjust for transfection efficiency. Data are presented as relative luciferase activity compared to the empty pGL3‐Basic vector or the control group.

### 4.25 NF‐κB Luciferase Reporter Assay

To evaluate NF‐κB signaling activation, RAW 264.7 cells were transiently co‐transfected with 100 ng of the NF‐κB firefly luciferase reporter plasmid (pNFκB‐Luc), 10 ng of the pRL‐TK Renilla luciferase internal control plasmid (Promega), and 50 nm of the miR‐5099 mimic or negative control using Lipofectamine 3000 (Invitrogen) following the manufacturer's instructions. After 24 h post‐transfection, the cells were stimulated with LPS (100 ng/mL) and IFN‐γ (10 ng/mL) for 24 h. Firefly and Renilla luciferase activities were measured using the Dual‐Luciferase Reporter Assay System (Promega). The data were normalized to Renilla luciferase activity to adjust for transfection efficiency and presented as relative luciferase units (RLU).

### 4.26 Chromatin Immunoprecipitation (ChIP) Assay

ChIP analysis was conducted according to the described protocol [62]. Briefly, cells were lysed, nuclei were isolated, crosslinked with 1% formaldehyde, and lysed in a buffer containing 1% SDS, 10 mm EDTA, and 50 mm Tris (pH 8.1). The chromatin DNA was sonicated on ice to an average length of 500 bp. The sonicated supernatant was diluted 10‐fold in a ChIP dilution buffer (0.01% SDS, 1.1% Triton X‐100, 1.2 mm EDTA, 16.7 mm Tris‐HCl pH 8.1, and 167 mm NaCl). The precleared chromatin was incubated with the specific antibody or IgG control overnight at 4°C. The immunoprecipitations were then recovered by incubating with protein G‐agarose (Upstate Biotechnology) for 2 h at 4°C, followed by low‐speed centrifugation. The washed pellets were subjected to reverse crosslinking. The DNA was used for quantitative PCR analysis.

### 4.27 Statistical Analysis

All collected data were analyzed using Microsoft Excel 2010 and GraphPad Prism version 10.1.2. Normality of distribution was tested with the Shapiro–Wilk method in GraphPad Prism. Homogeneity of variance was assessed using either Levene's test or Bartlett's test. For comparisons between two groups, an unpaired two‐tailed *t*‐test was used. When analyzing more than two groups, one‐way analysis of variance (ANOVA) was applied, followed by Tukey's post‐hoc test for multiple comparisons between all groups, and Dunnett's post‐hoc test was used to compare treatment groups against the control group. Statistical significance was set at *P* < 0.05. All reported data came from multiple independent biological replicates, with exact sample sizes provided in the figures or their legends. Data are expressed as means ± standard error of the mean (SEM) unless specified otherwise.

## Author Contributions

P.T. conceived, designed, and performed the experiments, interpreted the results, and drafted and reviewed the manuscript. L.T. designed and performed the experiments and interpreted the results. H.X. performed the experiments and interpreted the results. D.Z. performed the experiments, interpreted the results, and reviewed the manuscript. J.L. designed and performed the experiments and interpreted the results. H.F. performed the experiments and interpreted the results. J.C. performed the experiments and interpreted the results. S.L. interpreted the results and reviewed the manuscript. L.D. conceived and designed the studies, interpreted the results, drafted, edited, and reviewed the manuscript. L.W. conceived and designed the studies, interpreted the results, and drafted, edited, and reviewed the manuscript.

## Conflicts of Interest

The authors declare no conflicts of interest.

## Supporting information




**Supporting File**: advs74443‐sup‐0001‐SuppMat.docx.

## Data Availability

The data that support the findings of this study are available from the corresponding author upon reasonable request.
