# Supplementary material for: Adipocyte‐Derived Exosomal miR‐5099 Mitigates M1 Macrophage Polarization and Adipose Inflammation via c‐Met/NF‐κB Axis to Improve Metabolic Health
Source: Adv Sci (Weinh). 2026 Feb 21;13(24):e18902. doi: 10.1002/advs.202518902 (PMC13116018; doi:10.1002/advs.202518902)
Supplement: Supplementary file 1 — Supporting File: advs74443‐sup‐0001‐SuppMat.docx. [file ADVS-13-e18902-s001.docx]

**Supporting Information**

**Adipocyte-Derived Exosomal miR-5099 Mitigates M1 Macrophage Polarization and Adipose Inflammation via c-Met/NF-κB axis to Improve Metabolic Health**

Ping Tang^1,2^, Lixin Tai^1,2^, Hongxia Xu^1,2^, Dongliang Zhu^1,2^, Jiajia Li^1,2^, Hao Feng^1, 2^, Jinghui Cui^1,2^, Shuhai Lin^3^, Li-jun Di^1,2,4^, Li Wang^1,2,4^

**Supplementary Tables and Figures**

| **Table 1： Key source** | | |  |
| --- | --- | --- | --- |
| **REAGENT or RESOURCE** | | |  |
| **Antibodies** | | |  |
| CD63 Rabbit Monoclonal Antibody | Cell signaling technology | Cat# 10112S | |
| Mouse monoclonal anti-HSP70 | Santa Cruz | Cat# sc-24 | |
| Anti-CD9 Antibody (C-4) （200 µg/ml） | Santa Cruz | Cat# sc-13118 | |
| Anti-GAPDH | Cell signaling technology | Cat# 2118S (1:2000 dilution) | |
| Flotillin-1 rabbit antibody | Cell Signaling technology | Cat# 18634S | |
| Anti-Phospho-AKT Ser473 | Cell signaling technology | Cat# 9271S (1:1000 dilution) | |
| Anti-pan AKT | Cell signaling technology | Cat# 4691S (1:2000 dilution) | |
| Alexa FluorR 594 anti-Nos2 (iNOS) Antibody | Biolegend | Cat# 696804 | |
| F4/80 Monoclonal Antibody (BM8) | eBioscience™ | Cat# 14-4801-82 | |
| Phospho-NF-κB p65 | Cell signaling technology | Cat# 3033S | |
| NF-κB p65 | Cell signaling technology | Cat# 8242S | |
| NF-kappaB2 p100/p52 Antibody #4882 | Cell signaling technology | Cat# 4882S | |
| GATA2 Polyclonal antibody | Proteintech | Cat# 11103-1-AP | |
| HSP70 Antibody | Cell signaling technology | Cat# 4872S | |
| PerCP/Cyanine5.5 anti-mouse CD11c Antibody | Biolegend | Cat# 117328 | |
| Calnexin Antibody | Santa Cruz | Cat# sc-23954 | |
| **Chemicals, Peptides, and Recombinant Proteins** | | |  |
| Murine M-CSF-50UG | PeproTech | Cat# AF-315-02-50ug | |
| Celastrol 1g | BOC Sciences | Cat# 34157-83-0 | |
| Dil(1,1′-Dioctadecyl-3,3,3′,3′-tetramethylindocarbocyanine perchlorate) | Sigma-Aldrich | Cat# 41085-99-8 | |
| Ethyl alcohol, puro, 200 proof, anhydrous | Sigma-Aldrich | Cat# 64-17-5 | |
| GW4869(N,N′-Bis[4-(4,5-dihydro-1H-imidazol-2-yl)phenyl]-3,3′-p-phenylene-bis-acrylamide dihydrochloride) | Sigma-Aldrich | Cat# 6823-69-4 | |
| Lenti-X Concentrator 500 mL | Takara | Cat# 631232 | |
| Lipofectamine™ 3000 Transfection Reagent | Invitrogen | Cat# L3000015 | |
| Poly (ethylene glycol) BioUltra, for molecular biology, 8,000 | Sigma-Aldrich | Cat#25322-68-3-1kg | |
| TRIzol™ Reagent | Invitrogen | Cat# 15596018 | |
| Universal PCR Primer R (10 μM) | Sangon Biotechnology | Cat# B661601-0002(200ul) | |
| Triglyceride (TG) Content Assay Kit | Solarbio | Cat# BC0625 100T/96S | |
| [Total cholestenone (TC) Content Assay Kit](file:///C:\\Users\\TANGPINGWJ\\AppData\\Local\\youdao\\dict\\Application\\8.10.6.0\\resultui\\html\\index.html#javascript:;) | Solarbio | Cat# BC1985 100T/96S | |
| 2-NBDG | MedComExpress | Cat# 186689-07-6 | |
| Glucagon | MedComExpress | Cat# 64790-15-4 | |
| Cell Counting Kit-8 | Beyotime | Cat# C0038 | |
| Recombined human insulin (Insulin solution human) used in ITTs | Sigma-Aldrich | Cat# 11061-68-0 | |
| Insulin used in glucose uptake and hepatic glucose production assays | Sigma-Aldrich | Cat# I9278 | |
| IBMX（3-Isobutyl-1-methylxanthine） | Sigma-Aldrich | Cat# 28822-58-4 | |
| D-Glucose | Sigma-Aldrich | Cat# G8270-100G | |
| [Dexamethasone](https://www.sigmaaldrich.com/US/en/substance/dexamethasone3924650022) | Sigma-Aldrich | Cat# 50-02-2 | |
| Insulin | Sigma-Aldrich | Cat# 11070-73-8 | |
| Rosiglitazone | Sigma-Aldrich | Cat# 122320-73-4 | |
| Collagenase Type I | Gibco | Cat# 9001-12-1 | |
| 10x Red Blood Cell (RBC) Lysis | Abcam | Cat# ab204733 | |
| RIPA buffer (10x) | Cell Signaling Technology | Cat# 9806 | |
| DiR（Cy7 DiC18） | MedComExpress | Cat#100068-60-8 | |
| High-fat diet containing 60 kcal% fat | Research Diets | Cat# D12491 | |
| PrimeScript RT Master Mix (Perfect Real Time) | Takara | Cat# RR036A | |
| SYBR Green supermix | Takara | Cat# 639676 | |
| Exosome-depleted FBS （250ml) | System Biosciences (SBI) | Cat# EXO-FBS-250A-1 | |
| lipopolysaccharides | Sigma-Aldrich | Cat# SMB00610-1MG | |
| Recombinant Murine IFN-γ | Peprotech | Cat# 315-05-100 | |
| Human IFN-gamma Recombinant Protein | Peprotech | Cat# 300-02-100UG | |
| PMA | Sigma-Aldrich | Cat# 16561-29-8 （P1585） | |
| Recombinant murine IL-4 | Peprotech | Cat# 214–14 | |
| Oleic acid | MedComExpress | Cat# 112-80-1 | |
| Palmitic acid | MedComExpress | Cat# 57-10-3 | |
| **Critical Commercial Assays** | | |  |
| miRNA First Strand cDNA Synthesis (Tailing Reaction) 20 RXNS | Sangon Biotechnology | Cat# B532451-0020 | |
| Dual-Glo® Luciferase Assay System | Promega | Cat# E2920 | |
| Glucose production assay kit | Nanjing Jiancheng | Cat# A154-1-1 | |
| Urea Assay Kit | Nanjing Jiancheng | Cat# C013-1-1 | |
| Aspartate aminotransferase Assay Kit | Nanjing Jiancheng | Cat# C010-2-1 | |
| Alanine aminotransferase Assay Kit | Nanjing Jiancheng | Cat# C009-2-1 | |
| Lactate dehydrogenase assay kit | Nanjing Jiancheng | Cat# A020-2-2 | |
| Triglyceride assay kit | Nanjing Jiancheng | Cat# A110-1-1 | |
| Total cholesterol assay kit | Nanjing Jiancheng | Cat# A111-1-1 | |

**Table 2：Primers table**

| **Gene name** | **Oligonucleotides** |
| --- | --- |
| M-Actb_F | TCAGCAAGCAGGAGTACGATG |
| M-Actb_R | AACGCAGCTCAGTAACAGTCC |
| M-iNOS-F | CCAAGCCCTCACCTACTTCC |
| M-iNOS-R | CTCTGAGGGCTGACACAAGG |
| M-IL-6-F | CCAGAGATACAAAGAAATGATGG |
| M-IL-6-R | ACTCCAGAAGACCAGAGGAAAT |
| M-IL-1β-F | CTCCATGAGCTTTGTACAAGG |
| M-IL-1β-R | TGCTGATGTACCAGTTGGGG |
| M-TNFα-F | CATCTTCTCAAAATTCGAGTGA |
| M-TNFα-R | TGGGAGTAGACAAGGTACAACCC |
| M-Ccl2_F | CTGGAGCATCCACGTGTTGG |
| M-Ccl2_R | TGGTGAATGAGTAGCAGCAGG |
| M-s16-F | GATCGAGCCGCGCACG |
| M-s16-R | CAAATCGCTCCTTGCCCA |
| M-Fizz1-F | CCTGCTGGGATGACTGCTA |
| M-Fizz1-R | TGGGTTCTCCACCTCTTCAT |
| M-Mgl1-F | CAGAATCGCTTAGCCAATGTGG |
| M-Mgl1-R | TCCCAGTCCGTGTCCGAAC |
| M-Mgl2-F | TTCAAGAATTGGAGGCCACT |
| M-Mgl2-R | CAGACATCGTCATTCCAACG |
| M-Ym1-F | AGAAGGGAGTTTCAAACCTGGT |
| M-Ym1-R | GTCTTGCTCATGTGTGTAAGTGA |
| M-Arg1-F | CTCCAAGCCAAAGTCCTTAGAG |
| M-Arg1-R | AGGAGCTGTCATTAGGGACATC |
| M-c-Met_F | GGAACTGGCTACTGCTCTGG |
| M-c-Met_R | GGTGGGAGCCTTCATTGTGA |
| M-Coch_F | ACAAGGCTGTGTGTCGGAAT |
| M-Coch_R | TGTTCGTGCGTACAGAGCTT |
| M-Cd33_F | TTCTTGGGCTCTGTCTCGTG |
| M-Cd33_R | TGCAGTTGGAGATAGGCAGTG |
| M-Inhba_F | AAATCAGAACGCCTCCGCTA |
| M-Inhba_R | TCCCGAGTGTAGAGTTCGGT |
| M-Siglece_F | ATGGGGACCTTTGTGGGTTC |
| M-Siglece_R | TGTAGGATCTTACTGCCAGGAG |
| M-Lad1_F | CCCTGAGAAGACGCTTGTGT |
| M-Lad1_R | TTCTTTGGCGTCAGCTTTGC |
| M-Mxd1_F | TGGATCCCCACCCGTTAGAT |
| M-Mxd1_R | CACAGAGAATGGCGGTGTCT |
| M-Gja1_F | GAGCAGAGTTCTCCCCGTG |
| M-Gja1_R | AAGGCTGCCCTGTACGTTTT |
| gRab27a-1# F | CACCGCTAAGGGATAGAGCACAGCG |
| gRab27a-1# R | AAACCGCTGTGCTCTATCCCTTAGC |
| gRab27a-2# F | CACCGGTTGGGAATCTAGCACTGCA |
| gRab27a-2# R | AAACTGCAGTGCTAGATTCCCAACC |
| gRab27b F | CACCGATGGGCTAGCTATTCCCCCG |
| gRab27b R | AAACCGGGGGAATAGCTAGCCCATC |
| gRab27b F | CACCGGCATCCGATTATGTGAACAC |
| gRab27b R | AAACGTGTTCACATAATCGGATGCC |
| Rab27b-F | CTCAGGAGTCGGGAAGACAAC |
| Rab27b-R | TCCCAAAGCTGCAGATGTACC |
| Rab27a-F | AAGGGATAGAGCACAGCGAG |
| Rab27a-R | TGCAGTGTAGCGTCCTTAGC |
| Sg5099-1#-F | CACCGGTAATGGGCTTAGATCGATG |
| Sg5099-1#-R | AAACCATCGATCTAAGCCCATTACC |
| sgc-Met-1# F | CACCGGGAAGTTCACATGAGGAGTG |
| sgc-Met-1# R | AAACCACTCCTCATGTGAACTTCCC |
| sgc-Met-2# F | CACCGATTCAAGACCGGGCCCGTGT |
| sgc-Met-2# R | AAACACACGGGCCCGGTCTTGAATC |
| gDicer F -1 | CACCGACCATGGACAGTGTACGGT |
| gDicer R -1 | AAACACCGTACACTGTCCATCGGTC |
| gDicer F -2 | CACCGAGATGCGTAAACGGATCACT |
| gDicer R -2 | AAACAGTGATCCGTTTACGCATCTC |
| M-Dicer-F | GAGTCTCTTGCTGGTGCCAT |
| M-Dicer-R | TCTCCGCTGGGCTAAACTTG |
| M-Gata2-F | TGGGCTCTACCACAAGATGA |
| M-Gata2-R | GCCATAAGGTGGTGGTTGTC |
| Gata2-Chip-F | GAGTCCATTTCATGGTGCTGA |
| Gata2-Chip-R | TGATGAGTCCCAACTCCAAAG |
| Homo-iNOS-F | CAGGACTCACAGCCTTTGGA |
| Homo-iNOS-R | CTGGATGTCGGACTTTGTAGATT |
| Homo-IL6-F | ACTGGTCTTTTGGAGTTTGAGG |
| Homo-IL6-R | GCTGGCATTTGTGGTTGG |
| Homo-TNFα-F | ATGTTGTAGCAAACCCTCAAGC |
| Homo-TNFα-R | TCTGGTAGGAGACGGCGATG |
| Homo-IL1β-F | TGCTCAAGTGTCTGAAGCAG |
| Homo-IL1β-R | TGGTGGTCGGAGATTCGTAG |
| Homo-actin-F | CTGGAACGGTGAAGGTGACA |
| Homo-actin-R | AAGGGACTTCCTGTAACAACGCA |

**Supplementary figures**

**
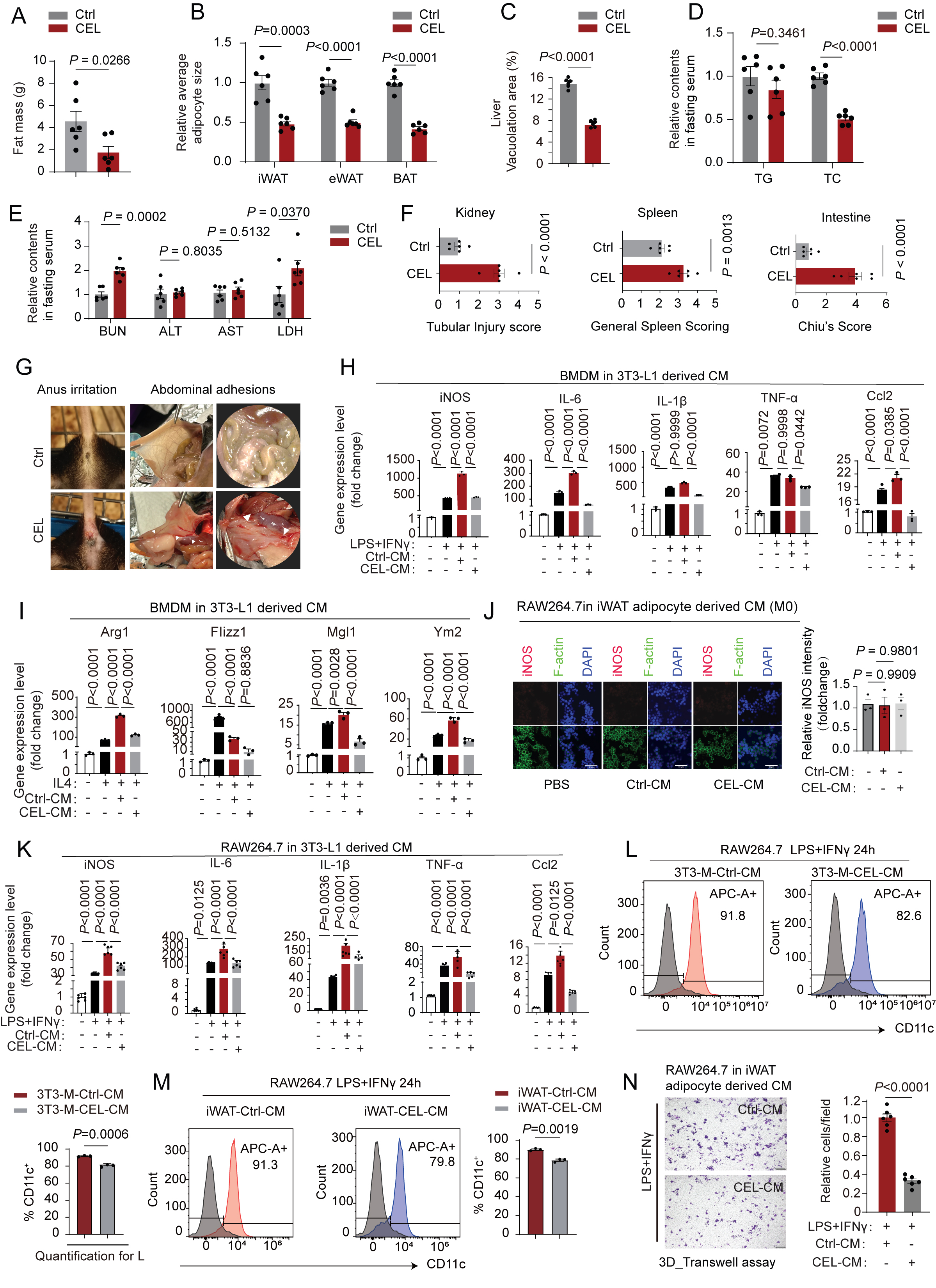
Supplementary Figure 1. The impact of CEL treatment on the metabolic and organ health of obese mice, and the influence of adipocyte-derived CM on the status of macrophages. Male C57Bl/6J mice (6-8 weeks) were induced to obesity by a high-fat diet (HFD) for 8 weeks, followed by a 21-day daily injection of CEL** (intraperitoneal daily injections with indicated doses)**, these mice were used for the experiments in Fig. 1A-K,** sFig. 1A-K and sFig. 2G-I (n=6). **A.** Fat mass of indicated obese mice (n=6). **B-C.** Quantification of average adipocyte size from H&E-stained sections of iWAT, eWAT, BAT, and liver microvesicular vacuoles (C) for main Fig. 1E. **D.** Fasting plasma TG and TC levels in indicated obese mice (n=6). **E.** Serum BUN, ALT, AST and LDH levels (n=6). **F.** Scoring of H&E staining of kidney, spleen and intestine (n=6). **G.** Representative images of mice with irritation or adhesions of anal region and abdominal viscera from indicated mice. **H-I.** Quantification of M1 (H) and M2 (I) marker gene expression in BMDMs cultured in CM collected from 3T3-1 adipocytes without or with CEL treatment (for main Fig. 1J-1K) (n=6). **J-K.** IF staining and quantification for iNOS (J) and quantification of M1 marker gene expression (K, for main Fig. 1L) in RAW264.7 cells treated with adipocyte-derived CM (n=6). Scale bar, 50 μm. **L-M.** Flow cytometry plot and quantification of CD11c⁺ RAW264.7 macrophages with LPS/IFNγ stimulation (100ng/mL; 20ng/mL, 24h) cultured in CM harvested from 3T3-L1 adipocytes (L) or iWAT SVF differentiated adipocytes (M) with or without CEL treatment (0.4 μM, 48 h) (n=3). **N.** Representative images and quantification of migration analysis of RAW264.7 macrophages with LPS/IFNγ stimulation (100ng/mL; 20ng/mL, 48h) cultured in CM harvested from 3T3-L1 adipocytes with or without CEL treatment by 3D transwell assay (n=6). Scale bar, 200 μm. Comparison between two groups was evaluated using an unpaired two-tailed t-test. One-way ANOVA with Tukey’s post-hoc test was used for comparing more than two groups. Data are mean ± SEM.


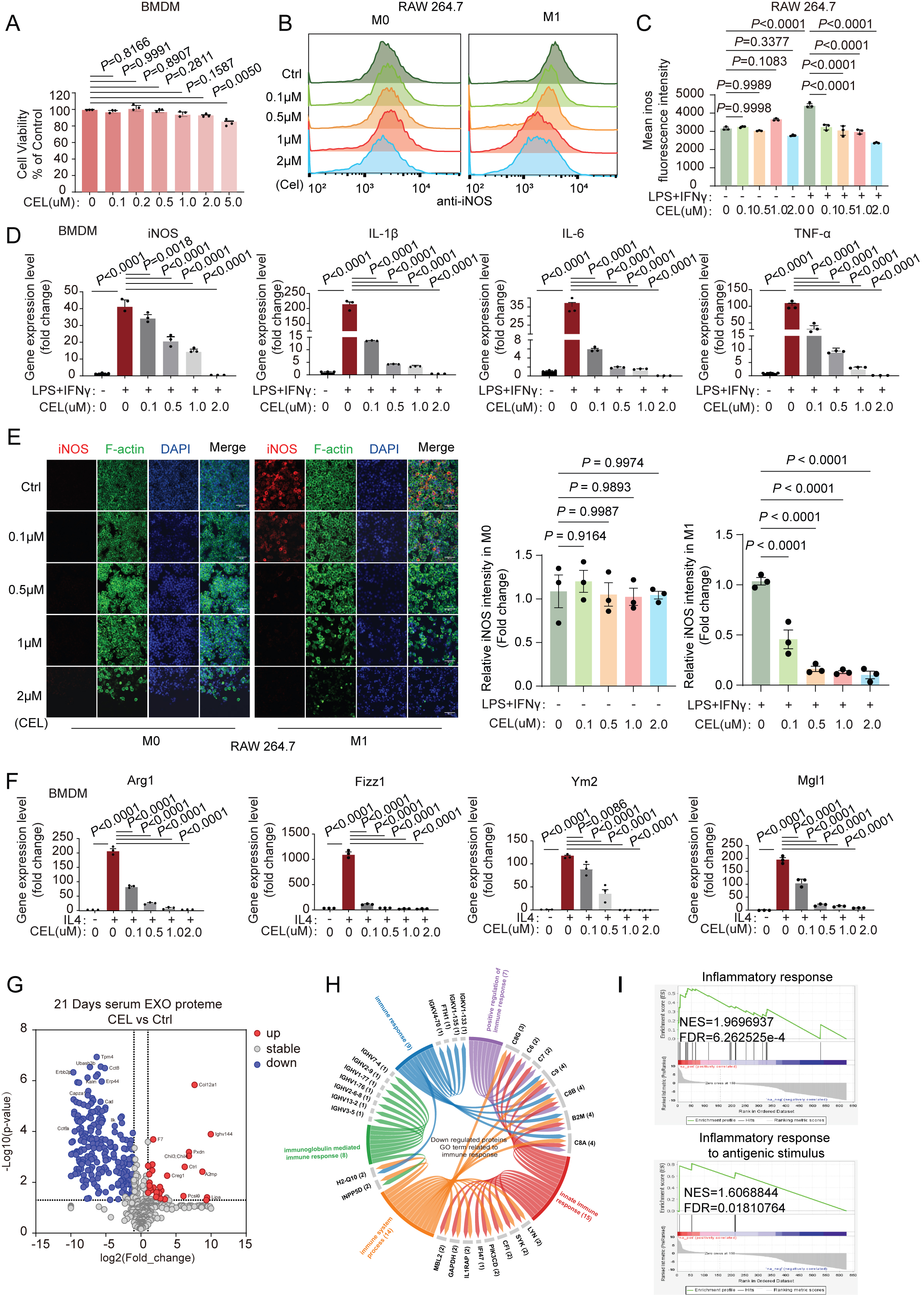


**Supplementary Figure 2. Direct CEL treatment suppresses macrophage differentiation and remodels the serum EXO proteome of obese mice. A.** Cell viability of BMDM macrophages following 48 h CEL treatment with indicated dosages (n=3). **B–C.** Flow cytometry to quantify iNOS⁺ cells in CEL-treated RAW264.7 macrophages (n=6). **D-F**. The expression analysis of M1 marker genes in BMDM (D), iNOS staining in RAW264.7 macrophages (E) and the expression analysis of M2 marker genes in BMDM (F) with or without CEL treatment (n=3). Scale bar, 50 μm. **G-I**. Proteomics analysis of serum EXOs from CEL-treated obese mice (from main Fig. 1A). Gene Ontology (GO) (H) and Gene Set Enrichment Analysis (GSEA) (I) on the downregulated EXO proteins in CEL-treated versus vehicle treated obese mice (n=3). One-way ANOVA with Tukey’s post-hoc test was used for comparing more than two groups. Data are mean ± SEM.


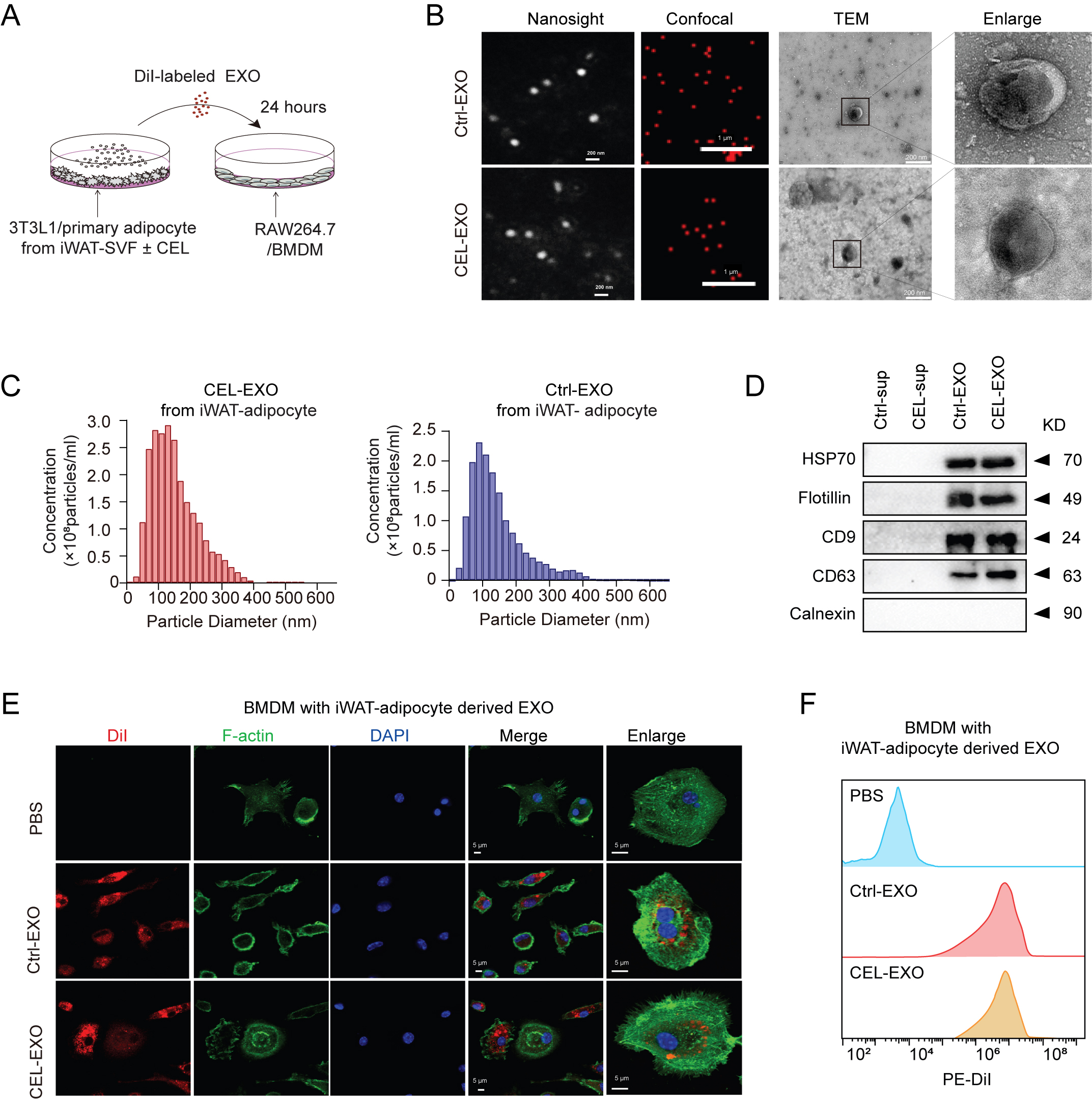


**Supplementary Figure 3. Characterization of EXOs and validation of the uptake of adipocyte-derived EXOs by BMDMs.
A.** Schematic of the approach illustrating EXOs isolation from CEL-treated iWAT-adipocytes. **B.** Representative EXO images by Nanosight (left), Scale bar, 200 nm, confocal microscopy (middle), Scale bar, 1 μm, and TEM (right), Scale bar, 200 nm. **C.** Nanoparticle tracking analysis for size distribution and concentration profile of iWAT-adipocyte–derived EXOs. **D.** Immunoblot of EXO positive and negative markers in iWAT-adipocyte derived EXOs. **E.** Representative images depicting the uptake of DiI-labeled EXOs by BMDMs after 24 hours of incubation, captured using confocal microscopy.Scale bar, 5 μm. **F.** Flow cytometry to validate the uptake of DiI-labeled EXOs by BMDMs. All data represent at least three independent experiments.


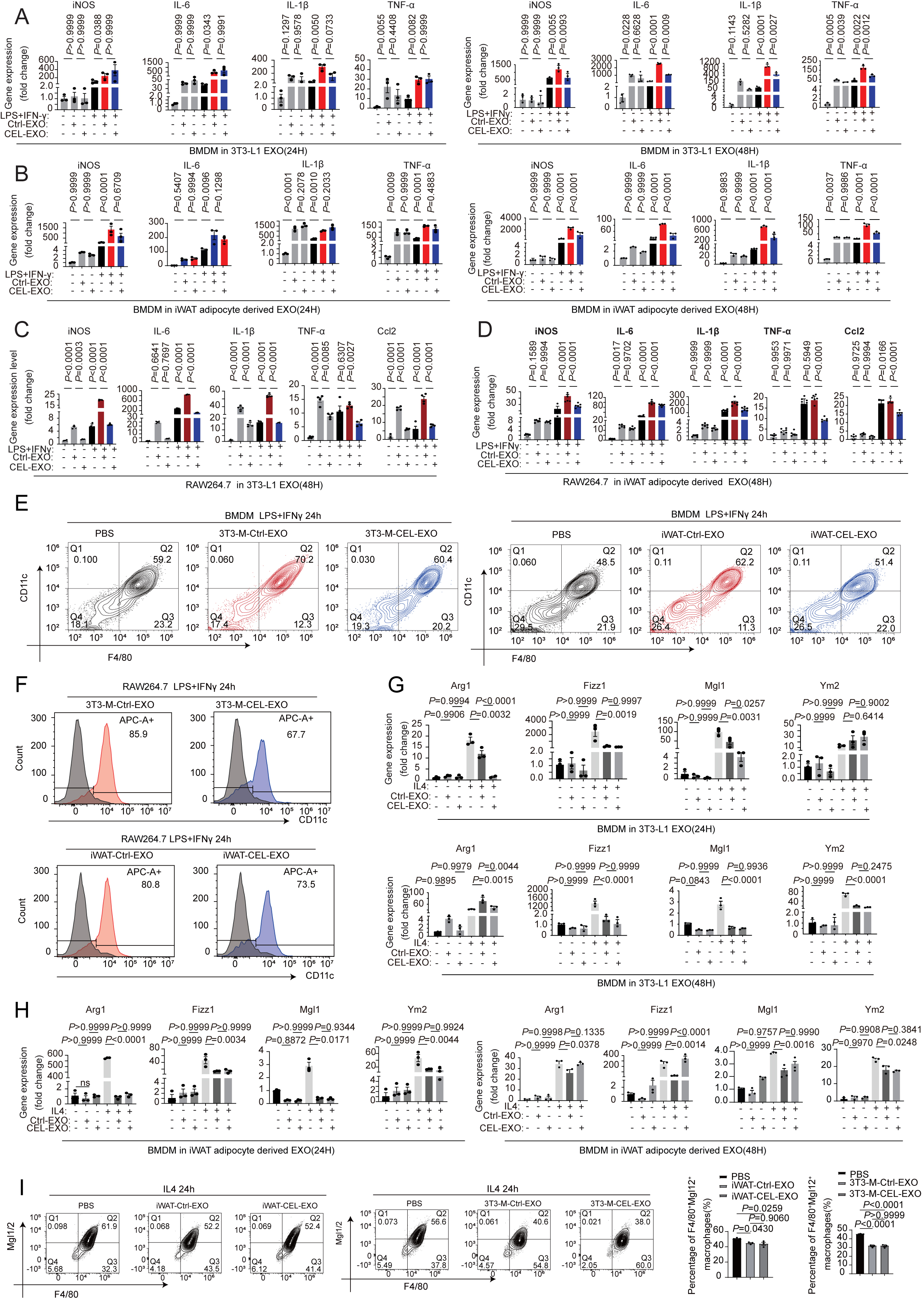


**Supplementary Figure 4. CEL-treated adipocyte-derived EXOs modulate macrophage polarization. A–D.** The expression analysis of M1 markers by RT-PCR in BMDMs (A, B) and RAW264.7 cells (C, D) treated for 24 h with EXOs (50 µg/mL), derived from 3T3-L1 (A, C) adipocytes or iWAT SVF differentiated adipocytes (B, D), which were either pretreated with DMSO (Ctrl-EXO) or CEL (400 nM, 24, or 48 h; CEL-EXO), n=3 for A and B; n=4 for C; n=6 for D. **E–F.** Flow cytometry to detect CD11c⁺F4/80⁺ M1 macrophages in BMDMs (E) and RAW264.7 cells (F) treated with or without CEL-EXO (quantification in main Fig. 2E-F) (n=3). **G–H.** The expression analysis of M2 markers by RT-PCR in BMDMs treated with 3T3-L1–derived (G) or iWAT SVF differentiated adipocytes (H) exosomes (n=3). **I.** Flow cytometry and quantification of F4/80⁺/Mgl1/2^+^ M2 BMDMs treated with CEL-EXO as indicated (n=3). One-way ANOVA with Tukey’s post-hoc test s was used for comparing more than two groups. Data are mean ± SEM.


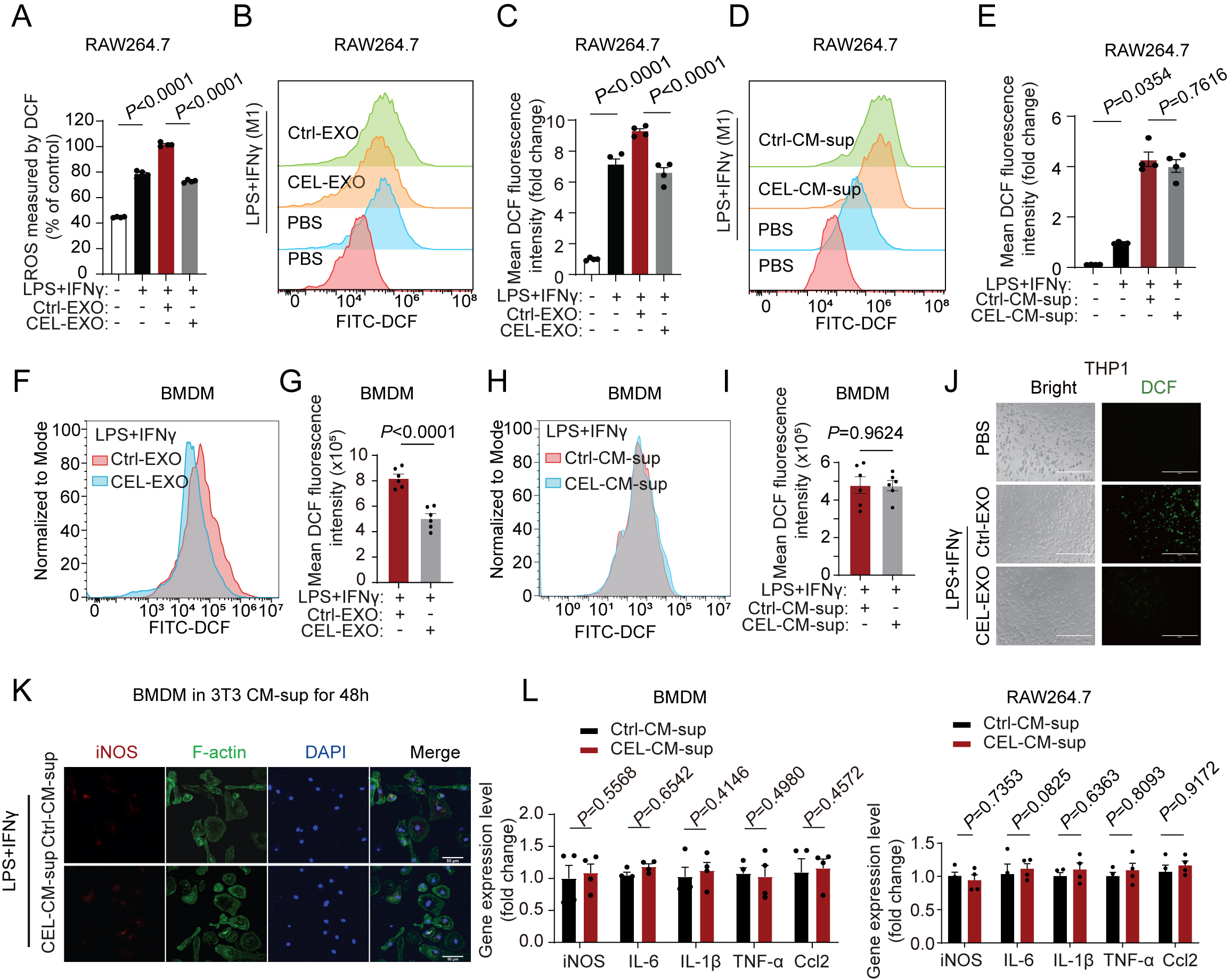


**Supplementary Figure 5. CEL-treated adipocyte-derived CM reduces ROS production and inflammatory gene expression in macrophages through exosomes.** **A.** Intracellular ROS levels in RAW264.7 cells treated with indicated EXOs (50 µg/mL, 24 h) derived from 3T3-L1 adipocytes (CEL-EXO), measured by fluorescence microplate reader (n=4). **B–C.** Flow cytometric analysis (B) and quantification (C) of ROS in RAW264.7 cells treated with indicated CEL-EXO (n=4). **D–E.** Flow cytometric analysis of ROS in RAW264.7 cells cultured in indicated CMs harvested from adipocytes with EXO depletion (CEL-CM-sup or Ctrl-CM-sup) for 24 h (n=4). **F–G.** Flow cytometric analysis (F) and quantification (G) of ROS levels in BMDMs treated with indicated EXOs for 24 h, measured by flow cytometry (n=6). **H–I**. Flow cytometric analysis (H) and quantification (I) of ROS levels in BMDMs cultured in indicated CMs harvested from adipocytes with EXO depletion for 24 h (n=6). **J.** Representative fluorescent images of ROS levels in THP1 macrophages treated with indicated EXOs for 24 h. Scale bar, 400 μm. **K.** Representive IF staining of iNOS, F-actin, and DAPI in RAW264.7 cells after 24 h CEL-CM-sup treatment. Scale bar, 50 μm. **L.** The expression analysis of M1 marker genes by RT-PCR in BMDMs and RAW264.7 cells cultured in indicated CMs harvested from adipocytes with EXO depletion (n = 4). Comparison between two groups was evaluated using an unpaired two-tailed t-test. One-way ANOVA with Tukey’s post-hoc test was used for comparing more than two groups. Data are mean ± SEM.


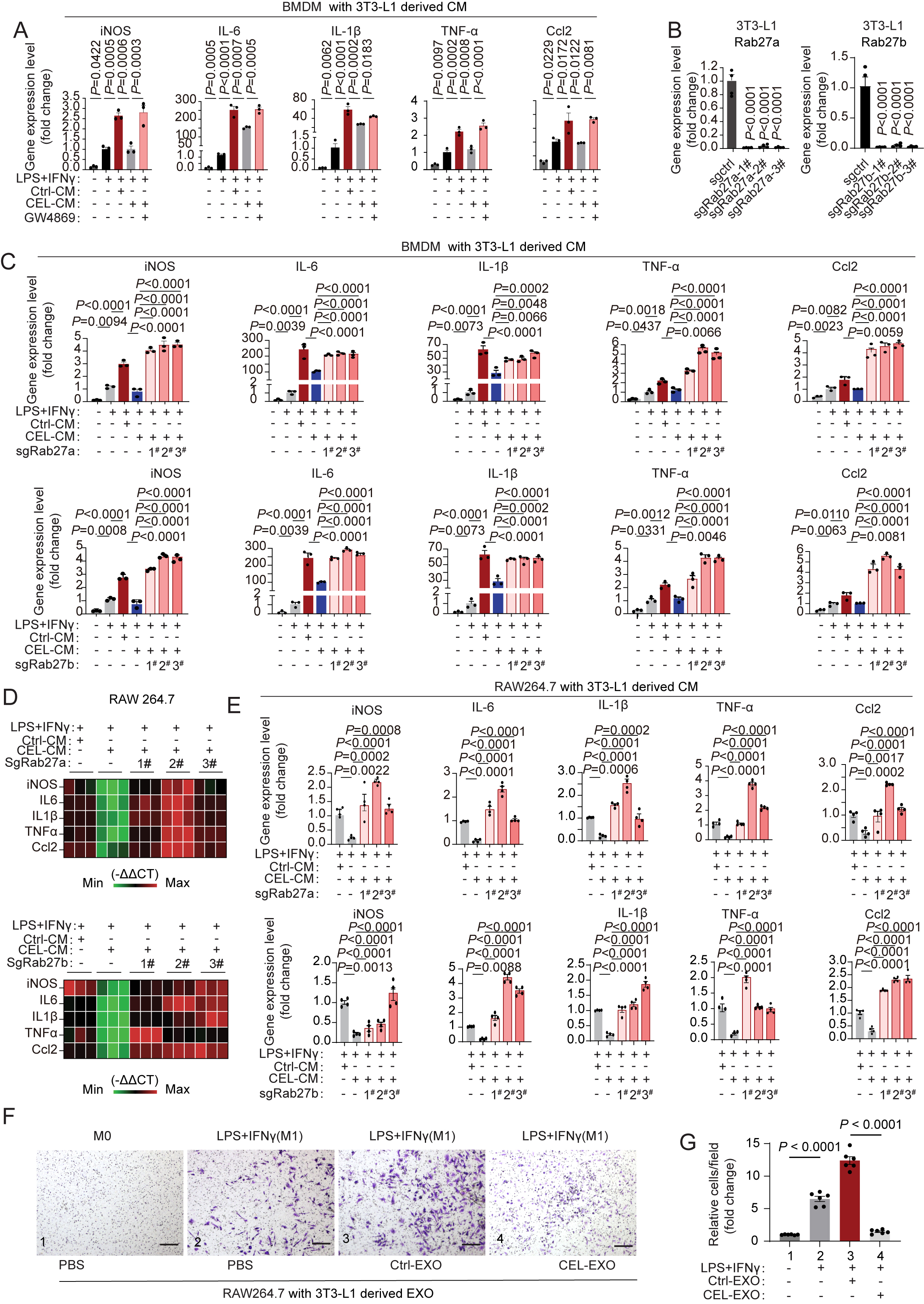


**Supplementary Figure 6. EXO secretion from adipocytes is essential to the impact of adipocyte-derived CEL-CM on macrophage polarization and migration.** **A.** The expression analysis of M1 macrophage markers by RT-PCR in BMDMs cultured in Ctrl-CM, CEL-CM or CEL-CM-GW4869 (CM harvested from 3T3-L1 adipocytes with CEL (0.4 μM, 48 h) and GW4869 (EXO inhibitor) treatment (20 μM, 24 h) treatment (for main Fig. 2I) (n=3). **B.** Knockdown efficiency of Rab27a and Rab27b in 3T3-L1 adipocytes confirmed by RT-PCR (n=4). **C.** The expression analysis of M1 macrophage markers by RT-PCR in BMDMs cultured in CEL-CM- sgRab27a (CM harvested from 3T3-L1 adipocytes with CEL treatment (0.4 μM, 48 h)) and with Rab27a knockdown) or CEL-CM- sgRab27b (CM harvested from 3T3-L1 adipocytes with CEL treatment and with Rab27b knockdown, for main Fig. 2J) (n=3). **D–E.** Heatmap (D) and quantification (E) of inflammatory gene expression in RAW264.7 macrophages cultured in CM as depicted in C (for Rab27a knockdown and for Rab27b knockdown) (n=4). **F–G.** Representative images (F) and quantification (G) of RAW264.7 macrophages migration assay with the treatment of indicated adipocyte-derived EXOs (n=6). Scale bar, 200 μm. One-way ANOVA with Tukey’s post-hoc test was used for comparing more than two groups. Data are mean ± SEM.


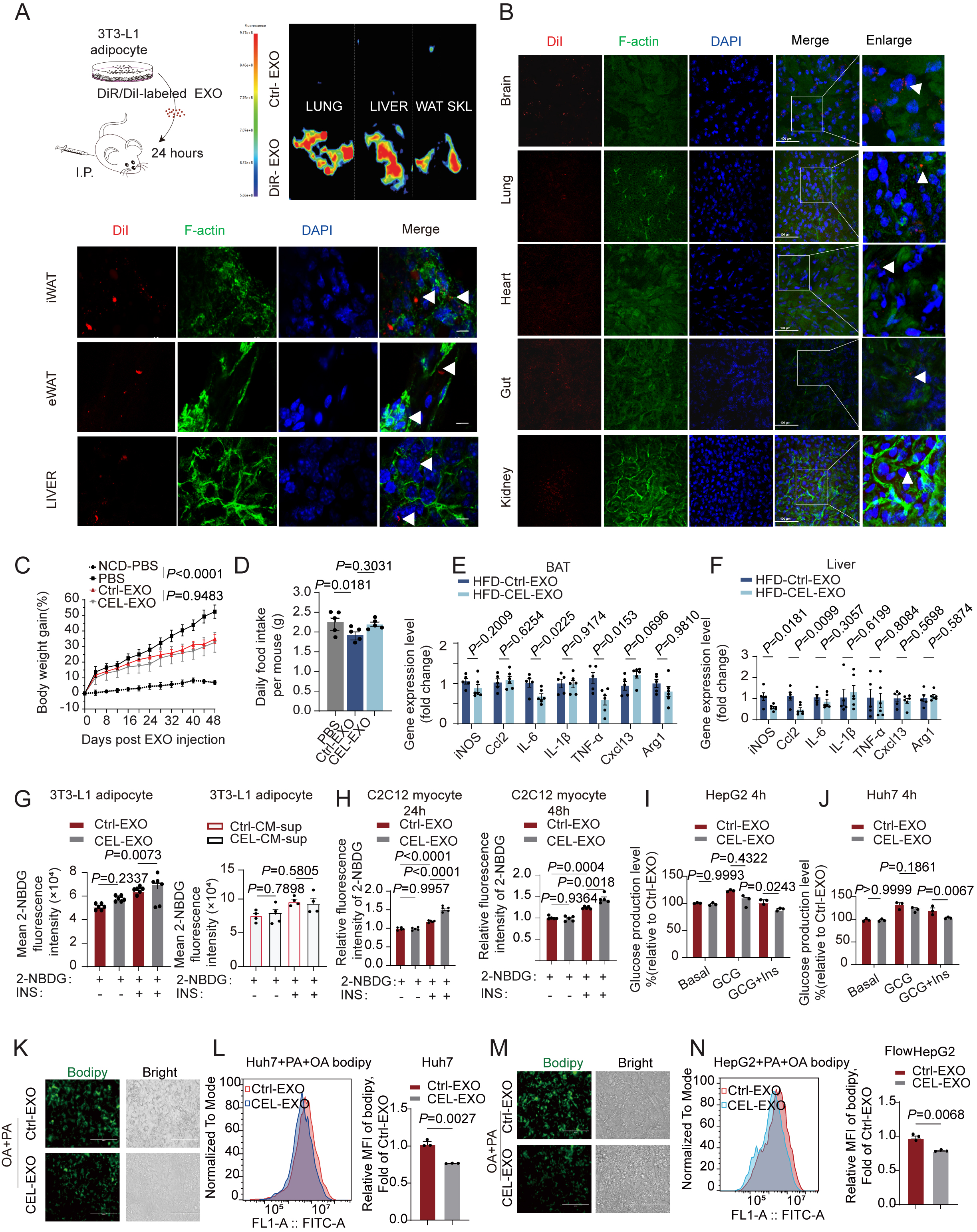


**Supplementary Figure 7. The uptake of Celastrol-treated adipocyte-derived Extracellular Vesicles (EXOs) by tissues in obese mice and evaluating the impact of CEL-EXOs on glucose metabolism.** Male C57Bl/6J mice (8 weeks) were fed a HFD for 7 weeks with administration of either Vehicle (PBS), Ctrl-EXO, or CEL-EXO (intraperitoneal injections, 50 μg/mouse, 3times/week) harvested from iWAT adipocytes with or without CEL treatment (0.4 μM, 48 h). Additionally, a control group of mice was fed a normal chow diet (NCD). These mice were utilized for the experiments A-F. **A–B**. Representative IVIS (In-vivo imaging Systems) images showing tissue distribution of DiR/DiI-labeled EXOs 24 hours after intraperitoneal injection. Signal detection observed in lung, liver, WAT, skeletal muscle, brain, heart, gut, and kidney (n=3). Scale bar for A, 10 μm, Scale bar for B, 100 μm. **C.** Body weight gain expressed as a percentage relative to day 1 post-EXO injection in mice on NCD, HFD treated with vehicle (PBS), Ctrl-EXO, or CEL-EXO derived from primary adipocytes with or without CEL treatment. (n=5-6). **D.** Daily food intake measurements during the exosome treatment period in the mice utilized in the study C (n=5-6). **E–F**. The expression analysis of M1 macrophage marker genes by RT-PCR in brown adipose tissue (BAT; **E**) and liver (**F**) isolated from obese mice with indicated treatment (n=5-6). **G-J.** Effect of the CEL-EXOs on glucose uptake in 3T3-L1 adipocytes (G) and C2C12 myocytes (H) and hepatic glucose production in Huh7 (I) and HepG2 (J) hepatocytes (n=3). **K-N.** Effect of the CEL-EXOs on lipid metabolism in Huh 7 (K-L) hepatocytes and HepG2 hepatocytes (M-N) treated with oleic acid (OA, 0.25mM) and palmitic acid (PA) (0.5mM) for 48h, Bodipy was used to visualize lipid droplets (n=3). Scale bar, 100 μm. Comparison between two groups was evaluated using an unpaired two-tailed t-test. One-way ANOVA with Tukey’s post-hoc test was used for comparing more than two groups. Data are mean ± SEM.


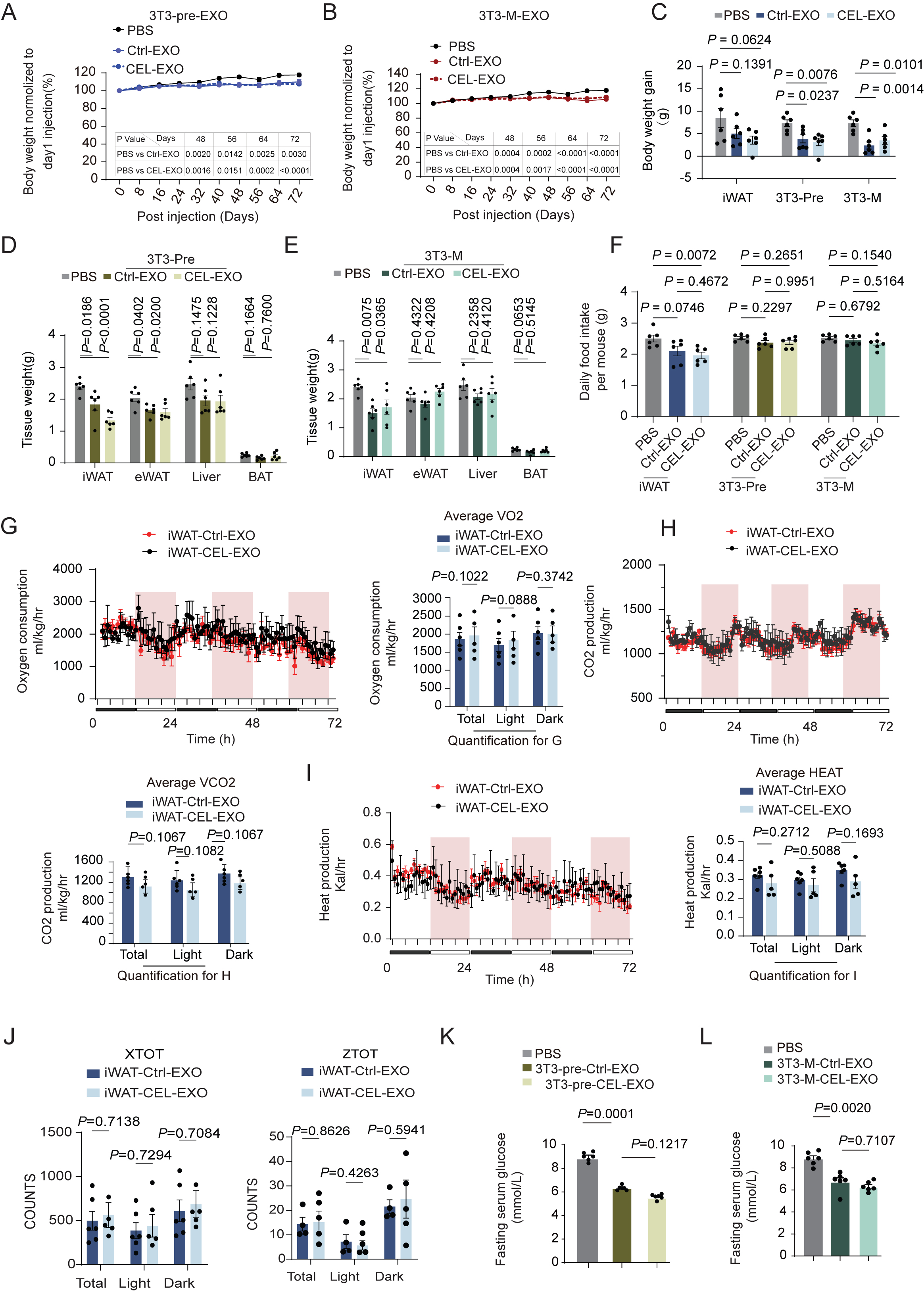


**Supplementary Figure 8. The impacts of CEL-treated adipocyte-derived exosomes on metabolic health of obese mice.**  Male C57Bl/6J mice (8 weeks) were fed a HFD for 12 weeks, followed by the administration of Vehicle (PBS), Ctrl-EXO, or CEL-EXO (Intravenous (I.V. injections, 50 μg/mouse, 3 times/week) for an additional 10 weeks. The exosomes (EXOs) used in this study were derived from iWAT SVF-differentiated adipocytes, 3T3-L1 preadipocytes (3T3-Pre), or 3T3-L1 mature adipocytes (3T3-M), with or without CEL treatment (0.4 μM, 48 h). These mice were utilized for experiments in sFig. 8 **(n=6). A-B.** Body weight changes over time of the obese mice normalized to baseline with indicated treatment (n = 6). **C.** Final body weight of obese mice with indicated treatment (n=6). **D-E.** Tissue mass of iWAT, eWAT, BAT, and liver in obese mice with treatment of indicated EXOs (n=6). **F.** Daily food intake of obese mice with indicated treatment (n = 6). **G-J.** O2 consumption (G), CO2 production (H), heat production (I) and physical activity (J) of obese mice with indicated treatment (n=5-6). **K-L.** Fasting glucose level in obese mice with indicated treatment (n=6). Comparison between two groups was evaluated using an unpaired two-tailed t-test. One-way ANOVA with Tukey’s post-hoc test was used for comparing more than two groups. Data are mean ± SEM.


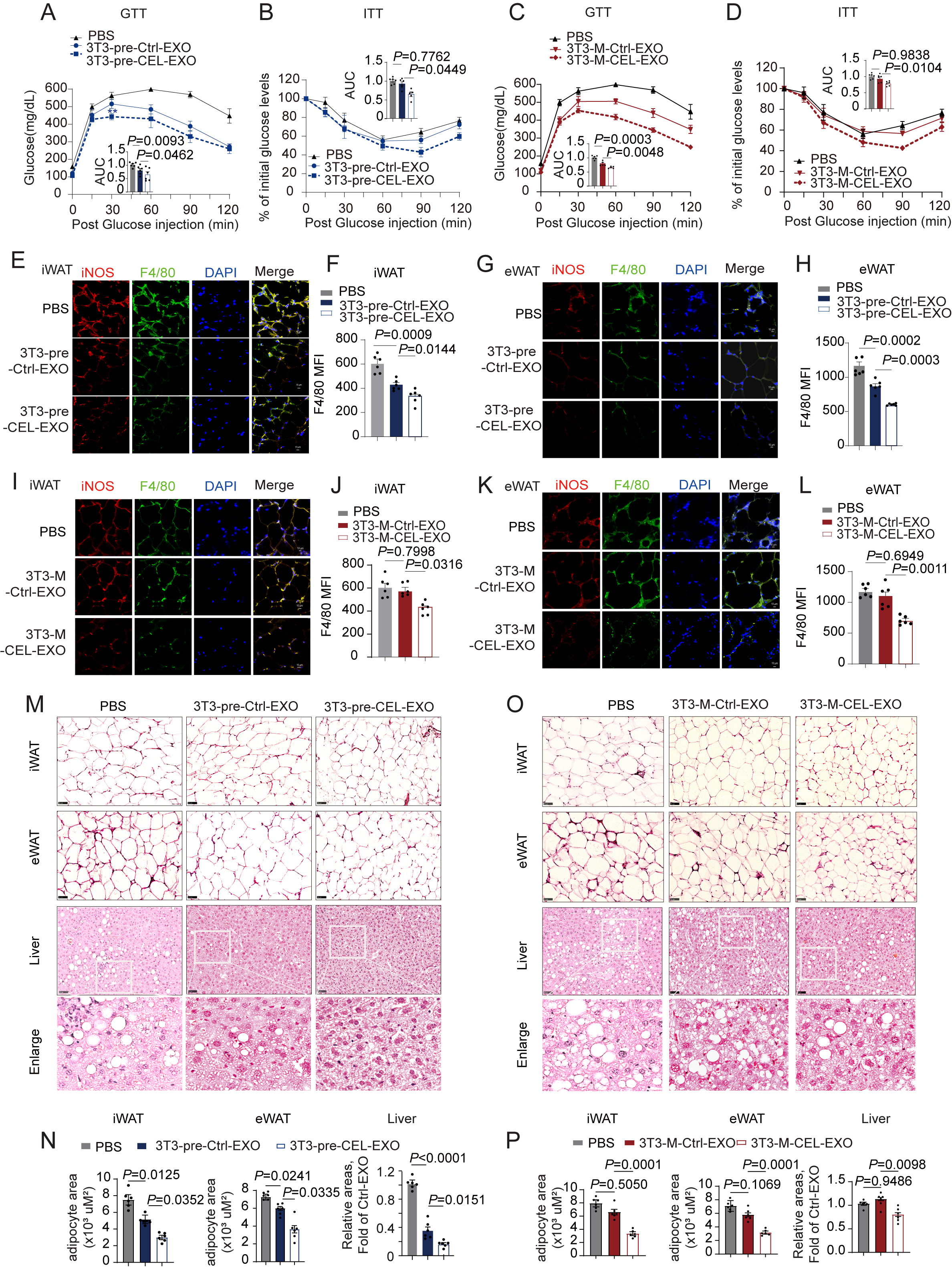


**Supplementary Figure 9. The impacts of CEL-treated adipocyte-derived exosomes on AT inflammation and adipocyte size.** Male C57Bl/6J mice (8 weeks) were fed a HFD for 12 weeks, followed by the administration of Vehicle (PBS), Ctrl-EXO, or CEL-EXO (Intravenous (I.V. injections, 50 μg/mouse, 3 times/week) for an additional 10 weeks. The exosomes (EXOs) used in this study were derived from 3T3-L1 preadipocytes (3T3-Pre), or 3T3-L1 mature adipocytes (3T3-M), with or without CEL treatment (0.4 μM, 48 h). These mice were utilized for experiments in sFig. 9. **A-D.** GTT (A, C), ITT (B, D) and associated AUC of obese mice with indicated treatment (n=5-6). **E–L.** IF staining for iNOS, F4/80, and DAPI and quantification for F4/80⁺ macrophages in iWAT (E, F, I, J) and in eWAT (G, H, K, L) of obese mice with indicated treatment. Scale bar, 10 μm (n=6). **M–P.** H&E staining for iWAT, eWAT, and liver (M, O) of obese mice with indicated treatment; quantification for adipocyte size (N-P Left) and for liver microvesicular vacuoles (N-P Right) by ImageJ (n=6). Scale bar, 50 μm. One-way ANOVA with Tukey’s post-hoc test was used for comparing more than two groups. Data are mean ± SEM.


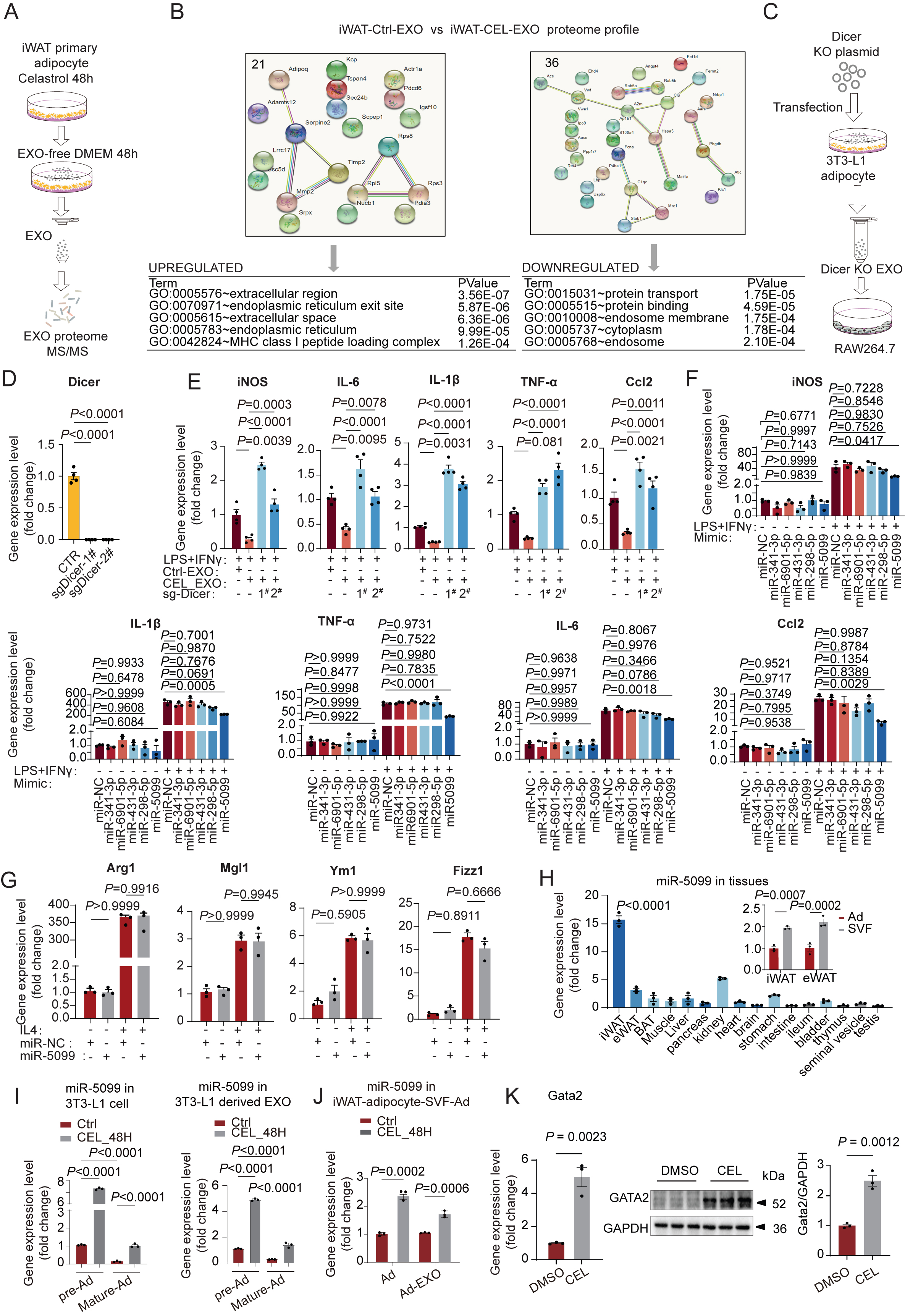


**Supplementary Figure 10. The proteomic analysis of CEL-treated iWAT SVF-differentiated adipocyte-derived EXOs and the regulation of macrophage polarization by CEL-EXOs is miRNA-dependent. A.** Experimental workflow for the preparation of iWAT-derived exosomes (iWAT-EXO) and subsequent proteomic profiling. **B.** GO analysis of differentially expressed proteins comparing iWAT-CEL-EXO versus iWAT-Ctrl-EXO; the top 5 upregulated and downregulated GO terms are displayed (n=3). **C.** Schematic workflow for generating EXOs from 3T3-L1 adipocytes with or without Dicer knockdown using sgDicer (Dicer-KO-EXO) and the subsequent treatment of RAW264.7 macrophages. **D**. Knockdown efficiency of Dicer in 3T3-L1 adipocytes confirmed by RT-PCR (n=4). **E**. The expression analysis of M1 macrophage marker genes in RAW264.7 cells treated with Ctrl-EXO or CEL-EXO, with or without Dicer knockdown, under LPS/IFN-γ( (100ng/mL; 20ng/mL)) stimulation for 24 hours (n = 4). **F**. The expression analysis of M1 macrophage marker genes in LPS/IFN-γ (10ng/mL; 20ng/mL)–stimulated BMDMs treated with selected miRNA mimics for 24 hours (n = 3). **G.** The expression analysis of M2 marker genes in IL-4(20ng/mL)-stimulated BMDMs treated with miR-5099 (50nM) mimics for 24 hours (n = 3). **H.** The relative expression level of miR-5099 in various mouse tissues, primary adipocytes (Ad) and SVF isolated from WAT (n=3). **I.** The relative expression level of miR-5099 in 3T3 preadipocytes and differentiated adipocytes without or with CEL treatment (0.4 μM, 48 h), and in preadipocyte or adipocyte-derived EXOs (n=3). **J.** The relative expression level of miR-5099 in iWAT SVF differentiated adipocytes without or with CEL treatment (0.4 μM, 48 h), and in adipocyte-derived EXOs (n=3). **K**. GATA2 mRNA and protein levels were determined by quantitative RT-PCR and WB, respectively in 3T3-L1 preadipocytes with or without CEL treatment (400nM, n=3). Comparison between two groups was evaluated using an unpaired two-tailed t-test. One-way ANOVA with Dunnet multiple comparison post hoc tests for D, H and the rest with Tukey’s post-hoc test were used for comparing more than two groups. Data are mean ± SEM.


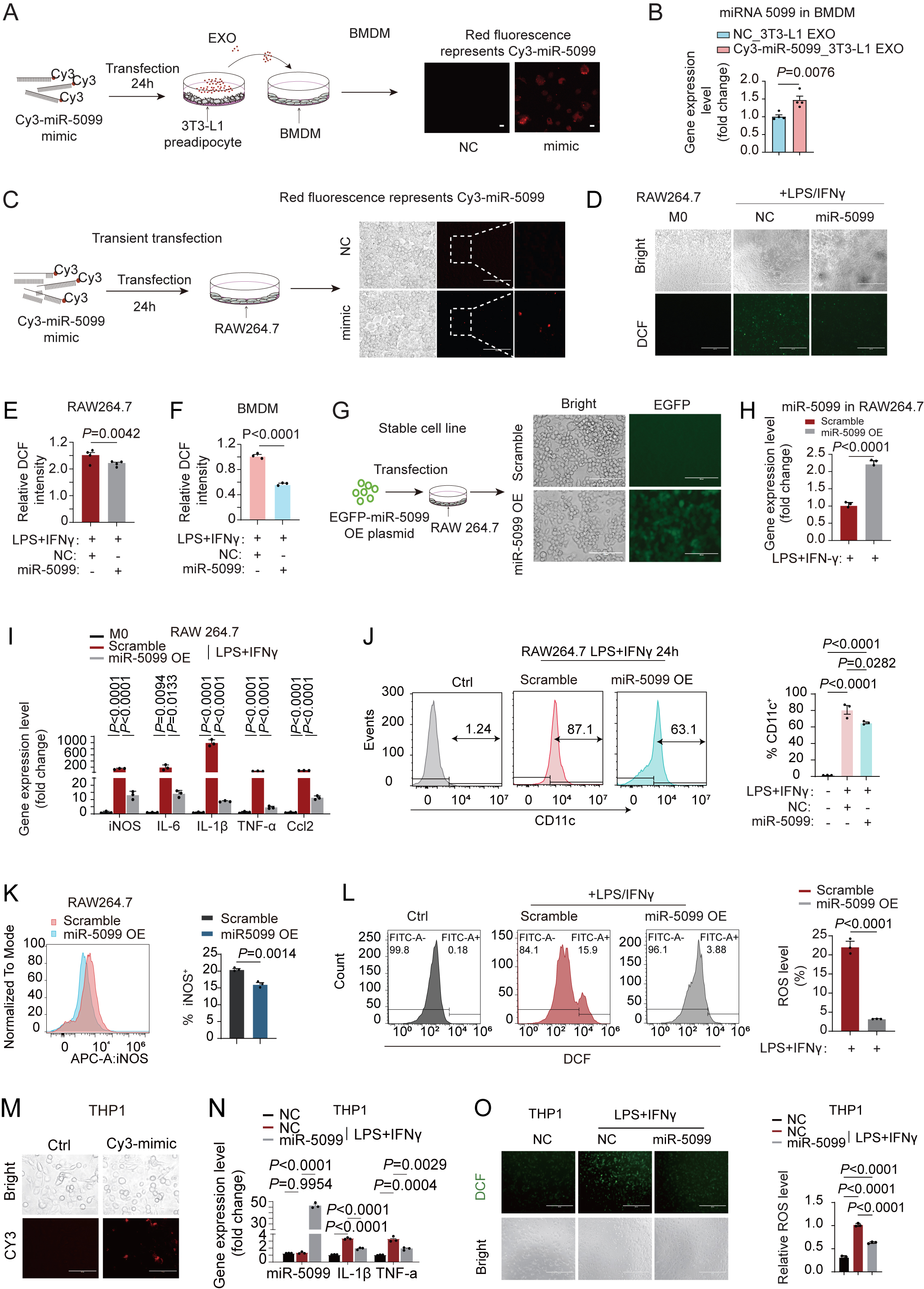


**Supplementary Figure 11. The overexpression of miR-5099 suppresses the inflammatory characteristics of macrophages. A–B**. Experimental workflow for encapsulating Cy3-labeled miR-5099 mimic into 3T3-L1 preadipcoyte derived exosomes (miR-EXO), followed by IF to confirm the uptake of Cy3-labeled exosomal miR-5099 by BMDMs (A) and RT-PCR to demonstrate the elevated miR-5099 levels in BMDMs (**B**, n = 3). Scale bar, 10 μm. **C**. Workflow illustrating the delivery of Cy3-labeled miR-5099 mimic into RAW264.7 macrophages; red fluorescence denotes Cy3-labeled miR-5099. Scale bar, 100 μm. **D-E.** Representative DCF fluorescence images illustrating ROS levels (D) and quantification using flow cytometry E) in RAW264.7 macrophages with or without miR-5099 mimic treatment (50nM, 24 h). Scale bar, 400 μm (n = 3). **F**. Quantification of DCF fluorescence signal to demonstrate ROS levels in BMDMs with or without miR-5099 mimic treatment (50nM, 24 h) (n = 3). **G-H**. Stable overexpression (OE) of miR-5099 in RAW264.7 cells using lentiviral vectors co-expressing EGFP. Workflow with representative images (G), and miR-5099 expression quantified through RT-PCR (**H**) (n=3). Scale bar, 100 μm. **I-K.** miR-5099 overexpression downregulated M1 marker gene expression (I) and decreased CD11c^+^ (J) and iNOS^+^(K) positive cells of RAW264.7 macrophages with miR-5099 OE (n=3). **L.** miR-5099 overexpression decreased intracellular ROS levels in RAW264.7 cells, validated by flow cytometry and quantification of DCF signal (n=3). **M.** IF to validate the uptake of Cy3-labeled miR-5099 by human THP1 macrophages. Scale bar, 100 μm. **N-O.** The changed level of miR-5099 and M1 macrophage markers (N) and DCF signaling (O,Scale bar, 400 μm) indicating ROS levels in human THP1 cells treated with or without miR-5099 mimic (50nM, 24h) (n=3). Comparison between two groups was evaluated using an unpaired two-tailed t-test. One-way ANOVA with Tukey’s post-hoc test was used for comparing more than two groups. Data are mean ± SEM.


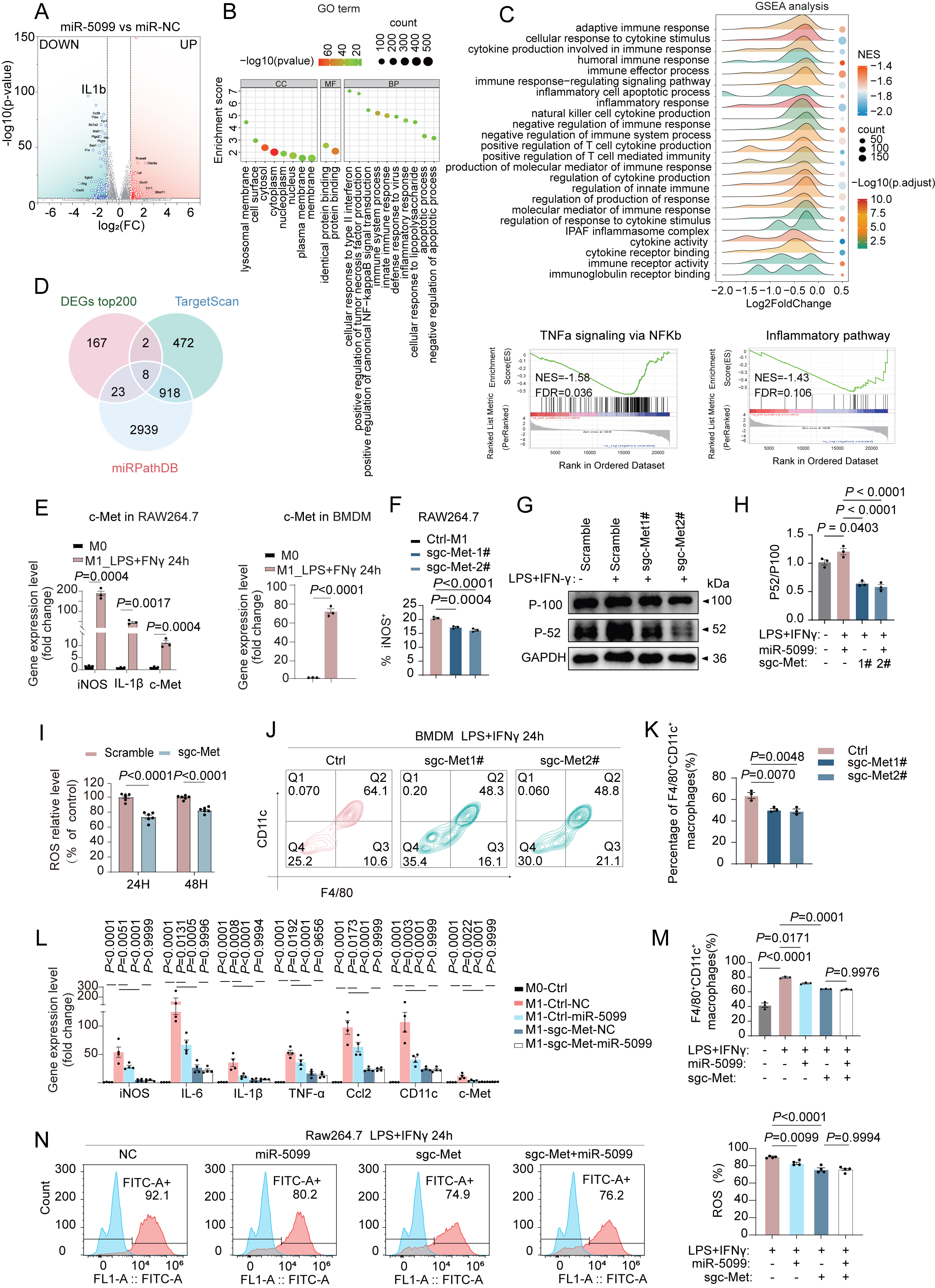


**Supplementary Figure 12. c-Met mediates the inhibitory effects of miR-5099 on inflammatory characteristics of M1 macrophages. A.** Volcano plot of RNA-seq for differentially expressed genes (DEGs) in BMDMs transfected with miR-5099 mimic or negative control (NC) (n=3). **B–C.** **G**O term enrichment analysis (B) and GSEA analysis (C) of differentially expressed genes (DEGs) in BMDMs treated with or without miR-5099 mimics, showing enrichment in inflammatory and oxidative stress pathways. **D.** A Venn diagram visualizing the potential co-regulated targets between predicted miR-5099 targets and the top 200 DEGs from RNA-seq. **E.** The expression change analysis of c-Met gene in RAW264.7 macrophages and BMDMs before and after LPS/IFN-γ (100ng/mL; 20ng/mL)stimulation for M1 polarization (n = 3). **F.** Flow cytometry analysis of iNOS expression in RAW264.7 macrophages with or without c-Met knockdown following LPS/IFN-γ treatment (100ng/mL; 20ng/mL) to induce M1 macrophage polarization for 48 hours (n = 3). **G-H.** Western blot analysis (G) and the quantification (H) of P100 and P50 proteins in RAW264.7 M1 macrophages with or without c-Met gene knockout (n=3)**. I.** Flow cytometry analysis of DCF signaling for ROS levels in RAW264.7 macrophages with or without c-Met knockdown following LPS/IFN-γ(100ng/mL; 20ng/mL) treatment to induce M1 macrophage polarization for 24 or 48 hours (n = 3). **J-K.** Flow cytometry analysis and the quantification (K) of the CD11c+/F4/80+ cell population in BMDMs with or without c-Met knockdown under following LPS/IFN-γ(10ng/mL; 20ng/mL) treatment to induce M1 macrophage polarization for 24h (n=3). **L-N.** The expression analysis of inflammatory M1 macrophage marker genes (L), flow cytometry analysis of the CD11c+/F4/80+ cell population (M) and DCF fluorescent signaling for ROS levels (N) in LPS/IFN-γ (100ng/mL; 20ng/mL)stimulated RAW264.7 macrophages treated with or without miR-5099 mimic (50nM, 24h), in combination with or without c-Met knockout (n = 3). Comparison between two groups was evaluated using an unpaired two-tailed t-test. One-way ANOVA with Dunnet multiple comparison post hoc tests for F, K, and rest with Tukey’s post-hoc test were used for comparing more than two groups. Data are mean ± SEM.


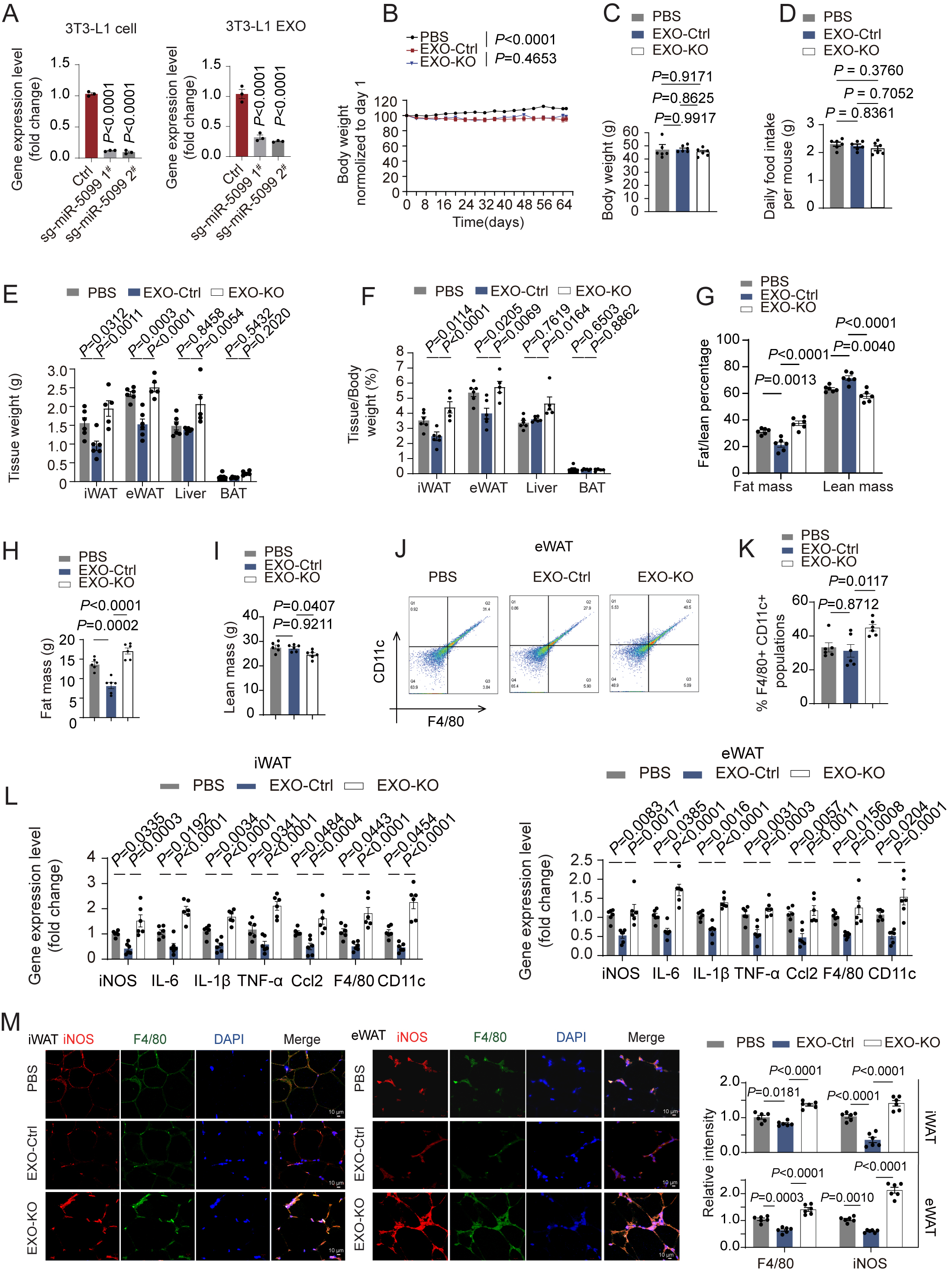


**Supplementary Figure 13. miR-5099–deficient EXOs fail to mitigate adipose tissue inflammation and obesity associated metabolic disorders of obese mice.** Male C57Bl/6J mice (8 weeks) were fed a HFD for 12 weeks, followed by the administration of vehicle (PBS), adipocyte-derived exosomes without (EXO-Ctrl) or with miR-5099 knockout (KO-EXO) derived from 3T3-L1 preadipocytes (I.V. injections, 50 μg/mouse, 3times/week) for additional 10 weeks. These mice were used for experiments in sFig.13 (n=6). **A**. The validation of miR-5099 knockout by RT-PCR in 3T3-L1 preadipocytes and secreted EXOs (n = 3). **B.** Body weight changes over time of obese mice with indicated treatment recorded during the treatment period. **C–D.** Body weight (C) and daily food intake (**D**) of indicated obese mice at the endpoint. **E–F**. Tissue mass (**E**) and tissue-to-body weight ratios (**F**) of iWAT, eWAT, liver, and BAT of obese mice. **G–I**. Body composition analysis showing total fat mass (**G, H**) and lean mass (**G, I**) in each group. **J–K**. Flow cytometric analysis (**J**) and quantification (**K**) of F4/80⁺CD11c⁺ macrophages in eWAT of indicated obese mice. **L**. The expression analysis of M1 macrophage marker genes in iWAT (left) and eWAT (right) of indicated obese mice. **M.** Representative IF images for the staining of iNOS, F4/80 and DAPI in iWAT (left) and eWAT (right) of indicated obese mice. Scale bar, 10 μm. One-way ANOVA with Dunnet multiple comparison post hoc tests for (A) and rest with Tukey’s post-hoc test were used for comparing more than two groups. Data are mean ± SEM.


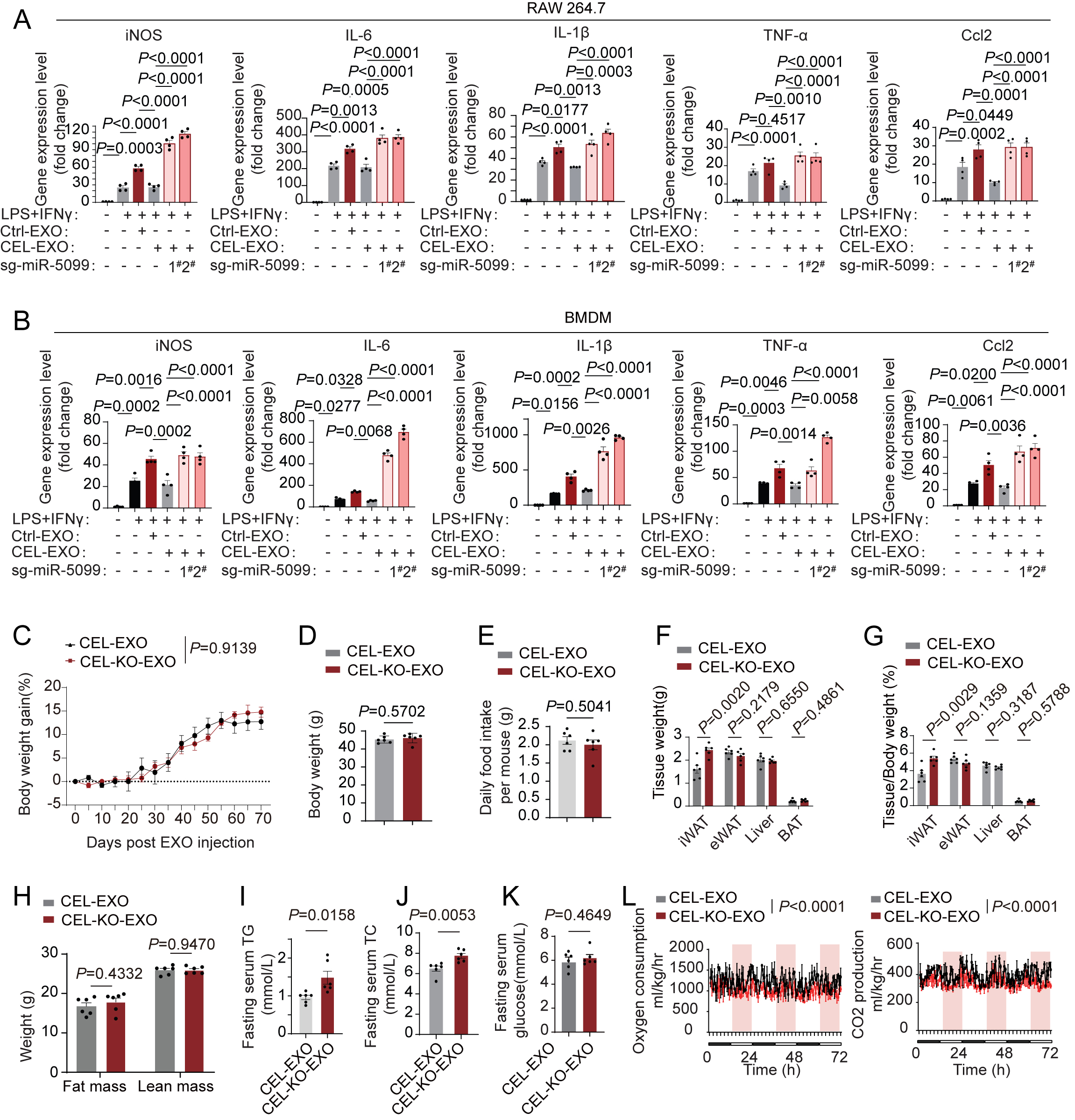


**Supplementary Figure 14. miR-5099 is essential to the beneficial impacts of CEL treated adipocyte-derived EXOs on macrophage inflammation and obesity associated metabolic complication.** Male C57Bl/6J mice (8 weeks) were fed a HFD for 12 weeks, followed by the administration of 3T3-L1 adipocyte-derived exosomes with CEL treatment without (CEL-EXO) or with miR-5099 knockout (CEL-KO-EXO) (I.V. injections, 50 μg/mouse, 3times/week) for additional 10 weeks. These mice were used for experiments in sFig. 14C-L.

**A–B.** Relative mRNA expression of inflammatory M1 macrophage marker genes in BMDMs (**A**) and RAW264.7 cells (**B**) treated with EXOs derived from Ctrl or CEL-treated 3T3-L1 adipocytes (Ctrl-EXO or CEL-EXO) without or with miR-5099 knockout for 24 h (n = 4). **C–E**. Body weight changes overtime (C), final body weight (D), and daily food intake (E) of obese mice with indicated treatment(n=6). **F-G.** Tissue mass (**F**) and tissue-to-body weight ratios (G) of iWAT, eWAT, liver, and BAT samples (n=6). **H.** Body composition analysis of total fat mass and lean mass (n=6). **I–K.** Fasting serum levels of triglycerides (**I**), total cholesterol (**J**), and glucose (K) in indicated obese mice(n=6). **L.** Whole-body oxygen consumption (left) and carbon dioxide production (right) measurement of indicated obese mice(n=6). Comparison between two groups was evaluated using an unpaired two-tailed t-test. One-way ANOVA with Tukey’s post-hoc test was used for comparing more than two groups. Data are mean ± SEM.


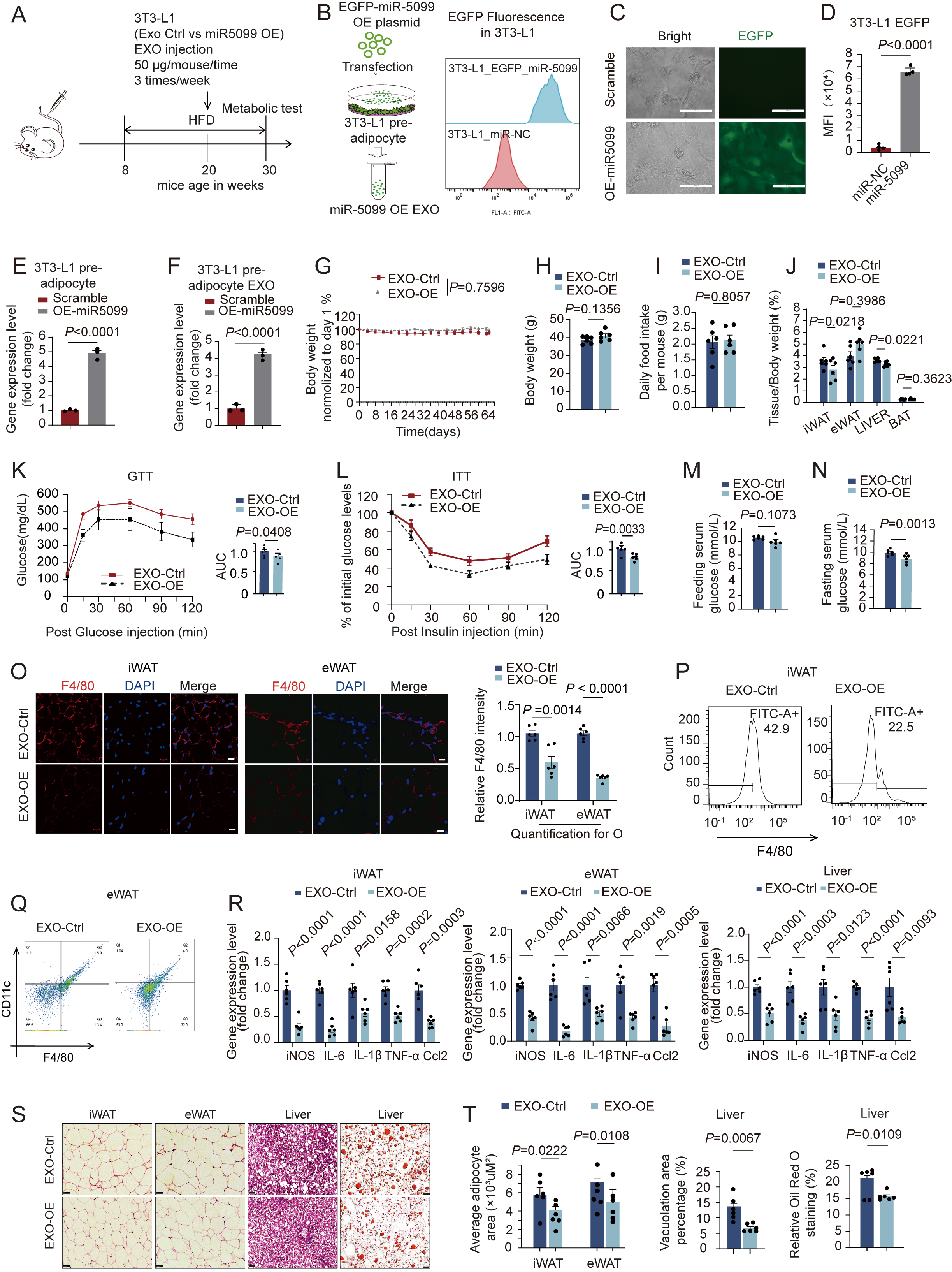


**Supplementary Figure 15. EXOs derived from adipocytes overexpressing miR-5099 alleviate adipose tissue inflammation and enhance the metabolic health of obese mice. A.** Schematic workflow illustrating in vivo delivery of EXOs. Male C57Bl/6J mice (8 weeks) were fed a HFD for 12 weeks, followed by the administration of EXOs derived from 3T3-L1 preadipocyte without (EXO-Ctrl) or with miR-5099 overexpression (EXO-OE) (I.V. injections, 50 μg/mouse, 3times/week) for additional 10 weeks. These mice were used in the experiment of G-V (n=6). Overexpressed miR-5099 was labeled using EGFP. **B-D**. Flow cytometry (B), IF (C, scale bar, 100 µm), and quantification (**D**) of EGFP signal validated miR-5099 OE (n=3). **E–F.** Using RT-PCR to validate miR-5099 OE in 3T3-L1 cells (**E**) and secreted EXOs (**F**) (n=3). **G–J**. Body weight normalized to baseline (G) and final body weight (H), daily food intake (I), and tissue-to-body weight ratios of iWAT, eWAT, BAT, and liver (J) in indicated obese mice (n=6). **K-L**. GTT(**K**) and ITT L) of obese mice with indicated treatment and associated AUCs (n=6). **M-N.** Feeding (**M**) and fasting (**N**) serum glucose levels in obese mice with indicated treatment (n=6). **O-P.** IF staining for F4/80 (O) and flow cytometry for F4/80+ macrophage population (P-Q) in iWAT and eWAT of indicated obese mice (n=6). Scale bar, 20 µm. **R.** The expression analysis of inflammatory M1 macrophage marker genes in iWAT, eWAT, and liver of indicated obese mice by RT-PCR (n=6). **S-T**. Representative H&E images of iWAT, eWAT and liver (scale bar, 50 µm), and Oil Red O staining of liver (**S**, scale bar, 20 µm) of indicated obese mice and quantification of adipocyte size and hepatic lipid accumulation (**T**) (n=6). Comparison between two groups was evaluated using an unpaired two-tailed t-test. Data are mean ± SEM.


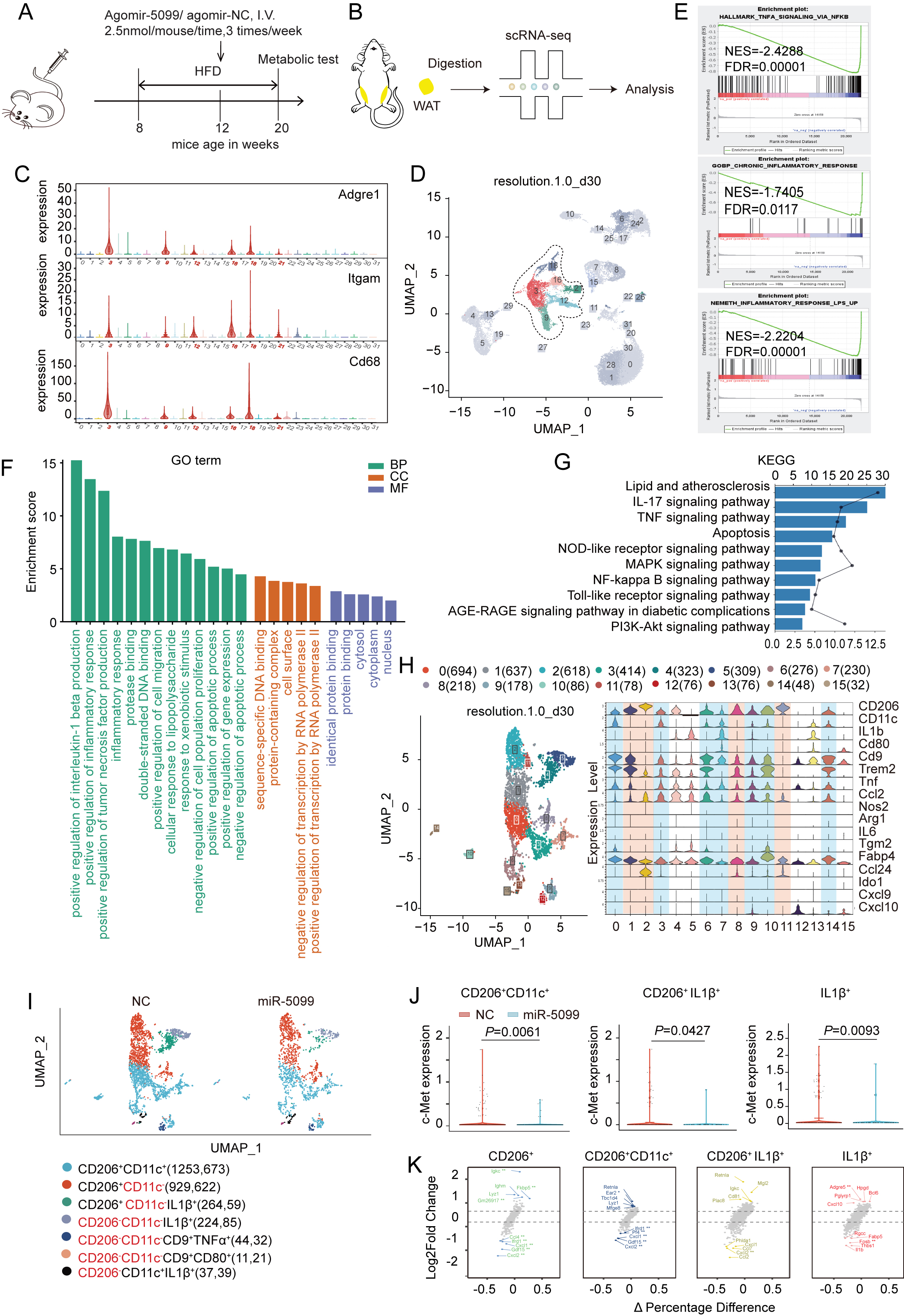


**Supplementary Figure 16. Single-cell transcriptomic analysis reveals immunomodulatory effects of miR-5099 on adipose tissue macrophages. A-B**, Schematic timeline of in vivo agomiR-5099 (miR-5099) treatment and downstream Single-cell transcriptomic analysis procedures. Male C57Bl/6J mice (8 weeks) were fed a HFD for 12 weeks, followed by the administration of agomiR-5099 (miR-5099) or agomiR-NC (NC) (I.V. injections, 2.5nmol/mouse/time, 3 times/week) for additional 8 weeks (n=6). SVFs isolated from iWAT of these mice were used for Single-cell transcriptomic analysis. **C.** Feature plots showing expression of macrophage lineage markers Adgre1, Itgam, and Cd68 in SVF cell populations. **D**. UMAP (Uniform Manifold Approximation and Projection) embedding of single-cell clusters with macrophage clusters identified as clusters 3, 9, 12, 16, 18, and 21. **E-G**. GSEA (E), GO (F) and KEGG (G) analysis of DEGs based on single-cell analysis reveal the downregulation of inflammation-related pathways in adipose tissue macrophages of obese mice treated with miR-5099. **H–I**. Re-clustering of CD11b⁺F4/80⁺CD68⁺ macrophages into 7 transcriptionally distinct subclusters (**H**) with changes in subcluster abundance (**I**) in adipose tissue macrophages of obese mice treated with miR-5099. **J**. Violin plots demonstrating reduced expression of the predicted target gene c-Met in macrophages upon miR-5099 treatment in obese mice. **K**. Downregulation of pro-inflammatory chemokines (Cxcl1, Cxcl2, etc.) across the top four macrophage subtypes in miR-5099–treated mice. Comparison between two groups was evaluated using an unpaired two-tailed t-test. Data are mean ± SEM.


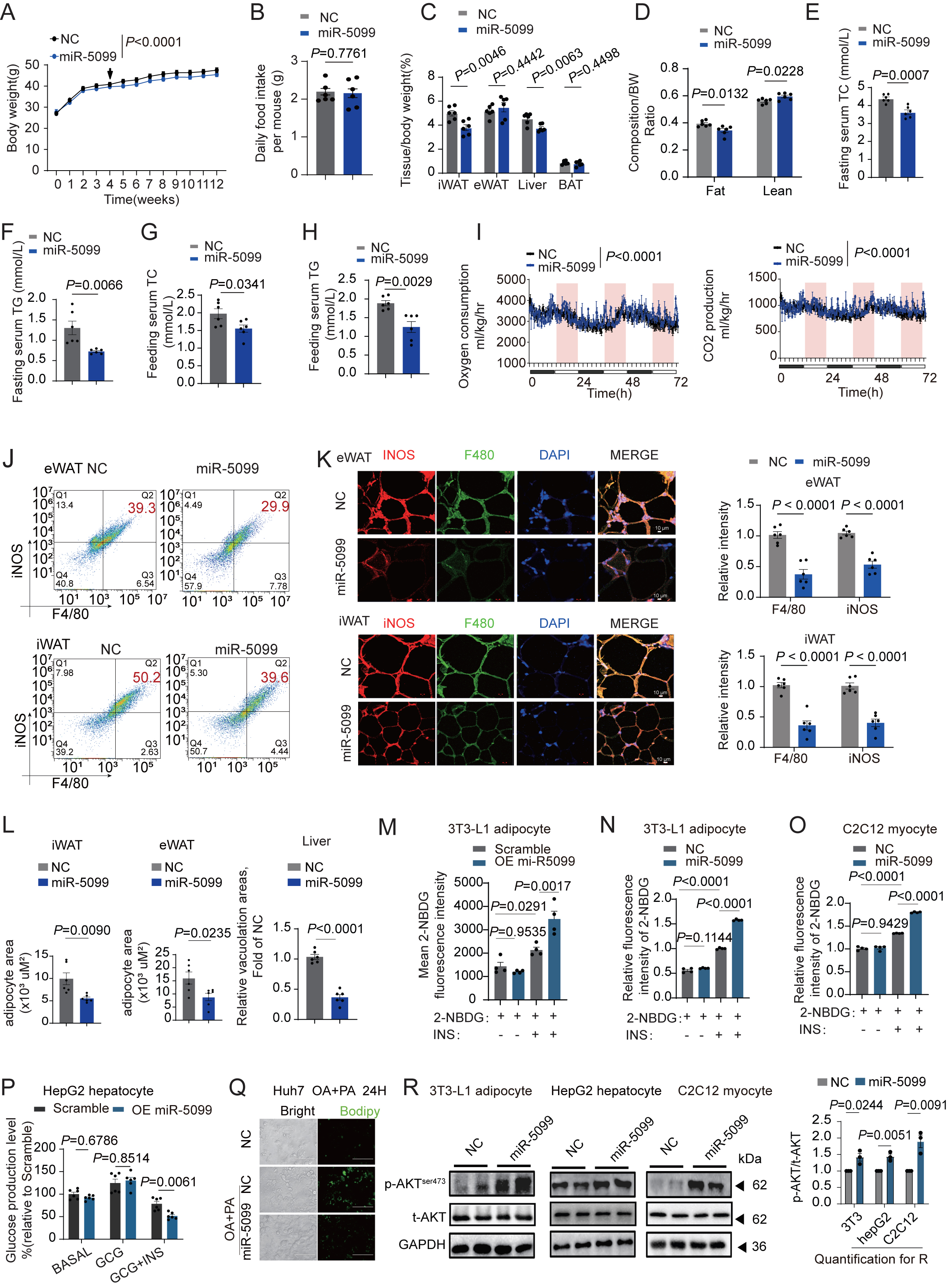


**Supplementary Figure 17.** **The effects of miR-5099 on the metabolic health of obese mice, tissue inflammation and glucose metabolism of adipocytes, myocytes and hepatocytes.** Male C57Bl/6J mice (8 weeks) were fed a HFD for 4 weeks, followed by the administration of agomiR-5099 (miR-5099) or agomiR-NC (NC) (I.V. injections, 2.5nmol/mouse/time, 3 times/week) for additional 8 weeks. These mice were used for the experiment in A-L. **A-I**. Body weight change over time, the arrow indicates the start of the injection (A), food intake (B), tissue to body weight ratios (C), body composition (D), fasting serum TC (E), fasting serum TG (F), feeding serum TC (G), feeding serum TG (H), O₂ consumption and CO₂ production (I) in indicated obese mice (n=6). **J.** Flow cytometric analysis of CD11c⁺/ F4/80⁺ macrophage population in eWAT and iWAT of indicated obese mice (for main Fig. 8N) (n=6). **K.** Representative IF staining and quantification of F4/80⁺ and iNOS⁺ in iWAT and eWAT of obese mice with indicated treatment (n=6). Scale bar, 10 μm. **L**. Quantification of adipocyte size and hepatic vacuolation for main Fig. 8Q (n=6). **M–O.** The effects of miR-5099 OE on glucose uptake in 3T3-L1 adipocytes (M) and the effects of miR-5099 mimic on glucose uptake in 3T3-L1 adipocytes (N) and C2C12 myocytes (O) (n=3). **P–Q**. Glucose production in HepG2 hepatocytes (P) and lipid accumulation (Q, Scale bar, 100 μm) in Huh7 hepatocytes with PA+OA stimulation (n=3). **R. Phosphorylated** AKT and total AKT level measurement by WB and quantification of pAKT/AKT ratio in 3T3-L1 adipocytes, C2C12 myotubes, and HepG2 hepatocytes following miR-5099 treatment (n=3). Comparison between two groups was evaluated using an unpaired two-tailed t-test. One-way ANOVA with Tukey’s post-hoc test was used for comparing more than two groups. Data are mean ± SEM.
